# Evaluating the association between household water, sanitation and hygiene (WASH) and selected placenta-related complications in The Gambia, Kenya and Mozambique

**DOI:** 10.1371/journal.pgph.0005004

**Published:** 2026-10-15

**Authors:** Joseph Wells, Joseph Waiswa, Anifa Vala, Bahtisen Golla, Grace Mwashigadi, Hawanatu Jah, Marleen Temmerman, Robin Okello, Isaac Mwaniki, Herminio Cossa, Moses Mukhanya, Esperança Sevene, Yahaya Idris, Onesmus Wanje, Angela Koech, Anna Roca, Laura Braun, Marie-Laure Volvert, Fatima Touray, Umberto D’Alessandro, Peter von Dadelszen, Wendy Graham, Hannah Blencowe, the PRECISE and PRECISE-DYAD Networks

**Affiliations:** 1 Department of Disease Control, London School of Hygiene and Tropical Medicine, London, United Kingdom; 2 Centre for Maternal, Reproductive, Adolescent and Child Health, London School of Hygiene and Tropical Medicine, London, United Kingdom; 3 Centro de Investigação em Saúde de Manhiça, Manhiça, Mozambique; 4 Centre of Excellence in Women & Child Health, Aga Khan University, Nairobi, Kenya; 5 MRC Unit The Gambia at the London School of Hygiene and Tropical Medicine, Fajara, The Gambia; 6 Department of Physiological Science, Clinical Pharmacology, Faculty of Medicine, Eduardo Mondlane University, Maputo, Mozambique; 7 Department of Women and Children’s Health, School of Life Course and Population Sciences, Faculty of Life Sciences and Medicine, King’s College London, London, United Kingdom; 8 Department of Obstetrics and Gynaecology and BC Children’s Hospital Research Institute, University of British Columbia, Vancouver, Canada; 9 Department of Infectious Disease Epidemiology and International Health, London School of Hygiene & Tropical Medicine, London, United Kingdom; PLOS: Public Library of Science, UNITED STATES OF AMERICA

## Abstract

Placenta-related complications contribute substantially to maternal and perinatal mortality globally, with the greatest burden in low- and middle-income countries (LMICs). However, the potential influence of inadequate household water, sanitation and hygiene (WASH) on these outcomes remains poorly characterised. We investigated associations between household WASH conditions and selected placenta-related complications, including pre-eclampsia, small-for-gestational-age (SGA) and stillbirth, in The Gambia, Kenya and Mozambique. This secondary analysis was nested within PRECISE (PREgnancy Care Integrating Translational Science, Everywhere), a prospective observational cohort of 5,745 unselected pregnant women. Multivariable modified Poisson regression with robust standard errors was used to assess associations between household WASH exposures and pre-eclampsia, SGA or stillbirth. Missing exposure and covariate data were low (<5%) and handled using multiple imputation. Compared with water piped into the dwelling or plot, other improved water sources (adjusted relative risk [aRR] 1.18, 95% confidence interval [CI] 1.07–1.31, p = 0.002) and unimproved water sources (aRR 1.23, 95% CI 1.00–1.50, p = 0.049) were associated with a higher risk of any selected placenta-related complication. Unimproved sanitation facilities, compared with improved facilities, were also associated with increased risk (aRR 1.15, 95% CI 1.02–1.30, p = 0.019). The findings suggest that household‑level WASH conditions may be associated with selected placenta‑related complications through both direct and indirect pathways. Water sources, whether unimproved or improved, may be contaminated, and collection, transport, and storage further increase contamination risks while adding a physical burden to water collectors. Unsafe sanitation may also increase exposure to enteric pathogens. Given the observed associations, targeted WASH interventions warrant investigation as a potential strategy to reduce placenta-related complications and improve maternal and neonatal health outcomes in LMICs. Future research should explore the mechanisms linking WASH to pre-eclampsia, SGA, stillbirth and other placenta-related complications, and assess the effect of comprehensive WASH interventions.

## Introduction

Placenta-related complications, such as pre-eclampsia, small-for-gestational-age (SGA) and stillbirth, contribute to approximately 46,000 maternal deaths and 2.5 million fetal, neonatal, and infant deaths globally each year [1]. The majority (99%) of these deaths occur in low- and middle-income countries (LMICs), with over half taking place in sub-Saharan Africa [1]. A range of factors have been associated with these complications. However, in many LMICs, the complete array of risk factors for these conditions remains poorly understood.

One pathway of interest is the potential increased risk of placenta-related complications in women exposed to inadequate household water, sanitation and hygiene (WASH) conditions. Poor household- and community-level WASH conditions are well-established determinants of various adverse health outcomes, particularly diarrhoeal diseases. Improved WASH infrastructure can help protect against diarrhoeal pathogens and reduce exposure to soil-transmitted helminths, acute respiratory infections and the risks associated with chemical contaminants in water [2–4].

Exposure to poor WASH conditions during pregnancy can have both direct effects, such as acute maternal gastrointestinal infection or exposure to chemical contaminants through drinking unsafe water, as well as indirect effects, such as increased physical exertion from water collection [4]. Enteric infections and urinary tract infections can provoke systemic inflammation and endothelial dysfunction implicated in abnormal placentation and pre‑eclampsia [5,6]. Chronic enteric exposure can also lead to environmental enteric dysfunction (EED) and malabsorption, compromising fetal growth [5–10]. Severe WASH‑related infections, such as cholera, are linked to fetal loss via maternal dehydration and reduced placental perfusion, while listeriosis causes transplacental infection that can result in stillbirth [6,9–11]. Inorganic compounds (e.g., arsenic) have also been associated with low birth weight (LBW), SGA and stillbirth [12–17]. Limited or no handwashing facility can also increase the risk of respiratory infections, such as influenza or severe acute respiratory syndrome coronavirus 2 (SARS-CoV-2), which have been linked to pre-eclampsia and stillbirth [18–22]. WASH access constraints often require women to walk long distances, frequently in high ambient temperatures, to collect water. Heat strain, dehydration and haemodynamic stress may reduce uteroplacental perfusion, consistent with epidemiological links between heat exposure and LBW and stillbirth [23–29]. Despite biological plausibility and supportive epidemiological evidence across related exposures, few studies have directly examined household-level WASH conditions in relation to pre-eclampsia, SGA, and stillbirth.

This secondary analysis of the PRECISE (PREgnancy Care Integrating Translational Science, Everywhere) cohort examined household WASH characteristics among pregnant women in The Gambia, Kenya, and Mozambique and their associations with a composite of selected placenta-related complications comprising pre-eclampsia, SGA and stillbirth [30]. National data from these three countries indicate poor WASH infrastructure in households. In The Gambia, 41% of households rely on unimproved sanitation facilities, such as pit latrines without a slab or bucket latrines, or practice open defecation. In Kenya, this figure is 38%, while in Mozambique it is 58% [31]. Additionally, many households in these countries use unimproved water sources, such as unprotected wells or springs, or surface water. In The Gambia, this figure is 9%, in Kenya, 28% and in Mozambique, 27% [31]. By assessing whether household WASH characteristics are associated with pre-eclampsia, SGA and stillbirth, we aim to enhance understanding of potential household-level determinants of these complications. Such evidence could inform the design and evaluation of targeted interventions to improve household-level WASH access for pregnant women, potentially reducing the incidence of placenta-related complications and the consequences for mothers and newborns.

## Methods

### Ethics statement

The PRECISE and PRECISE-DYAD studies were reviewed and approved by the King’s College London Research Ethics Committee, United Kingdom (approval references HR-17/18–7855 and HR-20/21-19714); The Gambia Government/Medical Research Council Joint Ethics Committee, The Gambia (approval references SCC 1619 and SCC 22843); the Aga Khan University Hospital Research and Ethics Committee, Nairobi, Kenya (approval references 2018/REC-74 and 2021/IERC-08); the Centro de Investigação em Saúde de Manhiça Institutional Bioethics Committee, Manhiça, Mozambique (approval reference CCI/044/OUT/2024); and the Mozambique Ministry of Health National Bioethics Committee for Health (Comité Nacional de Bioética para a Saúde, CNBS), Maputo, Mozambique (approval references 545/CNBS/18 and 655/CNBS/20). Ethical approval was granted by all committees. Written informed consent was obtained from all participants prior to their enrolment in the study.

### Inclusivity in global research

Additional information regarding the ethical, cultural, and scientific considerations specific to inclusivity in global research is included in the supporting information (S1 Checklist).

### Study design

This study is a secondary analysis nested within the PRECISE prospective observational cohort, exploring selected placenta-related complications, including pre-eclampsia, SGA and stillbirth in The Gambia, Kenya and Mozambique. Household WASH exposures were measured at enrolment, and selected placenta-related complications were recorded through follow-up and assessed at birth. This was a collaboration with King’s College London, the Medical Research Council (MRC) Unit The Gambia at the London School of Hygiene and Tropical Medicine (LSHTM), the Aga Khan University in East Africa, and the Centro de Investigação de Saúde de Manhiça in Mozambique [30,32,33].

Recruitment to the PRECISE cohort occurred between 18^th^ June 2019 and 2^nd^ July 2021 in The Gambia, 24^th^ June 2019 and 6^th^ December 2022 in Kenya and 2^nd^ July 2019 and 16^th^ November 2021 in Mozambique. There was a pause in recruitment (and follow-up in The Gambia and Kenya) from April to July 2020 due to the COVID-19 pandemic [30].

### Study sites

In The Gambia, the study was conducted in the district of Farafenni, Central Gambia, on the trans-Gambian road linking Dakar with Ziguinchor, in southern Senegal. The recruiting centres were Farafenni District Hospital (urban) and two rural primary health care facilities in Illiasa and Nyagen Sanjal. In Kenya, recruiting centres were Mariakani Subcounty Hospital (urban) in Kaloleni sub-county, and Rabai Subcounty Hospital (rural) in Rabai sub-county. Both hospitals are in Kilifi County in coastal Kenya. In Mozambique, recruiting centres were Manhiça District Hospital (primarily urban population) and Xinavane Rural Hospital (primarily rural population) in Maputo Province, Southern Mozambique.

### Study population

The PRECISE prospective pregnancy cohort enrolled unselected pregnant women who attended participating health facilities for their own antenatal care, aged 16–49 years, and had not been referred from another facility, regardless of gestational age or risk of complications [32,33]. Women were excluded if they declined participation, were not pregnant, or were aged less than 16 or more than 49 years [33].

For this particular analysis, women were included if they had:

- Enrolled as an unselected pregnant participant in PRECISE
- Completed study follow‑up through to delivery
- Available household WASH data

No additional restriction was applied on the basis of baseline pregnancy risk or complications identified at enrolment. This approach was chosen because PRECISE was designed as an unselected antenatal cohort, allowing assessment of household WASH exposures in a broadly representative population of pregnant women attending routine antenatal care at the study sites.

### Data collection

Enrolment took place at the first antenatal care (ANC) visit (median 20.9 weeks of pregnancy (interquartile range (IQR): 16.1, 25.4)). Information on baseline demographics, anthropometry and WASH variables was collected at enrolment. Data regarding selected placenta-related complications (pre-eclampsia, SGA or stillbirth) were collected at birth (Fig 1). Household WASH characteristics were collected once at enrolment and analysed as baseline exposure measures. Repeat WASH measurements during pregnancy were not available. Therefore, changes in household WASH conditions after enrolment could not be assessed. Descriptions of how data were collected or calculated, for example, gestational age, can be found in the PRECISE protocol (S1 File).

**Fig 1:**
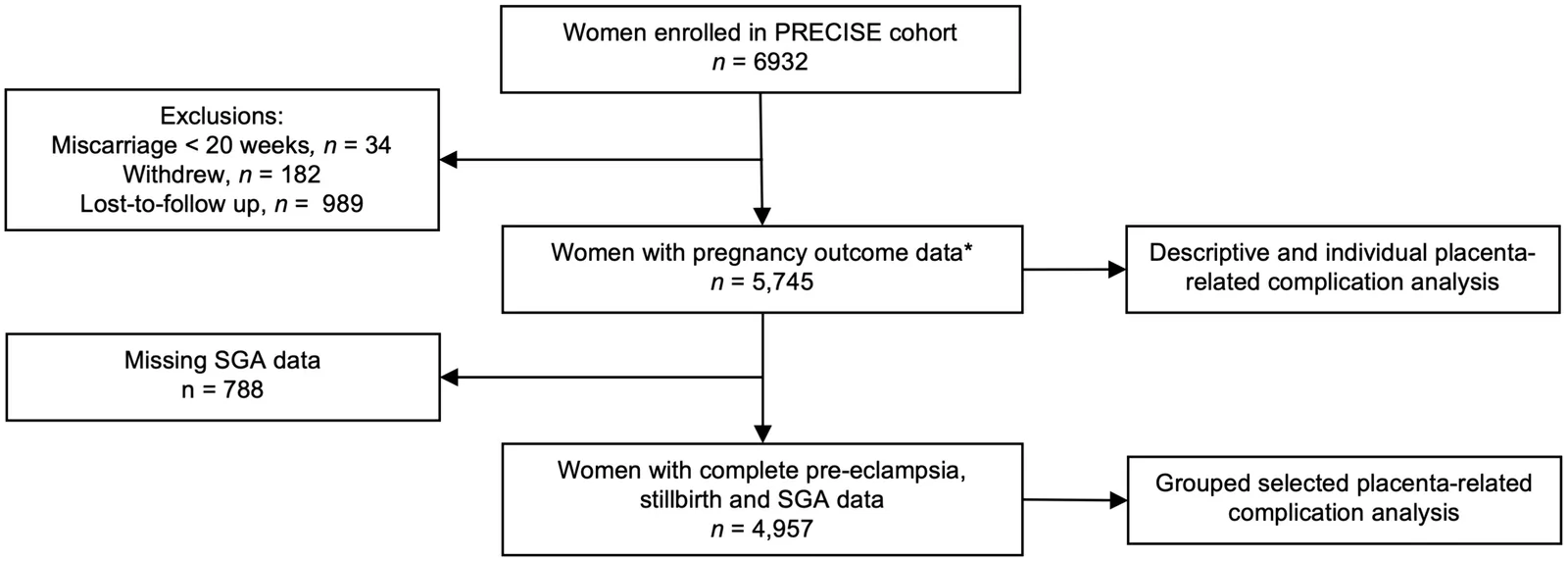
Participant flow diagram included from the PRECISE cohort. * Eighteen pregnancy outcomes could not be classified but were included in this analysis [30].

### Sample size calculation and post hoc statistical power assessment

The PRECISE cohort sample size was originally calculated to ensure recruitment of sufficient women with placental disorders for analyses of maternal risk factors [32,33]. No formal sample‑size calculation was undertaken specifically for this analysis; therefore, a post‑hoc power assessment was conducted. This assessment was based on the 4,957 participants with complete classification of the composite outcome, among whom the prevalence of any selected placenta-related complication was 27%. Statistical power was estimated for relative risks (RRs) of 1.1–1.5 across exposure prevalences of 10–50%, using two‑sample comparisons of proportions with a two‑sided α of 0.05. This assessment provides an approximate indication of the associations detectable under the specified assumptions but does not represent the power of the final multivariable models (S1 Fig).

### Data categorisations

#### Exposure variables.

A conceptual framework was developed based on existing literature to highlight the potential relationships between WASH variables collected in the PRECISE study and selected placenta-related complications (Fig 2) [4–29].

**Fig 2:**
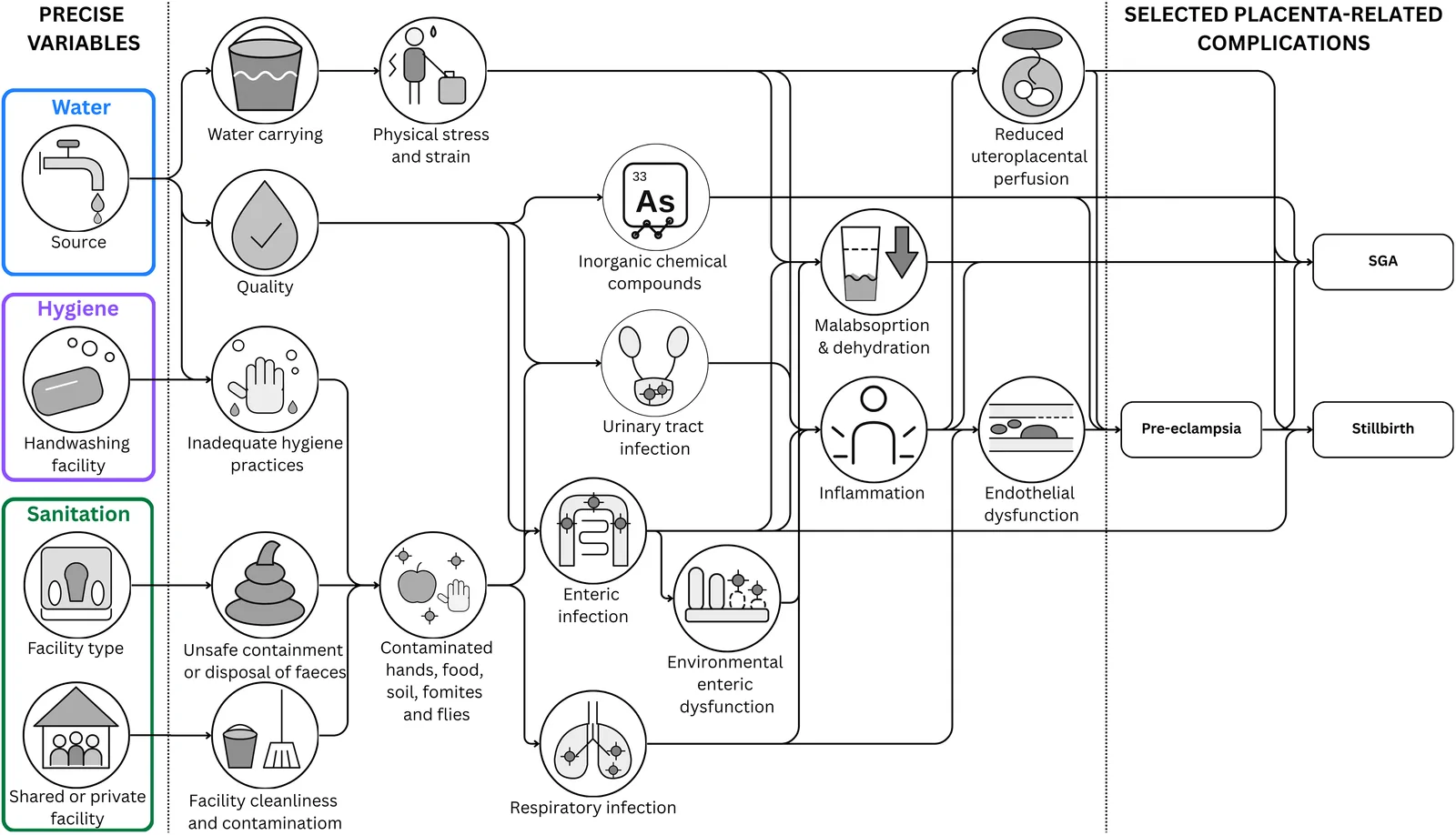
PRECISE-specific WASH exposures and potential pathways linking them to selected placenta-related complications. The icons were generated using Claude (Anthropic), from text descriptions of each concept provided by the authors. The authors reviewed all icons for conceptual accuracy and assembled the final figure. Abbreviations: PRECISE, PREgnancy Care Integrating translational Science, Everywhere; SGA, small-for-gestational-age; WASH, Water, sanitation and hygiene.

PRECISE WASH variables were categorised a priori to improve interpretability, with all categorisation decisions made before analysis (Fig 3). Water sources and sanitation facilities were categorised into improved and unimproved using the WHO/UNICEF Joint Monitoring Programme (JMP) definitions (Fig 3) [31]. Improved water sources were further subdivided into piped water into the dwelling/plot and other improved water sources, as this distinction captures whether water is available on premises, an important component of the JMP “safely managed” service ladder and an indicator of time savings, convenience, improved water quantity and quality and reduced water insecurity [31]. However, the data available do not allow us to determine whether supplies were consistently available when needed or free from faecal and chemical contamination (Fig 3) [31].

**Fig 3:**
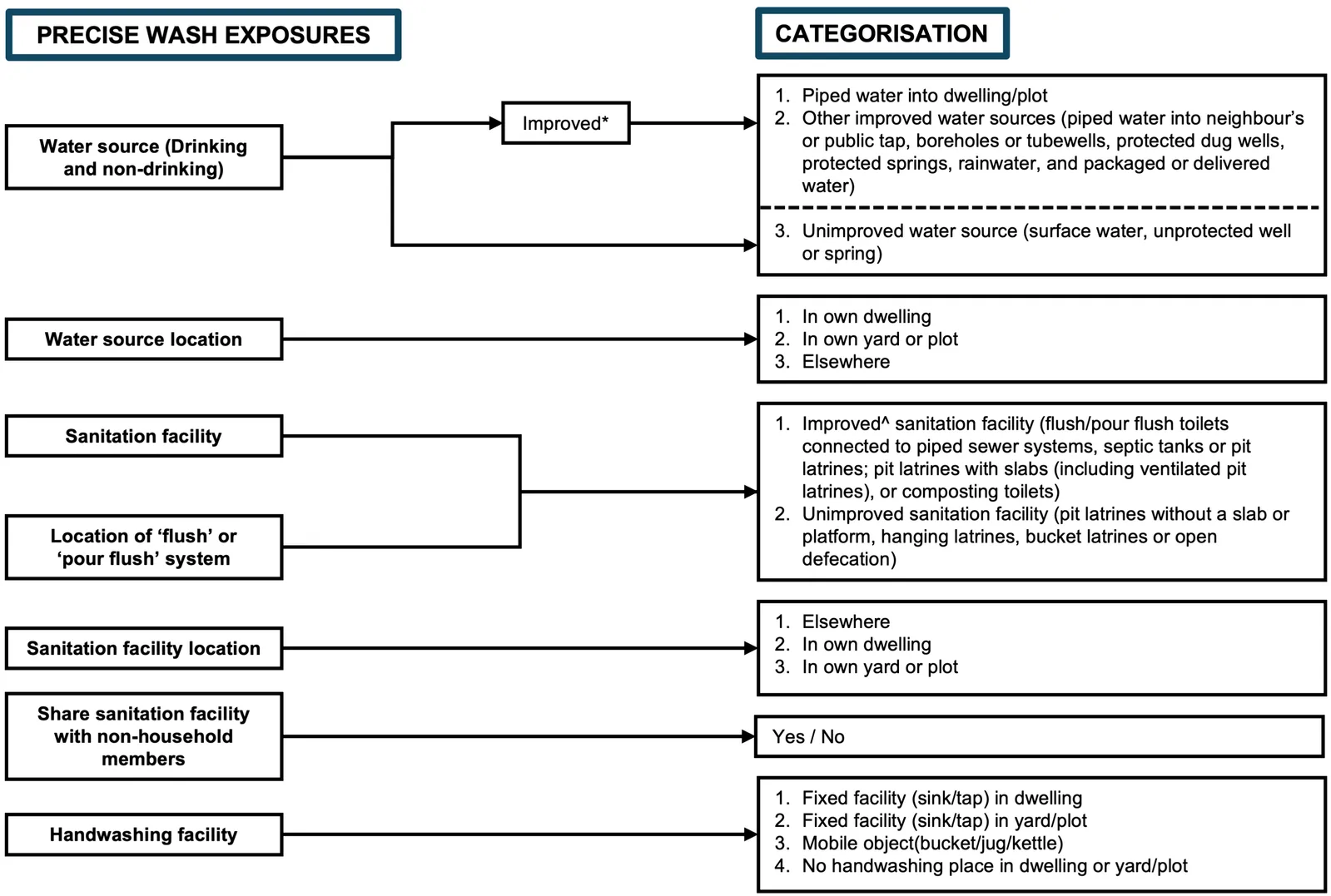
Water, sanitation and hygiene (WASH) survey questions in the PRECISE study and categorisations. * Improved drinking-water sources are defined as those more likely to be protected from outside contamination and faecal material. The improved water source variable was subdivided into piped water into dwelling/plot and “other improved sources” for further interpretability. ^ Improved sanitation facilities separate human waste from human contact.

WASH practices were also categorised into the JMP service levels, used globally for the monitoring of access, availability, and quality of water, management of excreta from households, and the conditions and practices related to hygiene. These service levels were compared with the national data from the 2022 JMP global database [31]. JMP data used for comparisons can be found in the supplementary material (S1 Data).

#### Outcome.

A binary variable was created to identify whether a woman experienced one or more selected placenta‑related complications. These complications were defined as follows:

- **Pre-eclampsia** was defined according to the International Society for the Study of Hypertension in Pregnancy criteria as: hypertension (systolic blood pressure ≥140 mmHg or diastolic blood pressure ≥90 mmHg) and at least one of:
  1. Symptoms: abdominal pain, headache, visual, chest pain, shortness of breath
  2. Signs: oxygen saturation (pulse oximetry) <90%, eclampsia, seizure, blindness, stroke, or pulmonary oedema
  3. Laboratory tests: thrombocytopaenia, or elevated serum creatinine, alanine transaminase, or aspartate transaminase
  4. Significant proteinuria (≥ ++ dipstick, urinary protein:creatinine ratio >30 mg/mmol)
  5. Evidence of uteroplacental dysfunction: fetal death, stillbirth, placental abruption, or birthweight <5^th^ centile for gestational age [34]
- **SGA:** < 10th centile for sex and gestational age, using the INTERGROWTH standards [35]
- **Stillbirth:** Baby born without vital signs at ≥20 + 0 weeks’ gestation or ≥500 g [36]

Pre-eclampsia, SGA and stillbirth were combined as a composite because each may involve placental dysfunction, while recognising that the outcomes have distinct pathophysiological pathways and frequencies. The composite was therefore used to assess whether WASH exposures are associated with the occurrence of any selected placenta-related complication, while each component was also analysed separately in sub-group analysis.

Birth outcomes (still- or livebirth) were complete for all women, and minimal missing data (0.1%) were present for the pre-eclampsia outcome. SGA status could not be determined when birthweight, gestational age at birth or infant sex was unavailable. Consequently, SGA status could not be classified for 13.7% of participants. The primary analysis was restricted to participants with complete data for all outcome components. It was chosen not to impute SGA as it is a derived outcome, and reliable imputation would have required an adequately specified imputation model for the underlying variables used to determine the sex- and gestational-age-specific birthweight centile. Baseline characteristics of participants with and without classifiable SGA status were compared to assess differences between the outcome-eligible and excluded samples (S1 Table).

### Potential covariates and confounders

Maternal and household factors, including maternal age at enrolment, parity, underweight status (defined as an average mid-upper arm circumference (MUAC) <23 cm), rural household location, education level, percentage likelihood of living below the poverty line, season at conception and country, were selected *a priori* for inclusion in the multivariable models as potential covariates and confounders [7,33,37–43].

Missing covariate data were low (<5%) and were imputed using multiple imputation by chained equations implemented in the mice package [44]. Missing covariate and WASH exposure data were handled under a missing-at-random assumption conditional on the variables included in the imputation models. Twenty imputed datasets were generated, with ten iterations per dataset. Participant identifiers and pregnancy outcomes, including pre-eclampsia, SGA, stillbirth and the composite outcome, were not imputed and were not used to predict missing covariate or exposure values.

### Statistical analysis

All statistical analyses were conducted in RStudio (R Foundation for Statistical Computing) [45].

Descriptive statistics were based on the full cohort, whereas regression analyses were restricted to participants with complete outcome data, excluding those with missing SGA. Bivariable and multivariable modified Poisson regression models with robust standard errors were used to assess associations between WASH exposures and any selected placenta-related complication.

Given the limited prior evidence concerning WASH-specific determinants of placenta-related complications across diverse country settings, the analysis was exploratory and hypothesis-generating. Data-driven selection was used to identify candidate WASH associations for future confirmatory research rather than to test prespecified causal hypotheses. In contrast, potential confounders were selected a priori and retained in all adjusted models regardless of statistical significance.

Candidate WASH exposure variables were identified from bivariable models using a screening threshold of p < 0.20. This inclusive threshold was chosen to reduce the risk of prematurely excluding potentially relevant WASH exposures [46]. As a sensitivity analysis, the variable-selection procedure was repeated without the initial bivariable p < 0.20 screening threshold, allowing all eligible WASH exposures to be considered.

Pearson’s correlation coefficients were generated for all combinations of selected variables with a p < 0.20 to identify strongly correlated and conceptually overlapping measures. Where ∣r∣ > 0.50, one exposure was selected by considering model fit, assessed using the akaike information criterion (AIC), alongside epidemiological relevance and interpretability.

A stepwise model selection approach was used to identify a parsimonious, best-fitting multivariable model [47]. All a priori confounders were retained in every model regardless of statistical significance*.* Models included country as a fixed effect. Variables were added in order of best model fit (AIC). An added variable was discarded where it did not improve the model fit (AIC), and the next variable was then added. This process continued until all variables had been trialled. The final multivariable model was checked for multicollinearity, with variables exceeding a variance inflation factor of 5 removed. Modified Poisson models were fitted separately within each imputed dataset. Regression coefficients and sandwich-robust within-imputation variances were then combined using Rubin’s rules [48]. Analyses used the stats, sandwich, MASS and car packages [45,49–51].

### Sub-group analysis

Potential effect modification by country was formally evaluated by adding interaction terms between country and each retained WASH exposure to the fully adjusted models. Overall interaction p-values were obtained using joint Wald tests of the relevant country-by-exposure interaction terms. Interaction models were fitted separately within each of the 20 imputed datasets, and coefficients and sandwich-robust within-imputation covariance matrices were combined using Rubin’s rules [48]. Country-specific analyses were conducted to describe the direction and magnitude of associations within each country.

Individual outcome analyses were also conducted by replacing the composite outcome with each component outcome: pre-eclampsia, SGA and stillbirth.

### Propensity-score-matched sensitivity analysis

As a sensitivity analysis, we assessed associations between household WASH exposures and pregnancy outcomes using propensity-score matching. Two binary exposures were assessed separately: piped water into the dwelling/plot versus other improved and unimproved sources, and improved versus unimproved sanitation. Propensity scores were estimated using logistic regression, with age at enrolment, parity and average MUAC represented categorically (see Table 1 for categorisations), along with rural residence, education, household poverty-likelihood quintile and season at conception. In the pooled cohort, we performed 1:1 nearest-neighbour matching without replacement, estimating the average treatment effect on the treated (ATT). Matching was conducted within country using exact matching on country, with a calliper of 0.2 standard deviations of the logit of the propensity score. Observations outside the region of common support were discarded. Covariate balance was assessed using absolute standardised mean differences before and after matching, with values below 0.10 considered indicative of acceptable balance. Household poverty likelihood was represented using quintiles in the propensity-score models to improve balance across its distribution using the MatchIt package [52]. Relative risks for each exposure–outcome pair were estimated in the matched sample using modified Poisson regression with robust standard errors, clustering by matched subclass and incorporating matching weights. Analyses were repeated for the selected placenta-related complication outcome, individual outcomes, and within each country.

**Table 1:** Characteristics and prevalence of selected placenta-related complications in the PRECISE cohort (The Gambia, Kenya and Mozambique).

Characteristic	PRECISE, N = 5,745
**Country**	
The Gambia	1,178 (20.5%)
Kenya	2,706 (47.1%)
Mozambique	1,861 (32.4%)
**Age at enrolment, median (IQR)**	26.0 (21.0–31.0)
Unknown	6
**Age at enrolment (years)**	
15–19	817 (14.2%)
20–24	1,683 (29.3%)
25–29	1,540 (26.8%)
30–34	1,018 (17.7%)
35–49	681 (11.9%)
Unknown	6
**Gestational age at enrolment, median (IQR), weeks**	20.9 (16.1–25.4)
Unknown	111
**Duration of follow-up from enrolment to delivery, median (IQR), weeks**	18.0 (13.0–22.9)
Unknown	43
**Parity**	
0	1,695 (29.5%)
1	1,370 (23.8%)
2-4	2,129 (37.1%)
5+	551 (9.6%)
**Maternal BMI, median (IQR)**	23.8 (21.3–27.1)
Unknown	26
**Maternal BMI categorised**	
Underweight (<18.5)	293 (5.1%)
Normal (18.5–24.9)	3,189 (55.8%)
Overweight (25–29.9)	1,517 (26.5%)
Obese (>30)	720 (12.6%)
Unknown	26
**MUAC, median (IQR)**	26.8 (24.5–29.5)
Unknown	22
**Average MUAC categorised (cm)**	
Underweight (<23)	549 (9.7%)
Normal (23–26.4)	2,024 (35.9%)
Overweight (26.5–29.9)	1,773 (31.4%)
Obese (>30)	1,296 (23.0%)
Unknown	103
**Household location**	
Rural	1,684 (30.1%)
Peri-urban	879 (15.7%)
Urban	3,030 (54.2%)
Unknown	152
**Highest education level**	
None	1,084 (18.9%)
Primary	2,254 (39.3%)
Secondary	2,012 (35.1%)
Higher	388 (6.8%)
Unknown	7
**% likelihood below the poverty line, median (IQR)**	21.4 (6.1–42.3)
Unknown	8
**Pre-eclampsia**	454 (7.9%)
Unknown	7
**Size for gestational age**	
Very Small for Gestational Age (<3rd centile)	381 (7.7%)
Small for Gestational Age (<10th centile)	574 (11.6%)
Appropriate for Gestational Age (10th to 90th centile)	3,488 (70.4%)
Large for Gestational Age (>90th centile)	341 (6.9%)
Very Large for Gestational Age (>97th centile)	173 (3.5%)
Unknown	788
**Stillbirth**	179 (3.1%)
**Selected placenta-related complications: Pre-eclampsia, small-for-gestational-age (SGA < 10th percentile) or stillbirth**	1,335 (26.9%)
Unknown	788

Abbreviations: BMI, body mass index; IQR, interquartile range; MUAC, mid-upper arm circumference; PRECISE, PREgnancy Care Integrating translational Science, Everywhere.

## Results

### Characteristics of study participants

A total of 5,745 participants were included in this study, 1,178 from The Gambia, 2,706 from Kenya and 1,861 from Mozambique (Table 1). The median age of participants was 26 years (IQR: 21–31). Parity was most commonly between 2 and 4 births in The Gambia (41%) and Kenya (38%), but in Mozambique, nulliparity (0) was more frequent (39%) (S2 Table). The median BMI, measured at the booking appointment, was 23.8 (IQR: 21.3–27.1) and the median MUAC was 26.8 cm (IQR: 24.5–29.5) (Table 1). Overall, 10% of women were underweight (MUAC < 23 cm), with the highest prevalence in Kenya (14%) and the lowest in Mozambique (3%) (S2 Table).

The Gambia had the highest proportion of women without formal education (64%), compared with Kenya (9%) and Mozambique (6%). In terms of residence, 60% of Kenyan women lived in urban areas and 32% in peri-urban areas. In contrast, 37% of women in The Gambia resided in urban areas and 61% in rural areas. In Mozambique, 56% of women lived in urban areas, while 42% lived in rural areas (S2 Table).

### Prevalence of selected placenta-related complications

Overall, 8% of women had pregnancies complicated by pre-eclampsia, 3% experienced a stillbirth, and 19% had an SGA baby (<10^th^ percentile), with 27% having at least one of these selected placenta-related complications (Table 1 and Fig 4). Overall, selected placenta-related complications were highest in The Gambia (34%), followed by Kenya (27%) and Mozambique (23%) (S2 Table and Fig 4).

**Fig 4:**
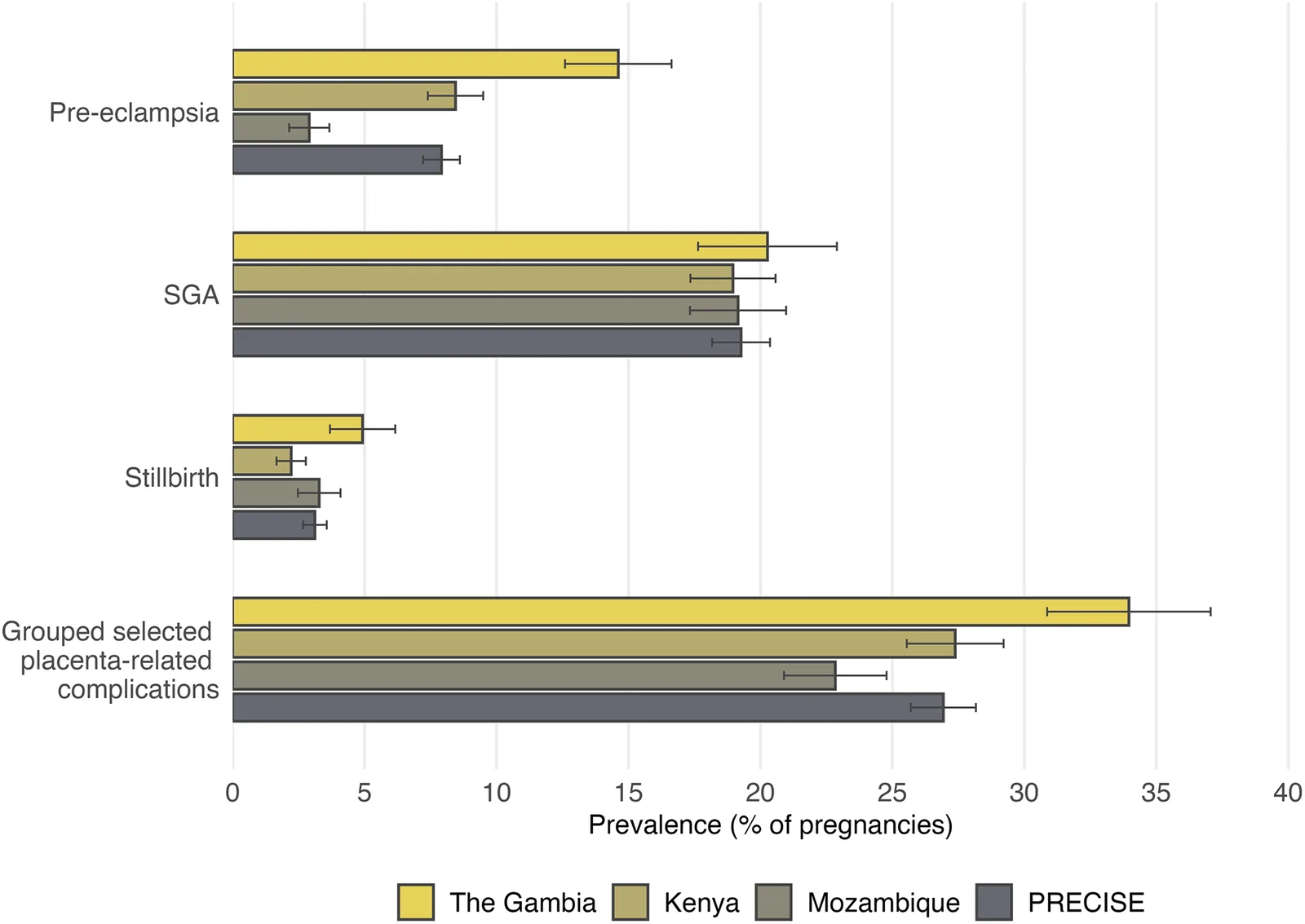
Prevalence of placenta‑related outcomes by country and the overall PRECISE cohort. Horizontal grouped bars show the proportion of pregnancies with pre‑eclampsia, SGA, stillbirth, and the composite selected placenta‑related complication outcome (see Methods). Error bars represent 95% confidence intervals (Wald method). Abbreviations: PRECISE, PREgnancy Care Integrating translational Science, Everywhere; SGA, small-for-gestational-age.

### Water, sanitation and hygiene

Across the PRECISE cohort, piped water into the dwelling/plot was the most common source of drinking water (50%) (Table 2). This was highest in Mozambique (71%), compared with Kenya (45%) and The Gambia (29%) (S3 Table). Few households (3%) used an alternative drinking or non-drinking water source. The median time to collect water, if it was collected outside the household, was 5 minutes (IQR: 2–10) (Table 2).

**Table 2:** Water, sanitation and hygiene facilities and practices in the households of pregnant women in the PRECISE cohort (The Gambia, Kenya and Mozambique).

Variable	PRECISE, N = 5,745
**Source of drinking water**	
Piped water into dwelling/plot	2,865 (50.1%)
Other improved water source (piped water into neighbour’s dwelling or public tap, boreholes or tubewells, protected dug wells, protected springs, rainwater, and packaged or delivered water)	2,535 (44.3%)
Unimproved water source (surface water, unprotected well or spring)	321 (5.6%)
Unknown	24
**Source of non-drinking water (used for cooking/washing/personal hygiene)**	
Piped water into dwelling/plot	2,886 (50.4%)
Other improved water source (piped water into neighbour’s dwelling or public tap, boreholes or tubewells, protected dug wells, protected springs, rainwater, and packaged or delivered water)	2,479 (43.3%)
Unimproved water source (surface water, unprotected well or spring)	361 (6.3%)
Unknown	19
**Alternative drinking and non-drinking water source**	195 (3.4%)
Unknown	25
**Location of water source**	
In own dwelling	760 (13.3%)
In own yard or plot	2,464 (43.0%)
Elsewhere	2,511 (43.8%)
Unknown	10
**Time to collect water if collected outside the household (minutes), median (IQR)**	5.0 (2.0, 10.0)
**Sanitation facility**	
Improved (flush toilets connected to piped sewer systems, septic tanks or pit latrines; pit latrines with slabs, composting toilets)	4,568 (79.9%)
Unimproved (pit latrines without a slab or platform, hanging latrines, bucket latrines or open defecation)	1,150 (20.1%)
Unknown	27
**Sanitation facility location**	
In own dwelling	1,186 (20.7%)
In own yard or plot	4,324 (75.4%)
Elsewhere	224 (3.9%)
Unknown	11
**Share sanitation facility with non-household members**	2,071 (36.1%)
Unknown	15
**Handwashing facility**	
Fixed facility (sink/tap) in dwelling	462 (8.1%)
Fixed facility (sink/tap) in yard/plot	317 (5.6%)
Mobile object (bucket/jug/kettle)	4,449 (78.2%)
No handwashing place in dwelling or yard/plot	461 (8.1%)
Unknown	56

For sanitation, households predominantly had improved sanitation facilities (e.g., flush toilets connected to piped sewer systems, septic tanks or pit latrines; pit latrines with slabs, composting toilets) (80%) compared with unimproved facilities (pit latrines without a slab or platform, hanging latrines, bucket latrines or open defecation) (20%) (Table 2). Use of unimproved facilities was highest in The Gambia (38%) compared with Mozambique (20%) and Kenya (13%). Across all countries, 76% of households had a sanitation facility located in the yard or plot (Table 2). Overall, 36% of households shared their sanitation facility, but this differed markedly between countries, with 55% in Kenya, 36% in The Gambia and 9% in Mozambique (S3 Table).

Most households in all countries used a mobile object (bucket/jug/kettle) for handwashing (78%). A higher proportion of households in Kenya had no handwashing facility located in the dwelling or yard/plot (15%) compared with The Gambia (6%) and Mozambique (0.2%) (S3 Table).

### Joint Monitoring Programme (JMP) ranked categories and national average

In all three countries, the proportion of households with access to at least basic water services exceeded the national average according to the JMP. In The Gambia, 95% of rural households and 96% of urban households in the PRECISE cohort had access to basic water services. In Kenya, the figures were 95% for rural and urban households, while in Mozambique, 83% of rural households and 93% of urban households had access to basic water services (S4 Table).

Access to basic sanitation facilities varied across the three countries compared with national averages. In The Gambia, 44% of rural households had access to basic sanitation, compared with the national average (24%) [31]. However, in urban households, 44% of households had access to basic sanitation compared with the national average (61%). Rural (35%) and urban (40%) households in Kenya had similar access to basic sanitation facilities compared to the rural (35%) and urban (40%) national average [31]. In Mozambique, 74% of rural households had access to basic sanitation, compared with the national average (23%) [31]. In urban households, 73% of households had access to basic sanitation compared with the national average (61%) [31] (S4 Table). The survey question did not specify whether soap and water were available in the household, so it was not possible to categorise the data into basic hygiene levels.

### Association between WASH and selected placenta-related complications in women

Associations between WASH exposures and placenta-related complications were then assessed using bivariable and multivariable regression models using complete outcome data, excluding those with missing SGA (Table 3 and S1 Table). Missing covariate data were low and therefore imputed. Most WASH variables showed some evidence of an association with the selected placenta-related complication outcome (p < 0.20), except for alternative water sources for drinking and non-drinking water and for sanitation facility location (Table 3). Drinking water source, non-drinking water source, and source location were correlated (r^2^ > 0.5), with drinking water source retained in the analysis due to it having the lowest AIC (S2 Fig). Drinking water was also deemed to have a clearer epidemiological interpretation in relation to exposure to waterborne pathogens. The variables capture related but not identical dimensions of household water access (Cramer’s V = 0.92).

**Table 3:** Bivariable models (modified Poisson regression with robust standard errors) of eight WASH variables and their associations with selected placenta‑related complications in the PRECISE cohort in The Gambia, Kenya and Mozambique (Imputed dataset).

Variable	Overall, N = 4,957	No selected placenta-related complication, N = 3,622	Selected placenta-related complications, N = 1,335	RR	95% CI	p-value
**Source of drinking water**						
Piped water into dwelling/plot	2,542 (51.4%)	1,942 (53.7%)	600 (45.1%)	Ref		
Other improved water source	2,122 (42.9%)	1,472 (40.7%)	650 (48.9%)	1.30	1.18–1.43	<0.001
Unimproved water source	280 (5.7%)	200 (5.5%)	80 (6.0%)	1.21	0.99–1.48	0.057
Unknown	13	8	5			
**Source of non-drinking water**						
Piped water into dwelling/plot	2,554 (51.6%)	1,959 (54.1%)	595 (44.7%)	Ref		
Other improved water source	2,084 (42.1%)	1,442 (39.9%)	642 (48.3%)	1.32	1.20–1.46	<0.001
Unimproved water source	310 (6.3%)	217 (6.0%)	93 (7.0%)	1.29	1.08–1.56	0.006
Unknown	9	4	5			
**Alternative water source for uses other than drinking**	166 (3.4%)	121 (3.3%)	45 (3.4%)	1.01	0.78–1.30	0.950
Unknown	13	8	5			
**Location of water source**						
In own dwelling	654 (13.2%)	499 (13.8%)	155 (11.6%)	Ref		
In own yard or plot	2,201 (44.4%)	1,671 (46.1%)	530 (39.8%)	1.01	0.87–1.18	0.869
Elsewhere	2,098 (42.4%)	1,451 (40.1%)	647 (48.6%)	1.30	1.12–1.51	<0.001
Unknown	4	1	3			
**Unimproved sanitation facility - Yes**	958 (19.4%)	658 (18.2%)	300 (22.6%)	1.22	1.09–1.35	<0.001
Unknown	18	9	9			
**Sanitation facility location**						
In own dwelling	1,004 (20.3%)	749 (20.7%)	255 (19.1%)	Ref		
In own yard or plot	3,770 (76.1%)	2,743 (75.8%)	1,027 (77.1%)	1.07	0.95–1.21	0.245
Elsewhere	178 (3.6%)	128 (3.5%)	50 (3.8%)	1.11	0.86–1.44	0.427
Unknown	5	2	3			
**Share sanitation facility with non-household members - Yes**	1,737 (35.1%)	1,230 (34.0%)	507 (38.1%)	1.14	1.04–1.25	0.007
Unknown	9	5	4			
**Handwashing facility**						
Fixed facility (sink/tap) in dwelling	400 (8.1%)	309 (8.6%)	91 (6.9%)	Ref		
Fixed facility (sink/tap) in yard/plot	267 (5.4%)	189 (5.3%)	78 (5.9%)	1.28	0.98–1.65	0.066
Mobile object (bucket/jug/kettle)	3,879 (78.8%)	2,841 (78.9%)	1,038 (78.6%)	1.17	0.97–1.41	0.098
No handwashing place in dwelling or yard/plot	374 (7.6%)	260 (7.2%)	114 (8.6%)	1.33	1.05–1.68	0.018
Unknown	37	23	14			

Abbreviations: CI, confidence interval; RR, relative risk; PRECISE, PREgnancy Care Integrating translational Science, Everywhere; Ref, Reference group.

Descriptive counts and unknown values are based on the observed dataset, whereas RRs, confidence intervals and p-values are from the imputed analyses. Comparisons of the imputed and non-imputed bivariable analyses are presented in S5 Table.

When the variable-selection procedure was repeated without the initial bivariable p < 0.20 screening threshold, the same drinking-water source and sanitation exposures were retained, indicating that the final exposure set was not dependent on the screening step.

Two WASH variables were retained in the multivariable model after adjusting for confounders and covariates (Table 4). In the imputed analysis, other improved water sources (adjusted relative risk (aRR) 1.18, 95% confidence interval (CI) 1.07–1.31, p = 0.002), unimproved water sources (aRR 1.23, 95% CI 1.00–1.50, p = 0.049), and unimproved sanitation facilities (aRR 1.15, 95% CI 1.02–1.30, p = 0.019) were associated with a higher risk of any selected placenta-related complication. The full multivariable model, including all a priori covariates and confounders, is available in S7 Table. In sensitivity analyses, associations were modestly stronger when non-drinking-water source was used instead of drinking-water source, but the direction of association was consistent across both specifications (S8 Table).

**Table 4:** Final selected multivariable modified Poisson regression of retained WASH variables and their associations with selected placenta‑related complications in the PRECISE cohort in The Gambia, Kenya and Mozambique.

	Unadjusted analyses			Adjusted analyses^		
Variable	RR	95% CI	p-value	aRR	95% CI	p-value
**Source of drinking water**						
Piped water into dwelling/plot	Ref			Ref		
Other improved water source	1.28	1.16–1.40	<0.001	1.18	1.07–1.31	0.002
Unimproved water source	1.17	0.96–1.43	0.119	1.23	1.00–1.50	0.049
**Unimproved sanitation facility**	1.17	1.05–1.30	0.005	1.15	1.02–1.30	0.019

RR, relative risk; CI, confidence interval; PRECISE, PREgnancy Care Integrating translational Science, Everywhere; Ref, Reference group.

^Adjusted for: age at enrolment, parity, average MUAC <23 cm (Underweight), household located in rural area, education, % likelihood below the poverty line, season at conception and country.

In the non-imputed analysis, the estimate for other improved water sources was similar (aRR 1.19, 95% CI 1.07–1.33, p = 0.002). Estimates for unimproved water sources (aRR 1.09, 95% CI 0.87–1.37, p = 0.450) and unimproved sanitation facilities (aRR 1.11, 95% CI 0.98–1.26, p = 0.107) were attenuated, although they remained directionally consistent with the imputed estimates (S6 Table).

Propensity-score-matched analyses supported associations between the composite outcome and both non-piped water sources (RR 1.27, 95% CI 1.13–1.43, p < 0.001) and unimproved sanitation (RR 1.16, 95% CI 1.00–1.35, p = 0.049). Matching achieved acceptable balance in both analyses, with maximum absolute standardised mean differences decreasing from 0.285 to 0.057 for water source and from 0.293 to 0.031 for sanitation (S11 Table).

### Disaggregated results by country

Formal interaction tests provided no clear evidence that associations with the composite outcome differed by country for either drinking-water source (χ²[4]=6.18; p for interaction = 0.186) or unimproved sanitation (χ²[2]=3.37; p for interaction = 0.186). Country-specific estimates are therefore presented as exploratory analyses describing the direction and magnitude of associations within each setting (S9 Table). Compared with water piped into the dwelling or plot, other improved water sources were associated with a higher risk of any selected placenta-related complication outcome in The Gambia (aRR 1.31, 95% CI 1.01–1.70, p = 0.042) and Kenya (aRR 1.22, 95% CI 1.05–1.42, p = 0.008), but not in Mozambique (aRR 1.07, 95% CI 0.87–1.33, p = 0.511). Unimproved water sources were associated with a higher risk of any selected placenta-related complication in Kenya (aRR 1.66, 95% CI 1.20–2.29, p = 0.002), but no clear associations were identified in The Gambia or Mozambique. Unimproved sanitation was associated with a higher risk of any selected placenta-related complication in Mozambique (aRR 1.28, 95% CI 1.04–1.57, p = 0.019), but not in The Gambia or Kenya (S9 Table).

Propensity-score-matched analyses supported the association between non-piped water sources and the composite outcome in Kenya (RR 1.21, 95% CI 1.01–1.44, p = 0.036) and between unimproved sanitation and the composite outcome in Mozambique (RR 1.45, 95% CI 1.09–1.94, p = 0.012). No clear matched associations were identified in The Gambia or for the other exposure-country combinations. Matching achieved acceptable covariate balance in all country-specific analyses, with maximum post-matching absolute standardised mean differences ranging from 0.022 to 0.088 (S12 Table).

### Disaggregated results by placenta-related complications

For individual complications, other improved water sources were associated with an increased risk of SGA (aRR 1.15, 95% CI 1.01–1.32, p = 0.030). Unimproved water sources were associated with pre-eclampsia (aRR 1.71, 95% CI 1.14–2.57, p = 0.010), while unimproved sanitation was associated with SGA (aRR 1.21, 95% CI 1.05–1.40, p = 0.010). No statistically significant associations were identified with stillbirth (S10 Table).

Propensity-score-matched analyses supported the association between non-piped water sources and SGA (RR 1.26, 95% CI 1.09–1.46, p = 0.002). No clear matched associations were identified between non-piped water sources and pre-eclampsia or stillbirth, or between unimproved sanitation and any individual complication. Matching achieved acceptable covariate balance across all analyses, with maximum post-matching absolute standardised mean differences ranging from 0.031 to 0.058 (S13 Table).

## Discussion

This secondary analysis of the PRECISE cohort assessed household and community WASH conditions among pregnant women and the associations with selected placenta-related complications in The Gambia, Kenya and Mozambique. Although studies have explored some of these associations, the associations between WASH conditions and a composite of placenta-related complications, including pre-eclampsia, SGA and stillbirth, have not been well studied. After adjustment, women using other improved water sources had an 18% higher risk of the composite outcome, while women using unimproved water sources had a 23% higher risk. Unimproved sanitation was associated with a 15% higher risk. Outcome-specific analyses indicated that other improved water sources and unimproved sanitation were associated with SGA, while unimproved water sources were associated with pre-eclampsia. No statistically significant associations were identified with stillbirth individually. These findings suggest that the composite association was principally reflected in associations with SGA and pre-eclampsia and should not be interpreted as evidence of equivalent associations with all three outcomes separately. The absence of a statistically significant association with stillbirth should be interpreted cautiously because the smaller analytic sample and low event count limited statistical power to detect an association.

These findings are broadly consistent with previous studies examining household water sources and adverse pregnancy complications. In Sierra Leone, using a well or borehole as the main source of drinking water was linked to higher odds of pre-eclampsia/eclampsia [53]. In India, groundwater, compared with piped water, was associated with LBW and very LBW [54]. Similarly, a pooled analysis in the African Great Lakes region (covering Burundi, the Democratic Republic of Congo, Kenya, Rwanda, Tanzania, and Uganda) found that drinking water from an unimproved source away from the dwelling was associated with stillbirth [55]. However, in Nepal [56], only weak evidence of associations between drinking water sources and stillbirth was found.

Use of unimproved sanitation facilities, including open defecation, was associated with a 15% higher risk of any selected placenta-related complication, compared with improved sanitation facilities. These findings are similar to other studies. In India, a study found unimproved sanitation was associated with LBW and VLBW [54]. In Nepal, studies have found associations with unimproved sanitation and open defecation with perinatal mortality and stillbirth [56,57]. In rural India, Padhi et al. [58] identified self-reported open defecation to be associated, after adjustment, with increased odds of adverse pregnancy outcomes, defined as an event of preterm birth, LBW, spontaneous abortion or stillbirth. Our study could not explore the associations between open defecation and selected placenta-related complications due to the low number of participants who reported practising open defecation.

Water sources not piped into the participant’s dwelling or plot, including many other improved and unimproved sources, and unimproved sanitation facilities may increase exposure to pathways relevant to placenta-related complications in pregnant women (Fig 2). Water sources outside the household have a higher likelihood of contamination, including inorganic contaminants or infectious pathogens that pose risks during pregnancy. Water collected from sources outside the household has to be carried and stored within the household, which can introduce contamination, while unimproved sources do not adequately protect against external contamination. Contaminated water can be ingested by pregnant women or contaminate food, soil, hands, fomites, or flies [4] (Fig 2). Unimproved sanitation facilities do not hygienically separate human faeces from contact, resulting in unsafe containment that can lead to the transmission of pathogens from one person to another. Acute maternal enteric infections, including *Listeria monocytogenes,* cholera *and Escherichia coli*, can be introduced through both these pathways during pregnancy and may contribute to various adverse outcomes, including pre-eclampsia, SGA and stillbirth [5–9,11,59,60] (Fig 2). In China, preterm birth, fetal distress and LBW were significantly higher in pregnant women infected with water-borne Hepatitis E virus than those who were not [61].

Collecting water from sources located away from the household may pose health risks to pregnant women due to the increased physical burden (Fig 2). Women are often primarily responsible for water collection [62], and given the observed association with water sources located outside the household or premises, it is likely that the study participants had to travel to access water. However, this study did not determine whether the women themselves were collecting the water or how frequently. A systematic review of the effects of occupational activities during pregnancy on outcomes highlights the limited research currently available, particularly concerning lifting weights and placenta-related complications [25]. Only one study investigated stillbirth and heavy lifting, but found no association [63]. In addition, the review found a link between lifting weights (≥11 kg) and increased odds of pre-eclampsia, although there was no association between prolonged standing or walking and pre-eclampsia [25]. Meta-analysis indicated no association between lifting weights (≥11 kg) and SGA infants, although prolonged standing and walking were associated with increased odds of SGA, despite low certainty [25]. The pathways through which heavy lifting could lead to placenta-related complications are not well understood. Prolonged standing is linked to decreased cardiac output and plasma volume, which may also impact fetal growth [64]. Collecting water may increase exposure to higher ambient temperatures, which can lead to heat strain, dehydration and haemodynamic stress, consistent with epidemiological links between heat exposure and low birthweight and stillbirth [23,24,27–29] (Fig 2).

Country-specific estimates varied across the three settings. Other improved water sources were associated with selected placenta-related complications in The Gambia and Kenya, while unimproved water sources were associated with the outcome in Kenya. Unimproved sanitation was associated with the outcome in Mozambique. However, formal interaction tests provided no clear evidence that associations with either drinking-water source or unimproved sanitation differed by country. The country-specific findings should therefore be interpreted as exploratory. Country-specific differences may stem from a variety of factors, including variations in community-level WASH access, affordability, availability, accessibility, water quality, and local behavioural practices related to water usage and sanitation. For example, even “improved” water sources may still be subject to contamination or inconsistent quality [3,65]. Additionally, cultural practices around water storage and sanitation could influence exposure to pathogens, thereby affecting maternal health outcomes differentially across regions. These differences may also reflect variation in exposure prevalence and event counts across countries.

### Limitations

Although WASH exposures were measured before the outcome, causal interpretation remains limited due to residual confounding, the observational design, and the use of proxy household WASH indicators. As the cohort was not restricted to uncomplicated pregnancies at enrolment, residual confounding by baseline maternal or pregnancy risk may remain. WASH conditions were self-reported and measured only once at enrolment, which may have introduced reporting bias and exposure misclassification if household conditions changed during pregnancy. Women were also enrolled at varying gestational ages (median 20.9 weeks; IQR 16.1–25.4), resulting in different intervals between WASH assessment and delivery. Measurements obtained later in pregnancy may not reflect conditions earlier in pregnancy, including during placentation. The study sites were not designed to be nationally representative of the participating countries.

The composite outcome combined complications with distinct pathophysiology and frequencies and should not be interpreted as evidence of equivalent associations with pre-eclampsia, SGA and stillbirth. Outcome-specific findings were principally reflected in associations with SGA and pre-eclampsia, while no statistically significant associations were identified with stillbirth. However, the individual-outcome and country-specific analyses had smaller samples and, in some cases, low event counts. Their null findings may therefore reflect limited statistical power rather than the absence of an association.

SGA status could not be classified for 13.7% of participants because information required to determine the sex- and gestational-age-specific birthweight centile was unavailable. Participants with and without classifiable SGA status differed on several measured characteristics (S1 Table). Restricting the primary analysis to participants with complete outcome classification may therefore have introduced selection bias if SGA-data availability was related to WASH exposure or pregnancy outcome. Consequently, the findings may not fully represent the entire enrolled cohort.

The selection of WASH exposures involved bivariable screening followed by AIC-based stepwise selection. Although the same exposure set was retained when the procedure was repeated without the initial screening threshold, data-driven selection can produce sample-dependent model specifications, overstate the importance of retained variables, and fail to capture uncertainty introduced by the selection process. The selected exposure model should therefore be regarded as exploratory.

No prospective sample-size calculation was undertaken specifically for this secondary WASH analysis because the available sample was determined by the wider PRECISE cohort. The post hoc power assessment was intended only to contextualise the available sample and should not be interpreted as justification for the study design or validation of the observed results. It used simplified two-sample comparisons of proportions and did not reproduce the multivariable modified Poisson models, including covariate adjustment, country effects, or unequal exposure distributions. Post hoc power estimates also have recognised limitations, including circularity when calculated using observed effect sizes [66].

The questionnaire offered a restricted set of WASH measures. Additional information on water storage, water quantity, menstrual health practices, and handwashing materials could have provided better insights into when and how risks are introduced. Additionally, structured observations may offer a more nuanced understanding of WASH-related behaviours and their direct impact on women, beyond the household level. Some variables in the study did not accurately reflect household practices. For instance, questions about water collection did not specify whether the mother or another household member collected the water. Similarly, hygiene questions did not clarify whether soap and water were used for handwashing and if performed correctly.

The study also could not identify the specific pathway through which contamination may have reached pregnant women. Environmental testing of water, food, soil, hand swabs, fomites, or flies for faecal indicators or specific pathogens, such as *E. coli* and total coliforms, could have improved our understanding of contamination pathways and helped inform more targeted interventions. Multi-pathogen PCR analyses or other techniques could have been employed to identify individual pathogens, enhancing our understanding of contamination pathways and infection risks for pregnant women.

## Conclusions

A quarter of women enrolled in this study had one or more of the following selected placenta-related complications: pre-eclampsia, stillbirth, or an SGA infant. After adjustment, other improved and unimproved water sources were associated with 18% and 23% higher risks of the composite outcome, respectively, compared with water piped into the dwelling or plot. Unimproved sanitation was associated with a 15% higher risk of the composite outcome. Outcome-specific analyses identified associations with SGA and pre-eclampsia, while no statistically significant association was identified with stillbirth individually. Overall, our findings are consistent with other studies, adding to the evidence on associations between WASH conditions and selected placenta-related complications.

These findings underscore the importance of integrating WASH interventions into health improvement programmes globally. Improving access to safe water and sanitation could plausibly contribute to better pregnancy outcomes, but intervention studies are required to determine whether household WASH improvements reduce placenta-related complications. Interventions could consider improving water quality or reducing the physical burden associated with water collection. However, they should be developed with the local context and practices in mind, as WASH behaviours and exposures often differ greatly between populations. Further robust research is needed to confirm the associations identified in this study and to co-design context-appropriate improvements to all aspects of WASH services in collaboration with communities.

## Supporting information

S1 Table Baseline characteristics of participants with and without size‑for‑gestational‑age data in the PRECISE cohort in The Gambia, Kenya and Mozambique. (DOCX)

S2 Table Characteristics and prevalence of selected placenta-related complications in the PRECISE cohort in The Gambia, Kenya and Mozambique. (DOCX)

S3 Table Water, sanitation and hygiene facilities and practices in the households of pregnant women in the PRECISE cohort in The Gambia, Kenya and Mozambique. (DOCX)

S4 Table Comparison of Joint Monitoring Programme 2022 national WASH data and the PRECISE cohort in The Gambia, Kenya and Mozambique. (DOCX)

S5 Table Bivariable models (modified Poisson regression with robust standard errors) of eight WASH variables and their associations with selected placenta‑related complications in the PRECISE cohort in The Gambia, Kenya and Mozambique with imputed and non-imputed datasets. (DOCX)

S6 Table Final selected multivariable modified Poisson regression of retained WASH variables and their associations with selected placenta‑related complications in the PRECISE cohort in The Gambia, Kenya and Mozambique, with imputed and non-imputed datasets. (DOCX)

S7 Table Full multivariable modified Poisson regression model estimating associations between household WASH exposures, covariates, and selected placenta‑related complications in the PRECISE cohort. (DOCX)

S8 Table Sensitivity analysis comparing drinking-water and non-drinking-water exposures and their associations with selected placenta‑related complications in the PRECISE cohort. (DOCX)

S9 Table Comparison of the adjusted final multivariable model for the PRECISE cohort by country (The Gambia, Kenya and Mozambique). (DOCX)

S10 Table Comparison of the adjusted final multivariable model for the PRECISE cohort by pregnancy complication. (DOCX)

S11 Table Propensity‑score–matched modified Poisson regression models (with robust standard errors) estimating the association between household WASH exposures and the selected placenta‑related complication outcome in the PRECISE cohort (The Gambia, Kenya and Mozambique). (DOCX)

S12 Table Propensity‑score–matched modified Poisson regression models (with robust standard errors) estimating associations between household WASH exposures and the selected placenta‑related complication outcome within each country in the PRECISE cohort (The Gambia, Kenya and Mozambique). (DOCX)

S13 Table Propensity‑score–matched modified Poisson regression models (with robust standard errors) estimating associations between household WASH exposures and individual placenta‑related complications in the PRECISE cohort (The Gambia, Kenya and Mozambique). (DOCX)

S1 Fig Power to detect associations between WASH exposures and any selected placenta‑related complications (pre-eclampsia, small-for-gestational-age or stillbirth) in the PRECISE cohort. Statistical power to detect a given relative risk (RR) between WASH exposure and selected placenta‑related complications, assuming the analytic sample size of 4,957 women and an outcome prevalence of 27%. Power was calculated for RRs ranging from 1.1 to 1.5 using two‑sample comparisons of proportions, with exposure prevalences ranging from 10% to 50%. Lines represent different exposure prevalence scenarios.
(TIFF)

S2 Fig Correlation matrix of candidate WASH exposure variables for multivariable modelling. WASH variables with a p‑value <0.2 in the bivariable modified Poisson models were retained as candidates for the multivariable model. Pearson correlation coefficients were then generated for all pairwise combinations of these candidate variables to identify potential multicollinearity. The matrix presents the strength and direction of correlations, supporting decisions regarding variable inclusion in the final model.
(TIF)

S1 Data WHO/UNICEF JMP database for estimates of household drinking water, sanitation and hygiene in The Gambia, Kenya and Mozambique in 2022 [31]. (CSV)

S1 Checklist Inclusivity in global research. (DOCX)

S1 File The PRECISE network study protocol v2.0. (PDF)

S1 Acknowledgements Membership of the PRECISE and PRECISE-DYAD Networks. (DOCX)
