# Supplementary material for: Evaluating the association between household water, sanitation and hygiene (WASH) and selected placenta-related complications in The Gambia, Kenya and Mozambique
Source: PLOS Glob Public Health. 2026 Oct 15;6(10):e0005004. doi: 10.1371/journal.pgph.0005004 (PMC13671507; doi:10.1371/journal.pgph.0005004)
Supplement: Supplementary file 1 [file pgph.0005004.s018.pdf]

THE PRECISE NETWORK STUDY PROTOCOL

PRECISE
PREgnancy Care Integrating translational Science, Everywhere

Version 2.0

10th September 2020

Table of Contents

A. List of appendices 3

B. List of tables and figures 3

C. Preamble 4

D. PRECISE investigator team 4

E. Background & rationale 7

F. Objectives of PRECISE 8

G. Research settings 9

H. The women 11

I. Participant cohorts, data and sample collection 13

J. Creating the PRECISE biorepository (see Appendix 6 for Standard Operating Procedures) ... 20

K. Interactions with the clinical service 3

L. Sample size and general statistical approaches 3

M. The PRECISE database and data management 5

N. Ethics 12

O. Timeline 14

P. Research themes 14

A. List of appendices

Appendix 1. PRECISE Theory of Change
Appendix 2. PRECISE Research Capacity Building Framework
Appendix 3. PRECISE Advocacy Framework
Appendix 4. PRECISE Data collection forms: demographic, non-clinical and clinical data
Appendix 5. PRECISE Eligibility and Recruitment Flowchart
Appendix 6. PRECISE Biobank Standard Operating Procedures (SOPs)
Appendix 7. Interactions with the clinical service
Appendix 8. PRECISE Governance Framework
Appendix 9. PRECISE Information sheets and consent forms
Appendix 10. Health Geography approach
Appendix 11. Socio-cultural qualitative work
Appendix 12. Respectful maternity care: overview of evidence and research gaps
Appendix 13. TraCer protocol
Appendix 14. CRADLE user guide
Appendix 15. Catalyst project protocol (Mental Health)

B. List of tables and figures

Table 1. Co-investigator experience and institutions
Table 2. Community engagement
Table 3. Sampling from non-pregnant women of reproductive age
Table 4. Sampling from unselected pregnant women and women at time-of-disease (and their infants)
Table 5. Sample size calculations

Figure 1. The Geography of PRECISE
Figure 2. A holistic approach to pregnancy research
Figure 3. Gambian sites
Figure 4. Kenyan sites
Figure 5. Mozambican sites
Figure 6. Flow and sampling of nonpregnant women of reproductive age
Figure 7. Flow and sampling of unselected pregnant women
Figure 8. Flow and sampling of pregnant women with suspected/confirmed placental disease
Figure 9. Flow and sampling of women with stillbirth recruited postpartum
Figure 10. Probability of observing 600 outcomes
Figure 11. Data management plan for the biorepository
Figure 12. Overview Gantt chart for PRECISE activity
Figure 13. Hulton framework for assessing quality of care
Figure 14. Health systems framework

C. Preamble

The PRECISE Network is a new and broadly-based group of research scientists and health advocates mainly based in the UK and Africa. With core funding from the UK Research and Innovation (UKRI), we are establishing this network through a shared project investigating three important complications of pregnancy: high blood pressure (hypertension), babies who are smaller than they should be before birth (fetal growth restriction) and babies who die before birth (stillbirth). We estimate that about 46,000 women and two-and-a-half million babies (both before and after birth) die due to these problems every year, and half of them die in Africa [1]. In addition, about 50 million women and babies will have their short and long-term health altered because of these complications. These numbers represent one of the great global inequalities of our time.

D. PRECISE investigator team

The internationally renowned co-investigator network has been established to deliver scientific excellence across the PRECISE objectives and broad programme of holistic, interdisciplinary pregnancy research. Figure 1 and Table 1 shows the global geography of institutions collaborating in this network.

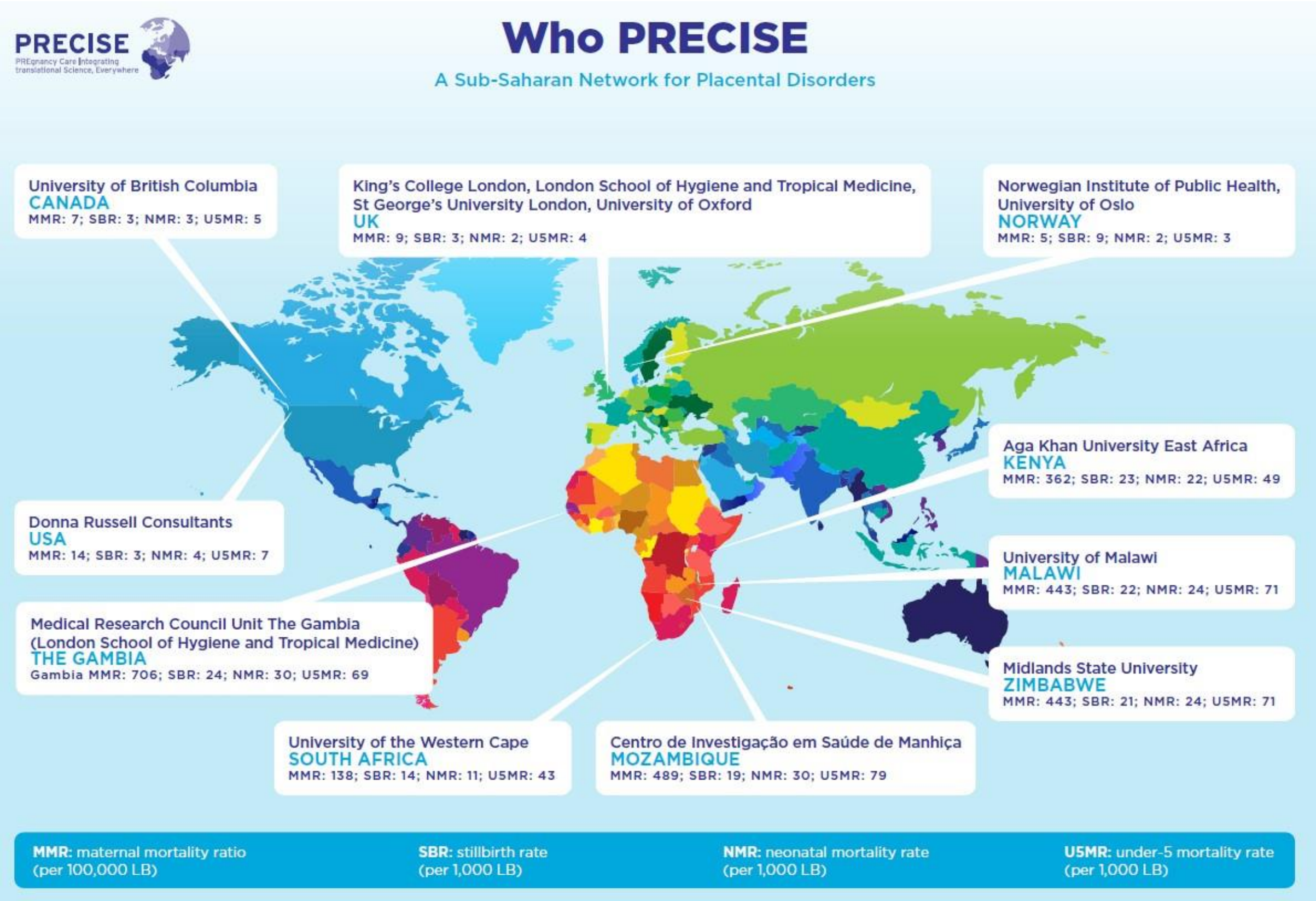

Figure 1 The geography of PRECISE
MMR, maternal mortality ratio; *NMR*, neonatal mortality rate; *SBR*, stillbirth rate; *U5MR*, under-5 mortality rate

Table 1 Co-Investigator expertise and institutions

Name	Job Title	Institution/ Organisation
**Marleen Temmerman**	Professor, Director of the Centre of Excellence in Women and Child Health	Aga Khan University, Nairobi
**Angela Koech Etyang**	Obstetrician Scientist	Aga Khan University, Nairobi
**Sikolia Wanyonyi**	Consultant Obstetrician/Gynaecologist and Fetal-Maternal Specialist	Aga Khan University, Nairobi
**Geoffrey Omuse**	Assistant Professor and Consultant Pathologist	Aga Khan University, Nairobi
**Patricia Okiro**	Consultant Pathologist	Aga Khan University, Nairobi
**Esperanca Sevene**	Professor of Clinical Pharmacology	Centro de Investigação de Saúde de Manhiça
**Helena Boene**	Junior Investigator	Centro de Investigação de Saúde de Manhiça
**Corssino Tchavana**	Data Manager	Centro de Investigação de Saúde de Manhiça
**Paulo Chin**	Clinician & Data Collection Manager	Centro de Investigação de Saúde de Manhiça
**Eusebio Macete**	Director of Centro de Investigação de Saúde de Manhiça	Centro de Investigação de Saúde de Manhiça
**Donna Russell**	Biobanking Consultant	Donna Russell Consulting
**Peter von Dadelszen**	Professor of Global Women's Health	King's College London
**Laura Magee**	Professor of Women's Health	King's College London
**Lucilla Poston**	Professor of Maternal and Fetal Health	King's College London
**Sophie Moore**	Senior Lecturer, Global Nutrition	King's College London
**Rachel Tribe**	Reader of Women's Health	King's College London
**Andrew Shennan**	Consultant Obstetrician & Professor of Obstetrics	King's College London
**Lucy Chappell**	NIHR Research Professor in Obstetrics	King's College London
**Sean Beevers**	Senior Lecturer in Air Quality Modelling	King's College London
**Ben Barratt**	Senior Lecturer in Chinese Environment	King's College London
**Guillermina Girardi**	Chair in Women's Health	King's College London

**Meriel Flint O'Kane**	PRECISE Programme Manager	King's College London
**Amber Strang**	PRECISE Research Coordinator	King's College London
**Marina Daniele**	PRECISE Midwife Researcher	King's College London
**Rachel Craik**	PRECISE Senior Research Coordinator	King's College London / University of Oxford
**Joy Lawn**	Professor of Maternal, Reproductive & Child Health	London School of Hygiene and Tropical Medicine
**Hannah Blencowe**	Assistant Professor of Maternal, Reproductive & Child Health	London School of Hygiene and Tropical Medicine
**Veronique Filippi**	Professor of Demography and Epidemiology	London School of Hygiene and Tropical Medicine
**Prestige Tatenda Makanga**	Senior Lecturer, Surveying and Geomatics Department	Midlands State University
**Umberto D'Alessandro**	Director of MRC Gambia and Professor of Infectious Disease	MRC Unit The Gambia at LSHTM
**Anna Roca**	Infectious Disease Epidemiology	MRC Unit The Gambia at LSHTM
**Melisa Martinez-Alvarez**	Health Systems Research	MRC Unit The Gambia at LSHTM
**Hawanatu Jah**	Gynaecologist-Obstetrician, PRECISE Clinical Lead	
**Alison Noble**	Professor of Biomedical Engineering	University of Oxford
**Aris Papageorghiou**	Consultant Obstetrician & Associate Professor	University of Oxford
**Judith Cartwright**	Professor of Reproductive Cell Biology	St George's, University of London
**Guy Whitley**	Professor of Cell Biology Research	St George's, University of London
**Sanjeev Krishna**	Professor of Molecular Parasitology and Medicine	St George's, University of London
**Marianne Vidler**	Global Health Researcher, PRE-EMPT Manager	University of British Colombia
**Beth Payne**	Postdoctoral Fellow, Global Women's Health	University of British Colombia
**Larry Li**	Database Manager	University of British Colombia
**Sumedha Sharma**	Process Evaluation Specialist	University of British Colombia
**William Stones**	Professor of Public Health, Obstetrics & Gynaecology	University of Malawi

E. Background & rationale

Annually, pregnancy hypertension, fetal growth restriction (FGR) and stillbirth unrelated to intrapartum events are associated with 46,000 maternal and 2.5 million fetal, neonatal and infant deaths [1]. Over 99% of these deaths occur in low- and middle-income countries (LMICs) and over half in sub-Saharan Africa [1]. Major morbidities complicate about 20-fold more pregnancies and infancies than lives lost, resulting in 50 million lives that are threatened and altered by these disorders. This disparity in outcomes between LMICs and high-income countries (HICs) represents a human rights issue and provides the opportunity to intervene to enhance development through improved survival and 'thrival' of each community's greatest resource, their people.

In more developed countries like the UK, we know that these three pregnancy complications are caused by problems with the afterbirth (placenta) and we know quite a lot about how they develop and complicate pregnancies [2]. In contrast, in sub-Saharan Africa, we know very little about how and why these placental conditions occur. This is especially complex as women in Africa often have many other challenges: limited diets that change with the seasons, chronic infections such as HIV or malaria, acute infections like Ebola, limited autonomy of decision-making and life in communities that are prone to either flooding or drought and are remote from health facilities.

Therefore, the pathways to pregnancy complications are probably very different for these women in sub-Saharan Africa, compared with women living in the UK. Yet, these women and their babies bear most of the burden of death and illness related to pregnancy complications. PRECISE is designed to address this area of neglected global health research.

The widely-cited failure within the Millennium Development Goals to adequately reduce infant deaths and reverse maternal health inequalities, prompted incorporation of new goals in the 2016 UN Sustainable Development Goal 3 to 'ensure healthy lives and promote well-being for all at all ages':

- By 2030, reduce the global mortality ratio to less than 70 per 100,000 live births
- By 2030, end preventable deaths of newborns and children under 5 years of age, with all countries aiming to reduce neonatal mortality to at least as low as 12 per 1,000 live births and under-5 mortality to at least as low as 25 per 1,000 live births

This is underpinned by WHO Global Strategy for Women's, Children's and Adolescents' Health. In the maternal-fetal-newborn research field, PRECISE has been funded to develop a network of sub-Saharan-, North American-, European, and UK-based scientists that will capacity-build through the investigation of pathways to disease that result in the placental complications of pregnancy. Designed to strengthen research capacity in Africa through a shared research project, PRECISE will learn about women in a 360° manner (Figure 2), including their socio-geographical, environmental and physical and mental health contexts and will create a biorepository that complements replete clinical and epidemiological data.

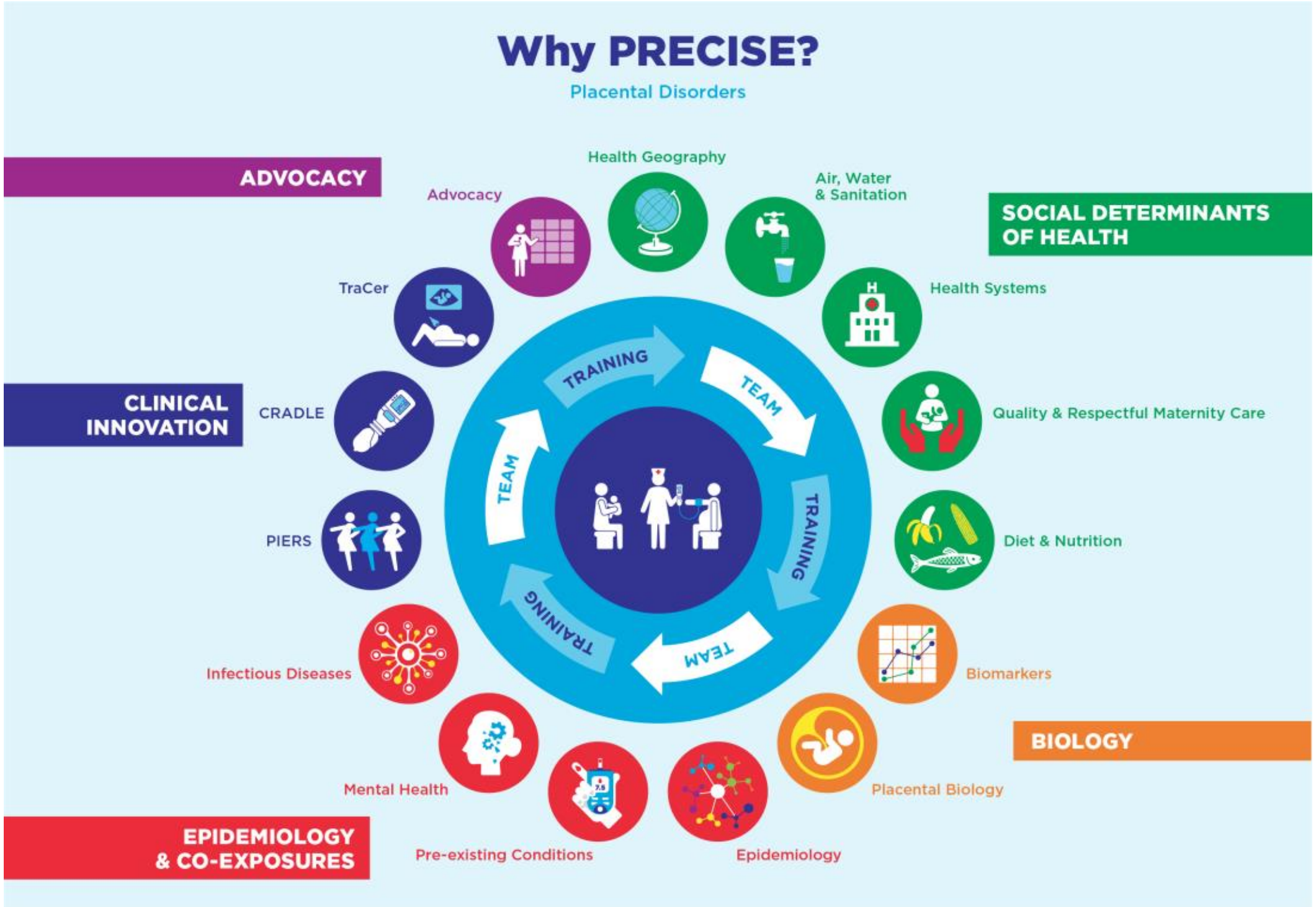

Figure 2: A holistic approach to pregnancy research

CRADLE, Community blood pressure monitoring in Rural Africa: Detection of underlying Pre-eclampsia (blood pressure device); WOICE, WHO Maternal Morbidity Working Group tool; *PIERS*, Pre-eclampsia Integrated Estimate of RiSk (time-of-disease outcome prediction model and associated app); *TraCer*, TransCerebellar diameter (ultrasound app to automatically detect and measure transcerebellar diameter to estimate gestational age)

Biological samples and associated phenotypic information will be collected from a prospective cohort of up to 10,000 pregnant women across three countries in Africa: The Gambia, Mozambique, and Kenya. In addition, we anticipate 20% of these pregnant women will present at clinic or hospital with pregnancy complications. Samples will be collected at booking and at subsequent visits, including delivery and post-delivery. An additional 1,800 non-pregnant women of reproductive age will also be enrolled as control subjects and samples collected at one timepoint.

The samples will be used to test for biomarkers as predictors of important maternal and fetal outcomes and will include genomic, proteomic, nutritional, hormonal and inflammatory markers. All samples remaining after planned analyses for the initial project are done will be used to establish a biorepository (PRECISE biorepository) to facilitate and accelerate future discoveries on maternal, fetal and neonatal health. The biorepository will be managed, governed and owned by each country.

F. Objectives of PRECISE

The broad objectives for The PRECISE Network cover all strategic areas of the programme: research capacity building, global maternal and child health research, partnership building and advocacy *(See Appendix 1: PRECISE Theory of Change).*

1. Build individual and institutional research capacity across Africa and the UK through a shared pregnancy research programme of work *(See Appendix 2: PRECISE Research Capacity Building Framework).*
2. Develop a unique cohort of biologically and contextually characterised pregnant and non-pregnant women of reproductive age in East (Kenya), West (The Gambia) and Southern

(Mozambique) sub Saharan Africa to support research into placental disorders (hypertension, fetal growth restriction and stillbirth) in the region.

3. Build sustainable, equitable partnerships across the individuals and institutions in The PRECISE Network, ensuring leadership and autonomy in research strategy and delivery across the collaborators.
4. Embed PRECISE in the global maternal and child health landscape across the areas of research, health service providers, NGOs, industry and national and international policy to maximise the contribution of PRECISE to the attainment of SDG 3 through broad advocacy and engagement *(Appendix 3: PRECISE Advocacy Framework).*

The research outcomes planned within objective 2 (as above) are detailed as follows:

2.a. To develop a unique cohort of pregnancies affected by placental disease and assess the prevalence of these disorders in women attending antenatal care in centres representative of urban and rural communities in three sub-Saharan African countries.

2.b. To develop cohorts of women with unselected pregnancies and non-pregnant women of reproductive age, for comparison. These cohorts will provide appropriate data with which to compare the context of women and their biology as they have pregnancies complicated by placental disorders, or not. Sufficient culturally and geographically relevant data do not exist to identify pathways to pregnancy resilience or vulnerability, considering women's burden of infectious, mental and/or non-communicable disease. Existing control data have been almost uniformly from more-developed countries in Europe, North America and Australasia.

2.c. To investigate environmental, biological, epidemiological, clinical, social/cultural and health system factors affecting the ability to understand, prevent and manage the effects of placental diseases for African women and their families.

2.d. To investigate the potential for introduction of novel methods to assist the prevention, diagnosis and management of placental disorders in sub-Saharan Africa. Such methods could be new diagnostics based on the agnostic proteomic screening of samples from women with or without pregnancy complications. New pathways to disease may be identified that could be circumvented with novel therapeutics. In addition, the role of culturally and geographically relevant interventions (e.g. faith-based discussions or roads and bridges) may be emphasised as critical health interventions.

G. Research settings

The PRECISE sites have been chosen in locations with research excellence and where data collection will produce representative cohorts of West, East and Southern African women. Given the transition towards increasingly urban populations in Africa and elsewhere in less-developed countries, PRECISE has been designed to recruit women living in both urban and rural settings in the three geographies (Figures 3-5).

To maximise the quality of the biorepositories, each country's selection of sites was undertaken to ensure rapid access to definitive biorepository facilities.

The Gambia (Figure 3)

Our primary partner in The Gambia is the MRC The Gambia Unit at LSHTM (PIs: Umberto D'Alessandro & Anna Roca). The field research will occur at the Farafenni field station (urban primary health centre [PHC] & hospital) and associated rural PHCs in Illiasa and Ngayen Sanjal.

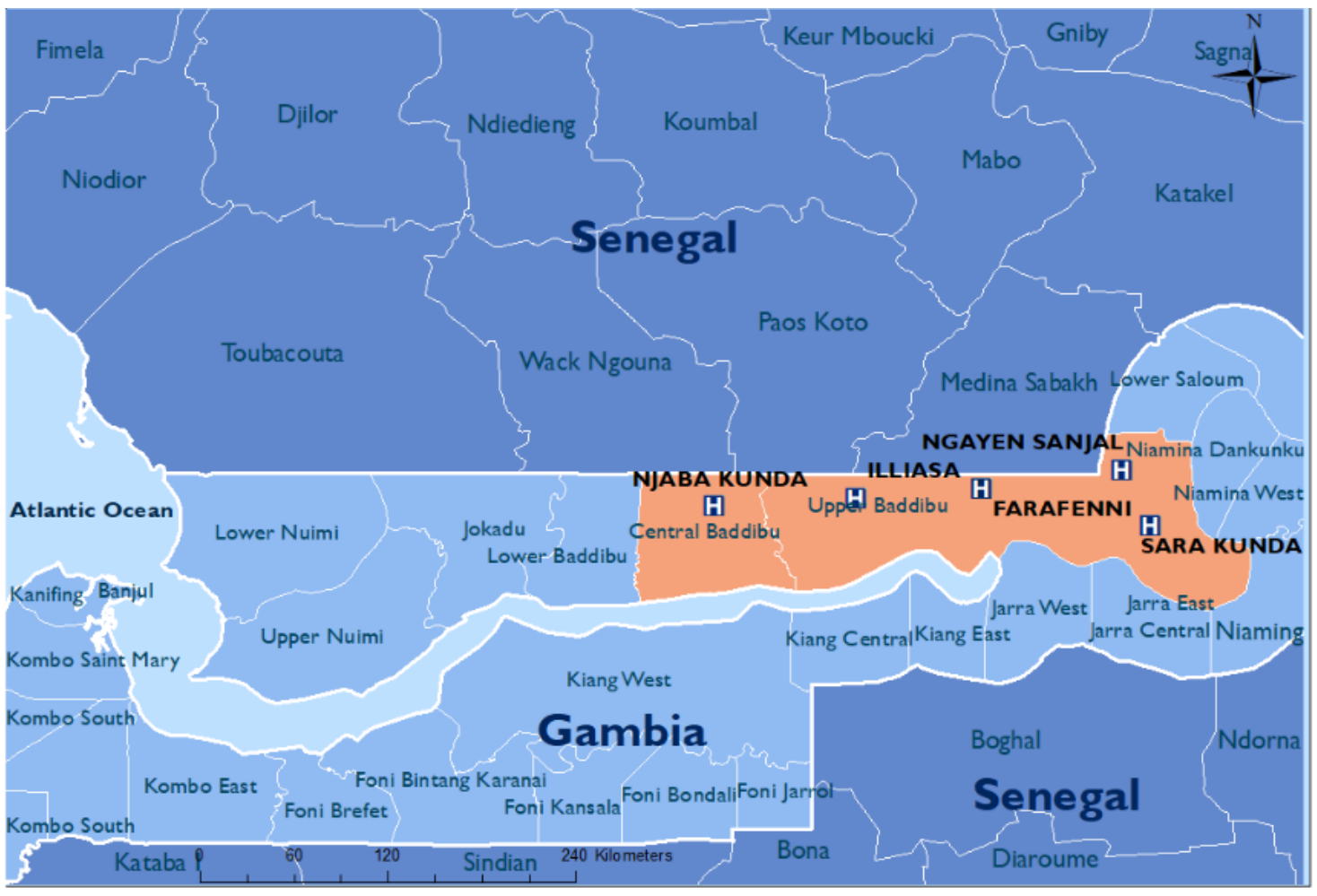

Figure 3 Gambian sites

Kenya (Figure 4)

Our primary partner in Kenya is the Aga Khan University (East Africa) (AKU) (PIs: Marleen Temmerman & Angela Koech). The field research will be conducted through the Mombasa field station, with field activity in Mariakani Subcounty Hospital (urban) and Rabai Health Centre (rural).

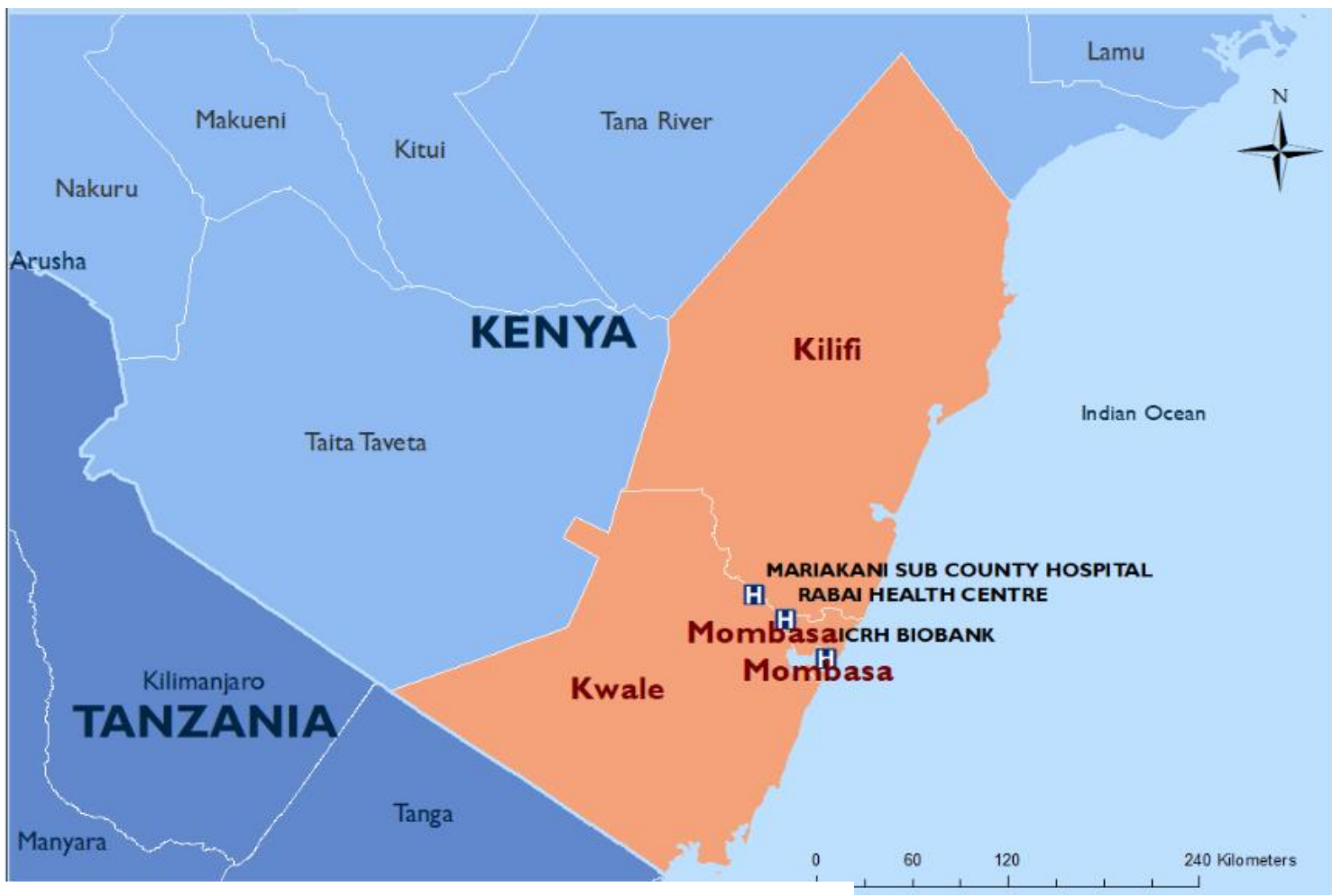

Figure 4 Kenyan sites

Mozambique (Figure 5)

Our primary partner in Mozambique is the Centro de Investigação de Saúde de Manhiça (CISM) (PI: Esperança Sevene). The field research will occur in the Manhiça District Hospital (primarily urban population) and Xinavane Rural Hospital (primarily rural population).

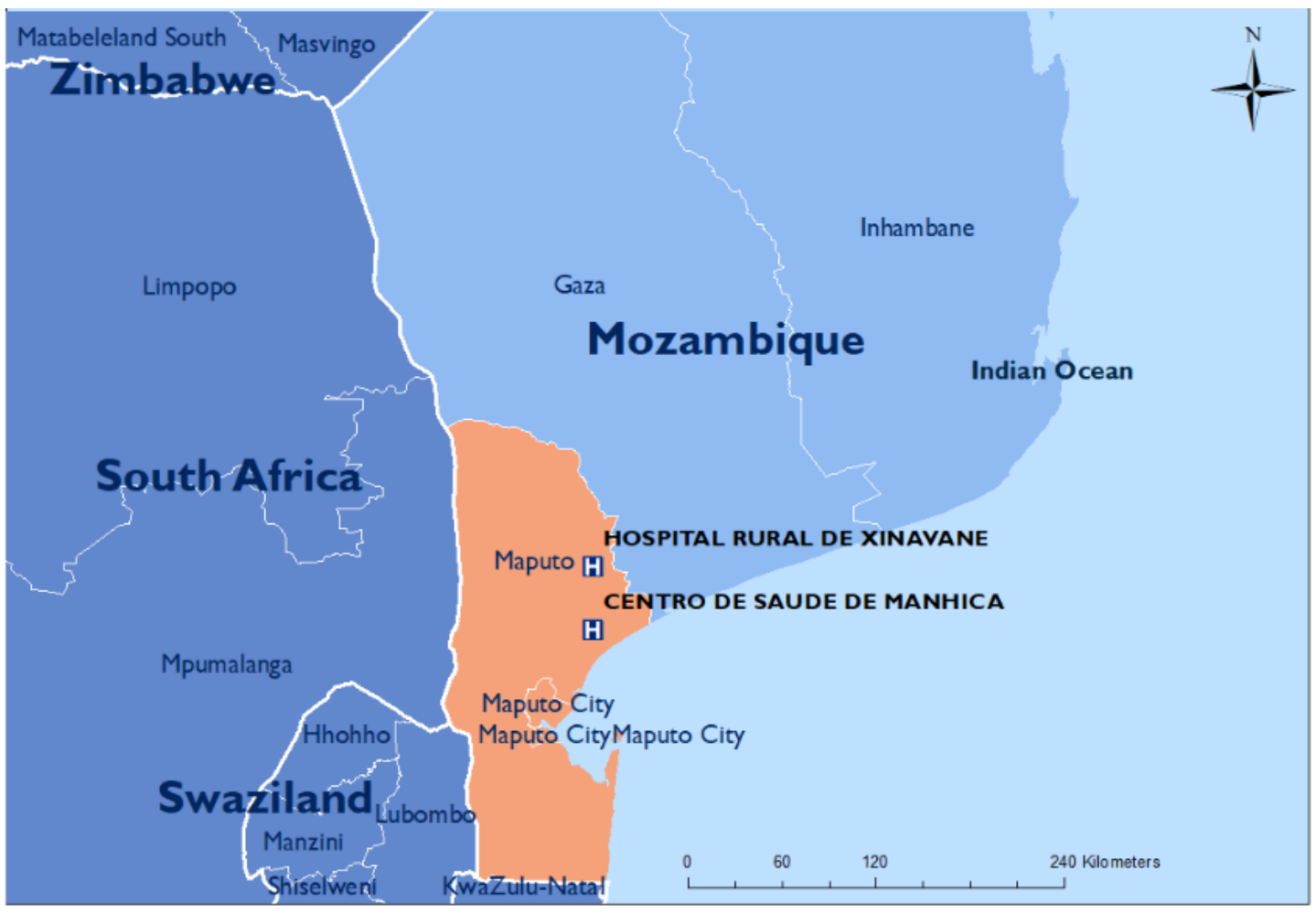

Figure 5 Mozambican sites

H. The women

While PRECISE is designed to answer specific research questions, a major legacy will be the highly-phenotyped representative cohorts of West, East and Southern African women and the creation of an incomparable Africa-based biorepository for future hypothesis-generated and generating research. The core activity will be to collect environmental, social, geographical, demographic, clinical and biological data related to three populations of women.

Consent process

Women enrolled in the PRECISE Network Study will have an opportunity to read or be read the informed consent in their native language and have an opportunity to ask the research co-ordinator any questions they may have. Consent will be confirmed with the participant's signature or a thumbprint. In the absence of a signature, a witness (other than the member of the research team obtaining consent) will be asked to sign. Finally, the member of the research team obtaining consent will sign the form. Specific information sheets and consent forms will be used for different cohorts:

- Cohort 1 (Non-pregnant women of reproductive age) - Appendix 9a
- Cohort 2 (Unselected pregnant women) - Appendix 9b
- Cohort 3 (Pregnant women with high blood pressure or a small baby or suspected or diagnosed COVID-19) - Appendix 9c
- Cohort 4 & 5 (Pregnant women with a known fetal death or following a stillbirth) - Appendix 9d

Vulnerable populations

This study involves pregnant women and newborns. In most sites, potential participants do not speak English. In these sites, consent will be taken in the local language to ensure that the potential participant understands the research. Every effort will be made to ensure their voluntary participation. It will be explained that participation is voluntary and can be terminated at any time without reason and without any penalty. If the potential participant has any questions, they will be

answered in their native language to ensure that they understand the research and their potential role in it.

Confidentiality

To ensure privacy, all the interviews and consenting will take place in a private room at the local health facility. Hard copies of the study-related forms will be stored in a locked cabinet in a storage room under supervision of the principal investigators. Electronic records will be stored in password-protected computers and tablets and only approved study personnel will have access to entire level of information (i.e. technicians will have access only to a subset of data, thus reducing the risk for major level breach). Access to the entire dataset will be restricted to the main PI and the site PIs, to keep a strict control of the data. All specimens and associated phenotypic data will be de-identified and given a unique participant identifier code and no personal information will be stored in the specimen tracking data management system.

Community engagement

The sites that are participating in the PRECISE Network have been working in these communities for many years and have built strong relationships of trust with their communities. The local PRECISE leadership and study teams have informed participating communities of the study and, particularly, had in-depth discussions on the best approach within a given community for the collection of samples that may have cultural or religious significance such as maternal blood, cord blood and placental samples. In addition, the team has discussed with communities the best approach for collecting specimens immediately following childbirth (cord blood and placental tissue) and will return the placenta to the family (if that is their wish) after it is weighed and photographed and small samples are taken from it.

Community engagement activities will be conducted in all study sites to ensure the women and communities in which they live are aware of the PRECISE programme of work. A brief overview of planned activity is presented in Table 2.

Table 2 Community engagement

	The Gambia	Mozambique	Kenya
Stakeholders to be engaged	Faith leaders<br>Non-religious community leaders<br>Women attending ANC	Community leaders<br>Pregnant women<br>Women of reproductive age<br>Mothers and mothers-in-law<br>Partners<br>Stakeholders (Ethical committees, investigators, policy makers)<br>Nurses	Health care workers<br>Community leaders<br>Community health volunteers (CHV)<br>Community-wide meetings<br>Pregnant women, partners, family members
Methods of engagement	Go out into the community to engage with community leaders<br>Open days at health facilities to explain to the women attending ANC	Health talks in the health facility<br>Community meetings<br>Informal conversations<br>Discussion boards	Health talks (baraza) or videos on loop at ANC<br>Community meetings (this will tag on to existing community-based meetings)

			Additional community meetings may be convened for target audiences (pregnant women, mothers, other in-laws, partners)
Key messages	Introduction to research<br>Introduction to informed consent<br>When/how/why biobanking	Assess the acceptability of the biobank<br>Promote early disclose of pregnancy and early attendance to the antenatal clinic (ANC) visits.<br>Promote birth preparedness and hospital delivery<br>Identification of a health unit to give birth in<br>Identification of skilled birth attendants	Introduction to research<br>Introduction to informed consent<br>When/how/why biobanking
Frequency of engagement	Sensitisation activities will occur before recruitment starts and continue during the recruitment period<br>At the end of the study, we will communicate the results to the community following the same procedures	Health talks will be conducted twice a week during the morning with pregnant women in the health facility<br>In the community meetings with specific groups will be conducted<br>One meeting per month with the stakeholders	Initiate activities two months before data collection.<br>Activities will be heightened in communities closest to the health facilities as they will have the largest number of women from these areas<br>Activities will occur throughout the study. Each community will be approached roughly once per month

I. Participant cohorts, data and sample collection

General points related to data collection

As described in Figure 2 (above), the PRECISE research programme will collect 360° data that place women in their social, geographic, health services, nutritional and chronic disease contexts, as well as collecting important biophysical and biomarker data that will explain risks for, and pathways to, placental disorders and other complications of pregnancy.

Using mixed methods, we will assess women in terms of their beliefs and practices around pregnancy, access to care and the acceptability of novel diagnostic tools and interventions. Similarly, we will assess these factors in men's groups, mothers and mothers-in-law, faith and community leaders, and health service providers and administrators. Much of this work has already

been completed in Mozambique during the CLIP trial feasibility study [3-6]. This activity will be supplemented in Mozambique and replicated in the Gambia and Kenya.

Non-clinical data will relate to their nutritional status, demographics and their natural and built environment, as well as available health services (and assessment of the quality of care provided by them). All data will be entered into the PRECISE REDCap data platform. These data will largely be collected only during the enrolment visit for all women, whether non-pregnant or pregnant.

Clinical data will be collected using the REDCap platform. At enrolment, these data will include limited information about past obstetric, medical, and surgical history for all women. For pregnant women, best estimates of pregnancy dating will be obtained at the first visit. At all visits, women will have their weight, BP and SpO_2 measured. At all visits during the COVID-19 pandemic, we will ask questions related to COVID-19 symptoms.

Specific clinical tools to be tested and validated within PRECISE include: (i) the TraCer (transcerebellar diameter) app that will date all pregnancies using the highly-conserved and growth restriction-resistant transcerebellar diameter (separate in-country, sub-project-specific ethics approval for the development and utility testing of the app is being sought) (pregnancy only); (ii) the CRADLE VSA semi-automated and validated BP device will be used for all clinical measurements of BP in the study [7-13] (non-pregnancy and pregnancy); (iii) pulse oximetry will be used to assess the impact of cardiorespiratory disease [14-20] (non-pregnancy, pregnancy and neonates); (iv) the PIERS On the Move (POM) platform to provide time-of-disease risk estimates to hypertensive pregnant women using the PIERS (Pre-eclampsia Integrated Estimate of RiSk) models [14,21-34] (non-pregnancy and pregnancy); and (v) WOICE, the WHO Maternal Morbidity Working Group Tool [35-38] at visits in the third trimester.

Standards for the use of the CRADLE BP device, pulse oximetry and TraCer platform will be detailed in the relevant portions of the clinical SOP.

Cohort 1: Non-pregnant women of reproductive age (Figure 6)

In each country, 50 women will be recruited each month for one year, to provide a final national sample size of 600 non-pregnant women of reproductive age. These women will provide culturally, ethnically and spatially-relevant control data with which to compare women with normal and complicated pregnancies (PRECISE Objective 2b). Recruitment will occur throughout the year to provide data throughout the seasons, with the associated fluctuations in geographical (due to seasonal migration) and infectious disease burdens of risk.

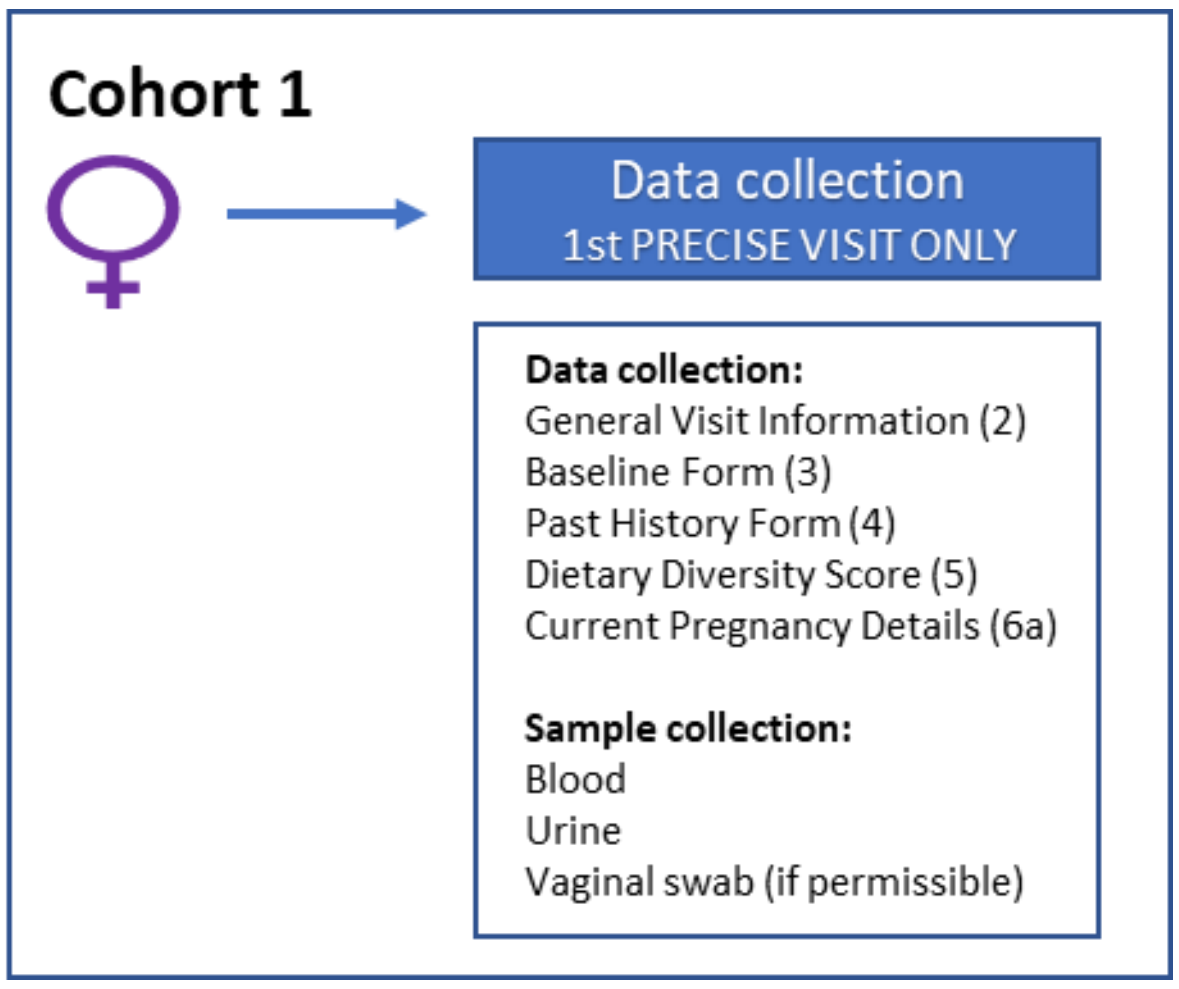

Figure 6 Flow and sampling of nonpregnant women of reproductive age

Cohort 1: Inclusion Criteria

- Attending facility for the care of someone else (e.g. infant vaccinations) or their own care (family planning assessment).
- Not pregnant by self-report and confirmed by a pregnancy test.
- Not pregnant within the last six months by self-report (including miscarriage, termination, stillbirth or live birth).
- Aged 18-49 years.

Cohort 1: Exclusion criteria

- Has already been approached and declined participation in PRECISE.
- Pregnant, confirmed by a pregnancy test.
- Pregnant within the last six months by self-report.
- Aged <18 or >49 years.

These women will be approached when attending the facility for the care of someone else (e.g. for infant vaccinations) or for their own care (family planning assessment) and asked to provide data and samples at a single time point. Non-clinical and clinical data as well as biological samples will be collected from consenting non-pregnant women of reproductive age opportunistically during the first year of the clinical phase of the project.

At the encounter, consenting women will be asked to provide:

- Demographic, non-clinical and clinical data (see Section I & Appendix 4).
- Blood, urine and vaginal swabs (see Section J & Appendix 6).

Cohort 2: Unselected pregnant women (Figure 7)

These women will be approached when visiting the clinic for antenatal care and will be asked to provide data and biological samples to derive normative ranges for pregnant women in less-developed countries (PRECISE Objective 2b). We will recruit women age 16*- 49 years-old (*as pregnant adolescents are at increased risk of placental disease)[39]. For recruitment of women aged 16 and 17 years old we will be guided by the consent and assent processes advised by in-country ethics boards. Research partners in the study countries have advised that pregnant women aged 16 years and over will not require parental consent to participate but can provide consent themselves.

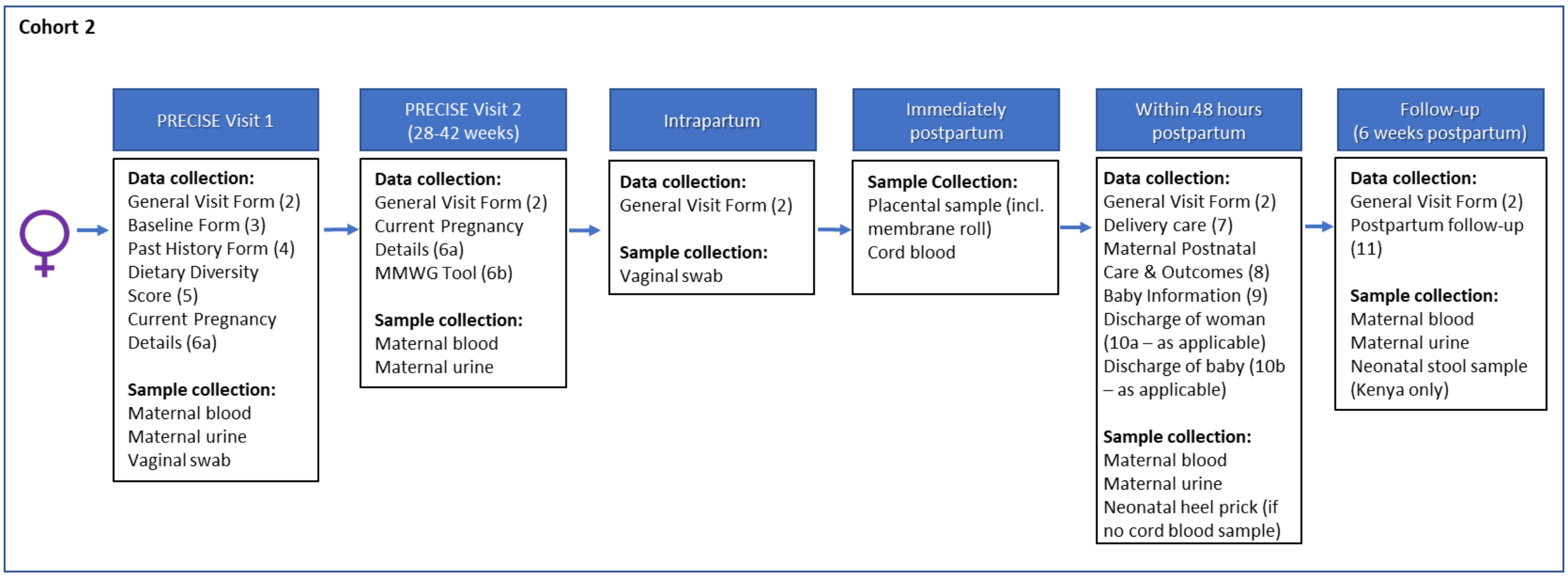

Figure 7 Flow and sampling of unselected pregnant women

Cohort 2: inclusion criteria

- Attending facility for their own care or for the care of someone else.
- Pregnant.
- Aged 16-49 years.
- Have not been referred from another facility.

Unselected Pregnancy Cohort: exclusion criteria

- Has already been approached and declined participation in PRECISE.
- Not pregnant.
- Aged <16 or >49 years.

- Does not plan to deliver at this facility.

Study recruitment (first study visit)

It is anticipated that the median gestational age at booking in each country will be about 24-27 weeks of pregnancy (unpublished data from the Community-Level Interventions for Pre-eclampsia [CLIP] trials).

Whenever possible, at the booking visit women will be approached for participation. Consenting women will have their pregnancies dated using, in order of accuracy, ultrasound dating (recognised to be very uncommon in these settings), certain last menstrual period, estimated last menstrual period and symphysis-fundal height (supplemented, for study purposes, by the TraCer app; the TraCer dating will be used as the gold standard for the academic enterprise). Thereafter, the women will be asked to provide:

- Baseline demographic, non-clinical and clinical data (see Section I & Appendix 4).
- Blood, urine and vaginal swab (see Section J & Appendix 6).

Should a woman not be identified and recruited at her booking visit (and not declined participation), she will be approached at her next visit and, if informed consent is obtained, she will be recruited into PRECISE and the above-described data and samples obtained.

Third trimester

Between 28 weeks' gestation and the onset of labour, and at least four weeks after the study recruitment visit, participating pregnant women will be asked to provide:

- Non-clinical and clinical data (see Section I & Appendix 4), including administration of the WHO maternal morbidity working group ANC tool, WOICE.
- Blood and urine (see Section J & Appendix 6).

Time of disease

If a woman presents at the health facility at a non-PRECISE visit with hypertension, suspected FGR, suspected or confirmed COVID-19 or a stillbirth, the following will be collected:

- Non-clinical and clinical data (see Section I & Appendix 4),
- Blood and urine (see Section J & Appendix 6).

Intrapartum, the following will be collected:

- An intrapartum vaginal swab will be taken in association with a routine, clinically-indicated vaginal examination.

Immediately postpartum

Participating women will be asked to provide:

- Non-clinical and clinical data (see Section I & Appendix 4).
- Placental samples and cord blood (see Section J & Appendix 6).

Within 48 hours postpartum

Prior to discharge home, and up to 48 hours postpartum, participating women will be asked to provide:

- Non-clinical and clinical data (see Section I & Appendix 4).
- Blood and urine (see Section J & Appendix 6).

In addition, permission will be requested to gather the following neonatal data and samples:

- Non-clinical and clinical data (see Section I & Appendix 4).
- Neonatal heel prick (if no cord blood sample collected) (see Section J & Appendix 6).

Six weeks postpartum

At, or soon as possible after, six weeks postpartum, participating women will be asked to provide:

- Non-clinical and clinical data (see Section I & Appendix 4), including administration of the WHODAS 2.0 tool.
- Blood and urine (see Section J & Appendix 6).

In addition, permission will be requested to gather the following neonatal data and samples:

- Non-clinical and clinical data (see Section I & Appendix 4).
- Neonatal stool sample (Kenya only) and heel prick (if not already collected).

Cohort 3 & 4: Pregnant women at time-of-disease (Figure 8)

These women will be approached when visiting the clinic for antenatal care or if they have been referred for care due to suspected or diagnosed placental complications (with either suspected or confirmed pre-eclampsia and/or FGR or as soon as possible after the confirmation of a fetal death) or if they are either tested positive for COVID-19 or suspected COVID-19 positive according to the WHO definition ((i) acute respiratory illness AND having been in contact with a confirmed or probable COVID-19 case or (ii) acute respiratory illness AND a history of travel to or residence in a location reporting community transmission of COVID-19 disease during the 14 days prior to symptom onset or (iii) acute respiratory illness requiring hospitalization AND in the absence of an alternative diagnosis that fully explains the clinical presentation).

We will recruit women age 16*-49 (*as pregnant adolescents are at high risk of placental disease). For recruitment of women aged 16 and 17 years old we will be guided by the consent and assent processes advised by in-country ethics boards. Research partners in the study countries have advised that pregnant women aged 16 and over will not require parental consent to participate but can provide consent themselves.

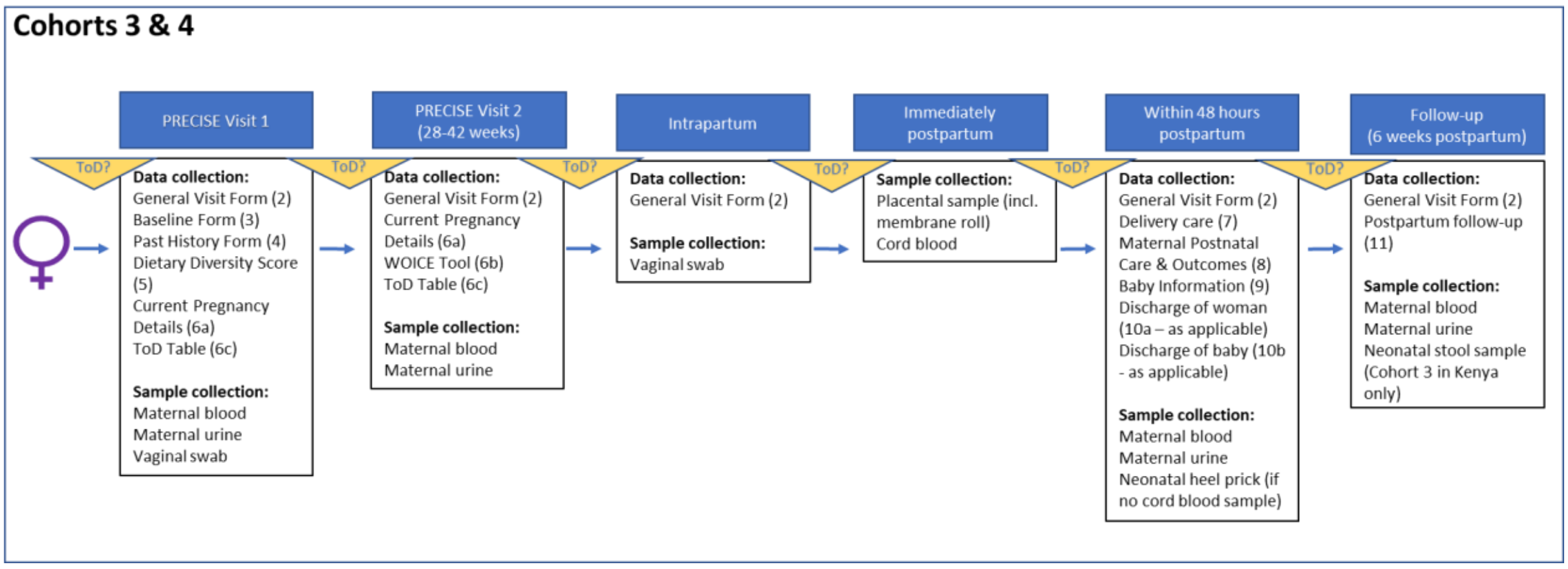

Figure 8 Flow and sampling of time of disease (ToD) pregnant women

Cohort 3: inclusion criteria

- Attending facility for their own care.
- Pregnant.
- Aged 16-49 years.

- By clinical assessment, have hypertension or suspected FGR (without hypertension) and/or tested positive for COVID-19 or suspected COVID-19 cases

Cohort 3: exclusion criteria

- Has already been approached and declined participation in PRECISE.
- Not pregnant by self-report.
- Aged <16 or >49 years.

By clinical assessment do not have hypertension or suspected FGR (without hypertension) and/ do not have COVID-19 symptoms or tested negative

Cohort 4: inclusion criteria

- Attending facility for their own care.
- Pregnant.
- Aged 16-49 years.
- By clinical assessment, have IUFD or stillbirth.

Cohort 4: exclusion criteria

- Has already been approached and declined participation in PRECISE.
- Not pregnant.
- Aged <16 or >49 years.
- By clinical assessment do not have IUFD or stillbirth.

Time-of-disease – Cohorts 3 & 4

With the first diagnosis of pregnancy hypertension $\geq 20^{+0}$ weeks, clinical suspicion of fetal growth restriction, COVID-19 or fetal death *in utero*, women will be approached for recruitment and consent. Health workers will be trained in appropriate and sensitive recruitment of women in the time-of-disease cohort, acknowledging the emotional distress that women may be feeling at this time. Consenting pregnant women will be asked to provide:

- Non-clinical and clinical data (see Section I & Appendix 4).
- Blood, urine and vaginal swab (see Section J & Appendix 6).

Third trimester

Between 28 weeks' gestation and the onset of labour, and at least four weeks after the study recruitment visit, participating pregnant women will be asked to provide:

- Non-clinical and clinical data (see Section I & Appendix 4), including administration of the WHO maternal morbidity working group ANC tool, WOICE.
- Blood and urine (see Section J & Appendix 6).

Intrapartum, the following will be collected:

- An intrapartum vaginal swab will be taken in association with a routine, clinically-indicated vaginal examination.

Immediately postpartum

Participating women will be asked to provide:

- Non-clinical and clinical data (see Section I & Appendix 4).
- A placental sample and cord blood (see Section J & Appendix 6).

Cohort 3 only Within 48 hours postpartum

Prior to discharge home, and up to 48 hours postpartum, participating women will be asked to provide:

- Non-clinical and clinical data (see Section I & Appendix 4).
- Blood and urine (see Section J & Appendix 6).

In addition, permission will be requested to gather the following neonatal data and samples:

- Non-clinical and clinical data (see Section I & Appendix 4).
- Neonatal heel prick (if no cord blood sample collected) (see Section J & Appendix 6)).

Cohort 4 only - Within 48 hours postpartum

Prior to discharge home, and up to 48 hours postpartum, participating women will be asked to provide:

- Non-clinical and clinical data (see Section I & Appendix 4).
- Blood and urine (see Section J & Appendix 6).

In addition, permission will be requested to gather the following fetal/neonatal data:

- Non-clinical and clinical data (see Section I & Appendix 4).

Cohort 3 only - Six weeks postpartum

At, or soon as possible after, six weeks postpartum, participating women will be asked to provide:

- Non-clinical and clinical data (see Section I & Appendix 4), including administration of the WHODAS 2.0 tool.
- Blood and urine

In addition, permission will be requested to gather the following neonatal data and samples:

- Non-clinical and clinical data (see Section I & Appendix 4).
- Neonatal stool sample (Kenya only) and heel prick (if not already collected)

Cohort 4 only –Six weeks postpartum

At, or soon as possible after, six weeks postpartum, participating women will be asked to provide:

- Non-clinical and clinical data (see Section I & Appendix 4), including administration of the WHODAS 2.0 tool.
- Blood and urine (see Section J & Appendix 6).

Cohort 5: Women with stillbirths recruited postpartum **(Figure 8)**

For women who have been referred to the facility with IUFD and for whom it will not be possible or appropriate to approach and consent before delivery, consent to collect retrospective data and samples will be sought postpartum. We will recruit women age 16*-49 (*as pregnant adolescents are at high risk of placental disease) in the delivery suites of the PRECISE facilities. As for Cohorts 3 & 4, for recruitment of women aged 16 and 17 years old we will be guided by the consent and assent processes advised by in-country ethics boards. Research partners in the study countries have advised that pregnant women aged 16 and over will not require parental consent to participate but can provide consent themselves.

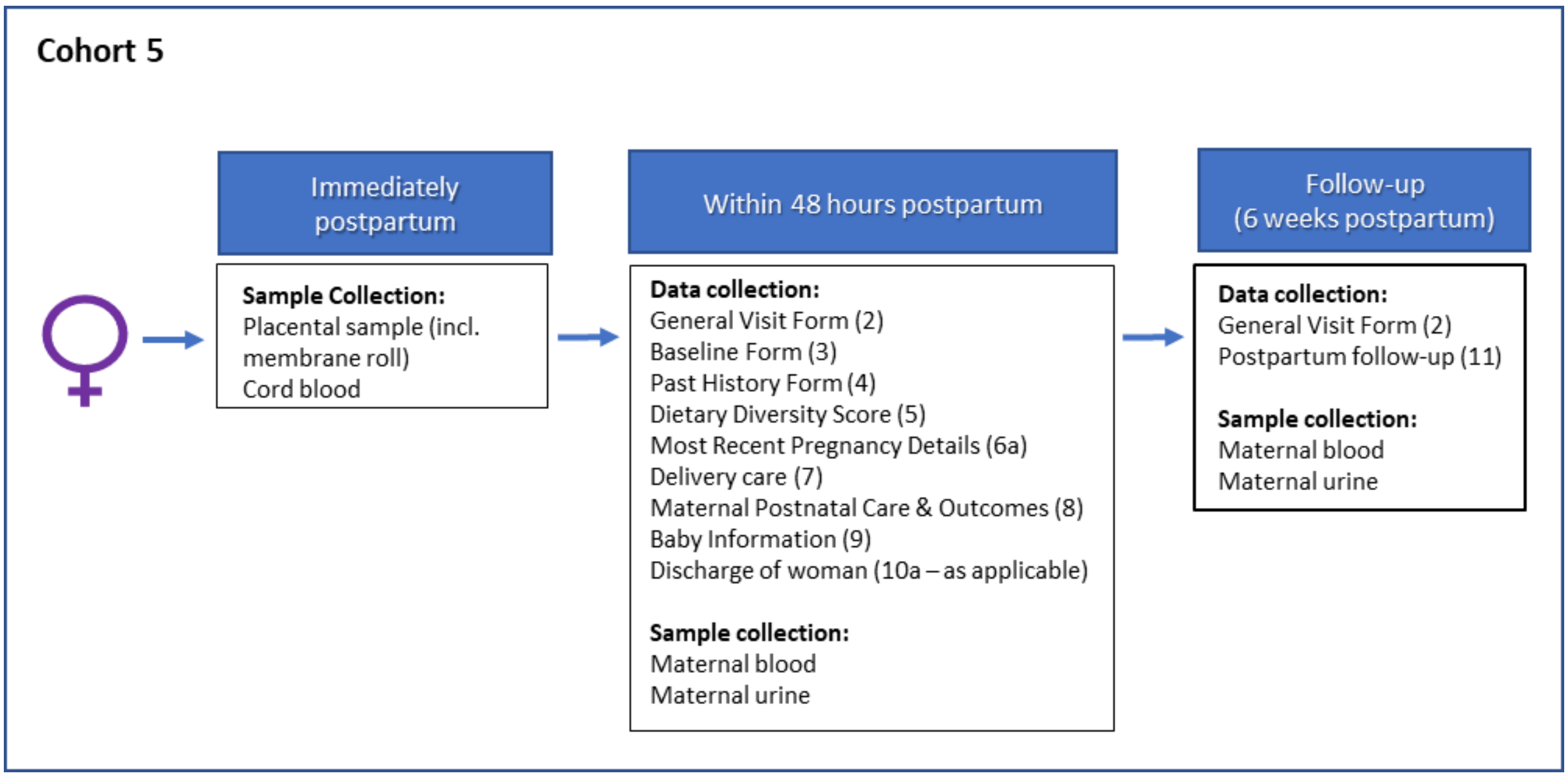

Figure 9 Flow and sampling of women with stillbirths recruited postpartum

Cohort 5: inclusion criteria

- Aged 16-49 years.
- Attends facility with IUFD or recently delivered a stillborn baby.

Cohort 5: exclusion criteria

- Aged <16 or >49 years.
- Not attending the facility with IUFD or has not recently delivered a stillborn baby.

Immediately postpartum

Participating women will be asked to provide:

- A placental sample and cord blood (see Section J & Appendix 6).

Within 48 hours postpartum

Prior to discharge home, and up to 48 hours postpartum, participating women will be asked to provide:

- Non-clinical and clinical data (see Section I & Appendix 4).
- Blood and urine (see Section J & Appendix 6).

Six weeks postpartum

At, or soon as possible after, six weeks postpartum, participating women will be asked to provide:

- Non-clinical and clinical data (see Section I & Appendix 4), including administration of the WHODAS 2.0 tool.
- Blood and urine (see Section J & Appendix 6).

J. Creating the PRECISE biorepository (see Appendix 6 for Standard Operating Procedures)

Collection of samples (Tables 3 & 4)

Blood

Cohort 1: We anticipate that each participant enrolled in the non-pregnant cohort will provide one blood sample to the PRECISE Study at the time of enrolment. The blood draw will be approximately 16mL (roughly equivalent to 1 tablespoon).

Cohort 2: We anticipate that each participant enrolled in the pregnancy cohort will provide at least four blood samples to the PRECISE Study: one at booking; one later in pregnancy between 28^{+1} – 36^{+6} weeks' gestation (at least four weeks after the initial biological sampling); one within 48 hours postpartum and one at or after six weeks postpartum. Each blood draw will be approximately 16mL (roughly equivalent to 1 tablespoon). If the woman presents with a Time-of-Disease condition outside of her routine study visits, an extra blood sample will be taken at this time.

Cohorts 3 & 4: We anticipate that each participant enrolled in the pregnancy at Time-of-Disease cohort will provide from one to four blood samples to the PRECISE Study, depending upon when they present with complications: one at enrolment; and/or one later in pregnancy between 28^{+1} – 36^{+6} weeks gestation (at least four weeks after the initial biological sampling); one within 48 hours postpartum and one at or after six weeks postpartum. Each blood draw will be approximately 16mL (roughly equivalent to 1 tablespoon).

All blood collection is intended to qualify as minimal risk. The total volume and frequency, when considered in the context of the clinical encounter, will not exceed the following parameters (OHRP (45 CFR 46.110):

- For healthy, non-pregnant adults who weigh at least 50kg, the amounts drawn may not exceed 550 mL in an 8-week period and collection may not occur more frequently than 2 times per week
- For other adults and children, the amount drawn may not exceed the lesser of 50 mL or 3 mL per kg in an 8-week period and collection may not occur more frequently than 2 times per week

Cohort 5: We anticipate that participants enrolled in the stillbirth cohort who are recruited postpartum will provide two blood samples to the PRECISE Study: one at enrolment and one at or after six weeks postpartum. Each blood draw will be approximately 16mL (roughly equivalent to 1 tablespoon).

Urine

Up to 20mL of urine will be collected at the same time the blood sample is collected for Cohorts 2, 3, and 4 . If the woman presents with a Time-of-Disease condition outside of her routine study visits, an extra urine sample will be taken at this time. For Cohort 1 (the Non-Pregnancy Cohort), one urine sample will be collected once at the time of enrolment.

Vaginal swabs

With explicit additional consent, in Cohort 2,3 and 4 mid-vaginal swabs will be collected at the booking visit and when the women present to deliver. For Cohort 1 (the Non-Pregnancy Cohort), vaginal swabs will be collected once at the time of enrolment. For cohort 5, vaginal swabs will not be collected.

Cord blood

After delivery of the baby, the umbilical cord is clamped and cut. The umbilical cord (including the cord blood within it) is generally discarded along with the placenta. After the cord is clamped and cut, the cord blood will be collected. The amount of cord blood drawn will be approximately 16mL (roughly equivalent to 1 tablespoon).

Placenta

The placenta will be trimmed and weighed and photographed using a digital camera in a fixed position. Eight small coin-sized tissue samples will be taken from the placenta, five 5mm-long

sections taken from the cord and one strip of membrane. Four samples will be processed in formalin for histology and the rest will be flash frozen in liquid nitrogen for proteomic and metabolomic analyses.

Newborn blood

Only if the cord blood collection is missed, 2-3 drops of blood for DNA will be collected from a heel stick prior to discharge from the facility.

Neonatal stool (Kenya only)

The stool will be collected to examine the newborn microbiome. The neonatal stool sample will be collected from the baby's nappy at the 6 weeks postpartum follow-up visit.

The samples will be collected, processed and stored in adherence to the PRECISE Network Biorepository Standard Operating Procedures (SOPs) across all sites. Study personnel at each site will receive initial and ongoing training as needed to ensure SOPs are followed and samples are of the highest quality.

Table 3. Sampling from Cohort 1 (non-pregnant women of reproductive age)

Samples Collected	PRECISE Visit 1	PRECISE Visit 1	PRECISE Visit 2 (28^{+1}-36^{+6})	Intra-partum	Immediately post-partum	Within 48 hrs postpartum	Total Collections
Blood	16 mL						1
Urine	20 mL						1
Vaginal swabs	4 swabs						1

Table 4. Sampling from Cohort 2 (unselected pregnant women) and Cohorts 3 & 4 (women at time-of-disease) and their infants

Samples Collected	PRECISE Visit 1	PRECISE Visit 2	Time-of-Disease (cohort 2)	Intrapartum	Immediately postpartum	Within 48 hrs postpartum	6 weeks postpartum	Total Collections
Blood	16 mL	16 mL	16 mL			16 mL	16 mL	4
Urine	20 mL	20 mL	20 mL			20 mL	20 mL	4
Vaginal swabs	4 swabs			4 swabs				2
Cord blood					16 mL			1
Placental tissue					14 small pieces of tissue, membrane and cord			1
Newborn blood						2-3 drops from heel stick ONLY if cord blood is not collected		1
Neonatal stool							Approximately 100mg	1*

***Cohorts 2 and 3 in Kenya only Table 5.**

Sampling from Cohort 5 (women with stillbirth recruited postpartum)

Samples Collected	Immediately postpartum	Within 48 hrs postpartum	6 weeks postpartum	Total Collections
Blood		16 mL	16 mL	2
Urine		20 mL	20 mL	2
Cord blood	16 mL			1
Placental tissue	14 small pieces of tissue, membrane and cord			1

Sample storage

We plan to create a unique biorepository (PRECISE Network Biorepository) in each country that will store maternal and newborn samples from high-risk populations in low-resource settings. Samples that are not immediately analysed as part of the PRECISE Study will be stored in the repository and made available to investigators for future studies with the goal of facilitating and accelerating discovery of new diagnostics, therapeutics and preventive strategies to improve maternal, newborn and child health outcomes in LMICs. These biorepositories will serve as a valuable in-country resource for building and strengthening research to improve maternal and newborn health across Africa.

Specimen data management system

The PRECISE repositories will use a web-based Laboratory Information Management System (LIMS) for sample tracking.

All samples and their associated data stored in the LIMS will contain a unique participant identifier sample code, which is the key identifier for linking to the clinical data stored in the REDCap database. In no case will a participant's personal health information be attached to any sample or associated data. The link between the participant's personal identifying information and their unique participant number will be stored securely under lock and key or on a password-protected computer at the study site and only the Principal Investigator or his or her designate will have access to this information. As only the Principal Investigator and/or an authorised study coordinator at each local collection site will have access to participants' personal information and will have taken secure measures to ensure the participants' confidentiality, the likelihood of a breach of confidentiality is minimal.

The LIMS meets the requirement of biobank best practices for human biobanking and meets requirements for ensuring secure and confidential electronic data. The LIMS specimen tracking and barcoding system will serve as the primary sample tracking and sample inventory data system.

These systems will provide PRECISE Management with a detailed daily record of participant enrolment and data and sample entry to monitor progress against the project's milestones. Corresponding actions, such as telephone calls, web conferences or site visits will take place within 10 days of detecting a problem at a site, to ensure that appropriate corrective measures are taken.

Ensuring that only authorised PRECISE staff have access to the samples from the time they are collected, processed and then stored in the biorepository freezer will prevent inadvertent access to samples. Freezers will be located in a secure location and monitored 24/7. The Data and Sample Committee (DSC) will oversee access to the samples, which will represent the governing authority of the PRECISE Biorepository. The DSC will follow a clearly outlined mandate, in the form of the

Bylaws, determining who may have access to the samples for research, how samples may be used and how unused samples should be disposed of. The DSC Bylaws' procedures relating to the distribution and use of human samples will be in accordance with the informed consent and national research policies/legislation regulating use of human biological samples.

The sample collection, processing and storage procedures will be performed in a standardised manner based on protocols described in detail in the PRECISE Standard Operating Procedures Manual. It is critically important to ensure that samples are collected, processed and stored in a standardised manner with adequate monitoring of quality control, because sample quality, quantity, and handling can significantly influence the results of microarray and sequencing experiments. Care will be taken to map each site's workflow, so that it aligns with standardised procedures.

Another very important reason for ensuring the samples are collected, processed and stored in a standardised manner is to facilitate the interchange of data and samples with other biorepositories for validation purposes and to enable the comparison of findings across multiple studies utilising samples from harmonised biorepositories. This addresses the challenge of trying to compare "apples to oranges" across similarly focused but otherwise non-standardised studies.

Direct access to the specimen and clinical database servers will be restricted to individuals who are responsible for monitoring and backing up the system and databases. All other access to the database server will be controlled by logical security and occur across a secure network protected by password access and system controls with role-based authorisation, chain of custody and the audit trail.

Ownership and long-term sustainability of the PRECISE biorepository

The PRECISE biorepository will be established in each of the participating sites in The Gambia, Mozambique and Kenya. The ownership of each of these biorepositories will be with a country-based institution. The Data and Sample Committee will manage and run the PRECISE biorepository. Future use of samples in the biorepository will be open to other researchers focused on improving maternal, newborn and child health outcomes, with a preference given to in-country researchers.

The most important capacities for maintaining/sustaining the biorepository are: a data management system that tracks data and metadata of the samples along with associated phenotypic data, standard operating procedures for enrolling/consenting participants, collecting/processing/storing samples, entering data and quality control; sufficient and secure space for long term storage for samples, including -80°C freezers and dedicated shelving for samples stored at ambient temperature; and a governing body (DSC) with clearly defined policies and procedures (bylaws) for ensuring that the samples are used the way they are intended, in accordance with each woman's informed consent.

Governance of the PRECISE biorepository

The DSC represents the governing authority of the PRECISE biorepository. The DSC will follow a clearly-outlined mandate, in the form of the bylaws, describing who may have access to the samples for research, how samples may be used and how unused samples should be disposed. The DSC bylaws' policies and procedures relating to the use of human samples will be in accordance with the informed consent and the national research policies/legislation regulating use of human biological samples. The DSC will meet on a regular basis to review requests from researchers for samples and will approve/deny access to samples according to criteria agreed upon in the Bylaws. Please see templates in Attachments A (Bylaws) and B (Decision Flow Chart).

Sustainability of the PRECISE biorepository

Long-term sustainability of the biorepository is ensured when the samples stored within the biorepository are perceived to be of high quality and accessible to researchers. As the PRECISE biorepository is affiliated with respected in-country academic institutions, we will encourage in-

country researchers to utilise the biorepository as a resource. The goal is two-fold: first to ensure that the samples are used as intended to support research that will lead to improved maternal and newborn health; and second, to promote research and build research capacity in-country. To that end, it is essential that the samples be promoted for use in future studies.

Safeguards that will be applied in case biological samples are transferred abroad

Many studies utilising biological samples are sending biological samples abroad to take advantage of lower costs for high throughput analyses. In addition, it is sometimes a requirement of participation in a large, multi-national research project utilising samples, for all participating sites to send samples to a common facility for analyses. Under these circumstances, the DSC would review the proposed projects and ensure that the samples would be used in accordance with the informed consent and national research policies and legislation.

K. Interactions with the clinical service

As PRECISE will improve the quality of antenatal care provision, with accurate blood pressure measurement, a focus on stillbirth, routine pulse oximetry and screening for acute risk of either self-harm or suicide (through the WOICE tool), the research team recognises that defined clinical responses are incumbent on us. Detailed descriptions of the available testing, treatment and care pathways in each country are provided in Appendix 7.

L. Sample size and general statistical approaches

The primary focus of PRECISE is one/more of:

- Pregnancy hypertension of any type (chronic hypertension, gestational hypertension & pre-eclampsia).
- Fetal growth restriction (defined as birthweight <10th centile for gestational age associated with PlGF <5th centile for gestational age). Other newborns delivered <10th centile for gestational age will provide an additional comparator cohort.

- Stillbirth (defined as the delivery of a dead fetus at ≥20^{+0} weeks gestational age and/or weighing ≥500g).

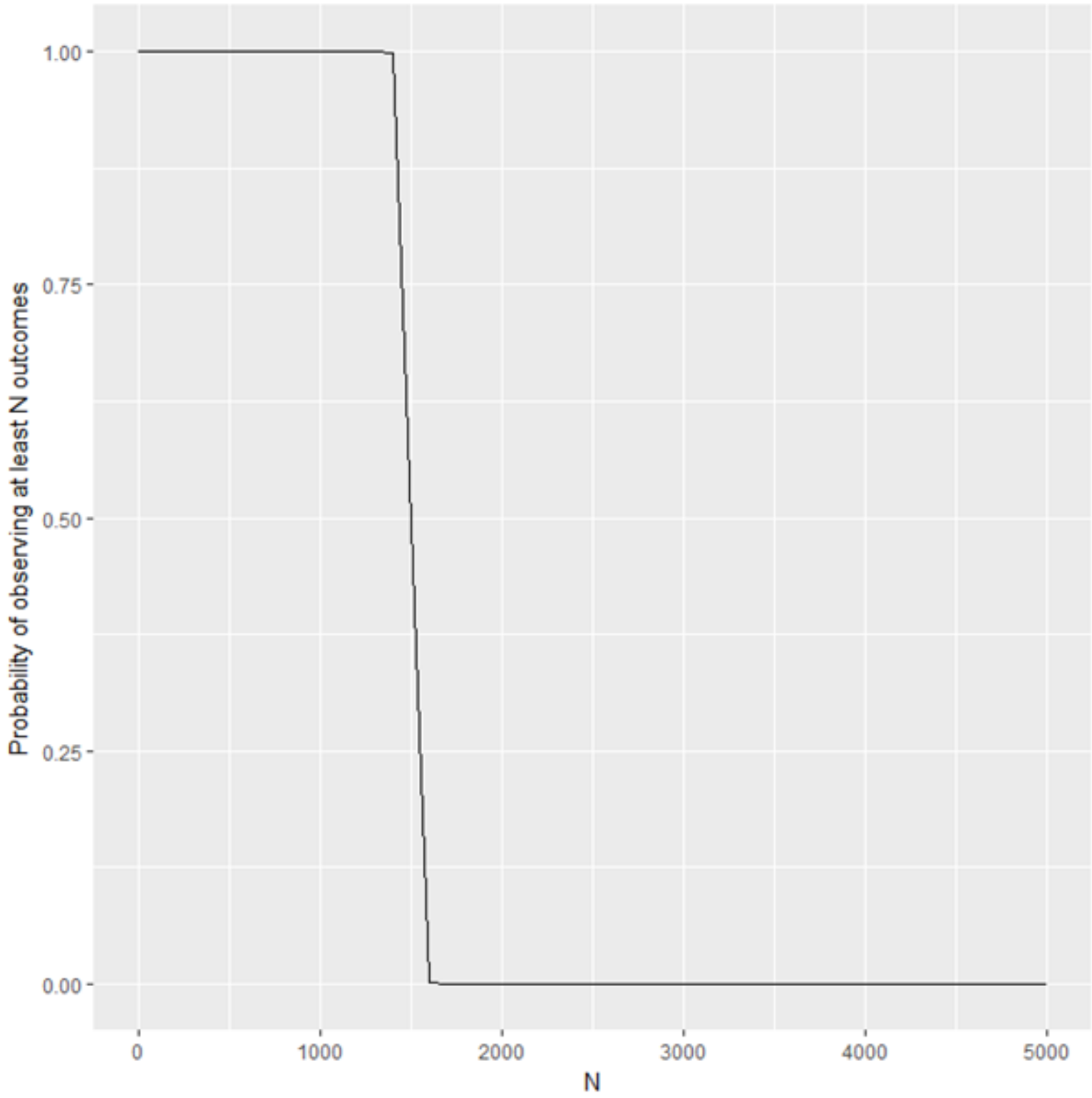

Figure 10 Probability of observing 600 outcomes
Graph for probability of observing at least N outcomes given 15% overall outcome rate (across all countries) and 10,000 total pregnancies. Near 100% probability of getting between 1300 – 1500 cases (if 15% outcome rate is true)

Our objective is to recruit at least 600 women per country whose pregnancy is complicated by at least one placental disorder. We have calculated various sample sizes (within each country) needed to achieve 600 cases of placental disorders/country (Table 5). For example, if the outcome rate is 15%, the sample size requirement is ≈4200 cases/country to be 89.9% certain of identifying ≥600 cases.

Table 5. Sample size calculations

N per country	Assumed outcome rate	Probability of ≥600 cases observed
**12,500**	5%	0.843
**6300**	10%	0.893
**4200**	15%	0.899
**3100**	20%	0.809
**2500**	25%	0.871

PRE-EMPT data suggest that ≈10% of recruited unselected pregnant women will have pregnancies complicated by pregnancy hypertension and 3% will suffer a stillbirth. Approximately 12% will deliver a growth-restricted newborn <10th centile for GA using the INTERGROWTH-21st standard. These numbers are not mutually exclusive; we assume that 20% of the cohort in each country will have a pregnancy complicated by a placental disorder. These numbers will be enriched by the enrolment of previously non-enrolled pregnant women who present at time-of-disease.

We have identified the following secondary foci against which we will examine variables:

- Maternal death (we estimate that ≈50 women will die either during pregnancy or the puerperium period).
- Neonatal death (we estimate that ≈500 newborns will die within 28 days of birth)
- Hyperglycaemia of pregnancy (using AUC ROC analyses of HbA_{1c} and/or glycosylated fibronectin to define an optimal Africa-relevant upper limits of normal that predicts adverse maternal and perinatal events).
- Preterm (<37^{0} weeks) and very preterm (<34^{+0} weeks) birth.

This sample size will provide sufficient power to investigate the complex demographic, geographical, nutritional infectious and biomarker differences and interactions, especially as they relate to stillbirth. Non-parametric analytical methods will be used, with $p < 0.05$ for statistical significance for primary outcomes of each analysis and $p < 0.01$ for all other comparisons.

When appropriate, Kruskal-Wallis ANOVA and multivariable logistic regression analyses will be performed.

In addition, we will undertake sophisticated mediation analyses to understand a known relationship by exploring the underlying mechanism or process by which one variable influences another variable through a mediator variable. Mediation analysis facilitates a better understanding of the relationship between the independent and dependent variables when the variables appear to not have a definite connection. They are studied by means of operational definitions and have no existence apart. A mediation model is one that is designed to identify and explain the mechanism or process that underlies an observed relationship between an independent variable and a dependent variable via the inclusion of a third, mediator, variable. Rather than a direct causal relationship between the independent variable and the dependent variable, a mediation model proposes that the independent variable influences the (non-observable) mediator variable, which in turn influences the dependent variable. Thereby, the mediator variable serves to clarify the nature of the relationship between the independent and dependent variables.

M. The PRECISE database and data management

The COLLECT-compliant PRECISE database

We have the advantage of access to the recently-developed standard pregnancy database (COLLECT), developed by the Global Pregnancy Collaboration (CoLab), within PRE-EMPT. The PRECISE platform will be COLLECT-compliant in terms of data dictionary, to facilitate future international collaborations. The database will be based on the REDCap platform (https://www.project-redcap.org/and be coded, managed and the data analysed, by the highly-experienced and global health-adept team which has co-ordinated the PRE-EMPT initiative.

Many obstetric problems, including pre-eclampsia, are complex syndromes with more than one subtype suggesting differing underlying pathogenic pathways. To unravel these complexities, large, statistically powerful datasets formed by merging many smaller studies are needed. Databases are expensive to design and maintain and often either difficult or impossible to merge due to incompatibility of definitions and format.

Therefore, in collaboration with CoLab, we have created a standard pregnancy database available for local and online use by interested investigators in less-developed countries.

What is provided:

- A dataset that is agreed and defined for the placental disorders of pregnancy, with optional data items available for specific investigators and local study-specific requirements
- Identical data items: collected in the same format using a standard data dictionary

- Data collection to enable retrospective diagnosis using variable definitions by impartial investigators
- Online input and access to data: with a local spreadsheet option where needed
- Security and ethical considerations: each database is fully owned by the investigator
- Collaboration: is enabled but not required
- Global application: high functionality for investigators anywhere in the world
- Updated: as needed while retaining core consistency
- Data management plan

Description of data

Type of study

The new data are being obtained from focus group discussions (FGDs), in-depth interviews (IDIs), geoinformation systems (GIS) mapping for communities and individual-level data related to clinical activities and human blood, urine and placental samples from pregnant and non-pregnant women participating in a cohort study. Pregnant women will be invited to give blood and urine samples up to five times during the time of their pregnancy, delivery and puerperium.

Types of data

The new data will be:

Qualitative: generated from formative qualitative research and community engagement through FGDs with pregnant women, non-pregnant women of reproductive age, adolescent girls and boys, mothers-in-law, matrons, husbands, men's groups and faith and local leaders. In addition, IDIs will provide data from health decision makers at local, regional and national levels.

Quantitative: generated from participant questionnaires, electronic health records and clinical measurements and laboratory-based analyses of human blood, urine, vaginal swabs and placental samples.

GIS: generated through our 9-step method for creating framework data for GIS analysis in less-developed countries: (i) determining what publicly-accessible spatial data are available from existing data warehouses; (ii) consultation with local/national mapping agencies to acquire relevant datasets; (iii) inventory of data from all sources to identify data gaps; (iv) use of freely-available high-resolution satellite data to digitise new vectors extractable from the imagery (e.g., dwellings and roads); (iv) use of appropriately-skilled local personnel with local knowledge to be part of a consensus-based process for data capture; (v) use of GPS co-ordinates from previous household surveys or censuses, where available, as the basis for mapping the location of populations and higher-resolution community boundaries; (vi) use of open geospatial standards for web mapping (e.g. the web feature service) to facilitate for distributed data capture; (vii) use of open standards to document metadata for the created data; (viii) use of independent data checkers to validate captured data (if possible, involving the mapping agency); and (ix) data sharing with mapping agencies to add to their data infrastructure and to make data accessible to other researchers.

Format and scale of data

Qualitative data

Digital voice recorders and hand-written field notes will be used to record discussions during GDs and IDIs. Primary analysis will be conducted in the local language, prior to translation/back translation into English if necessary. Each FGD and IDI will be assigned a unique identifier and photographs taken during data collection and reflection notes attached to transcripts for analysis. All coded transcripts will be cross-checked by the local research

team to resolve or clarify any data misinterpretation. Thematic analysis (combining inductive and deductive approaches) will be performed in-country by the local country team supported by the central team, as required. Using deductive reasoning, the results will be grouped into predetermined categories of key themes related to the discussion guides. During analysis, inductive reasoning will be used to incorporate new and unexpected ideas to produce a comprehensive analysis structure to reflect the richness and variety of responses. Data will be analysed using NVivo 10 software.

Clinical and laboratory data

Clinical data will be collected onto the REDCap PRECISE database using Android tablets and computers and stored on a PostgreSQL database. Core laboratory data will be transmitted into this database from a laboratory management information system (LIMS). The REDCap system (https://www.project-redcap.org/)meets the needs of web-based open-source system for data management.

REDCap database customisation allows us to collect, manage and analyse transactional, case-based data records. It stores information about individual participants and tracks multiple visits including booking, antenatal care, delivery and postnatal care over time. REDCap supports secure online data entry on a standard web browser, or offline data collection on a mobile device and data synchronisation with the server over Wi-Fi connection by end users. Data will be able to be downloaded as CSV or Excel files that can be imported to STATA or other equivalent statistical software.

Routine automated laboratory methods

All laboratory data, such as ELISAs and assays on automated clinical analysers, generate data which will be stored in Microsoft Excel. Data generated on automated analysers and from the plate reader for ELISAs, will be transcribed to Excel, from Ascii files generated. This is efficient given that samples are run in the order of the database (above). Hard copies of results will be retained for checking. After transcription, each result will be checked manually by two separate technicians. Once the dataset is complete, each variable will be range-checked. Data outside the expected range will be verified against the hard copies. Reasons for missing data will be logged using standardised coding. Analytical software includes Excel and STATA (most recent relevant version).

Laboratory and sample management data (Figure 10)

A laboratory information management system (LIMS) is central to the informatics infrastructure that underlies biobanking activities. To date, a wide range of commercial and open-source LIMS systems are available and the decision to opt for one LIMS over another is often influenced by the needs of the biobank clients and researchers, as well as available financial resources.

Until December 2019, the LIMS system Baobab was used for the study. The Baobab LIMS was developed by customising the Bika LIMS software (https://www.bikalims.org) to meet the requirements of biobanking best practices and address the unique challenges of biobanking in LMICs. The Baobab LIMS is open-source and comprises of modules for biospecimen kit assembly, chain of custody, sample management, analysis requests, reporting, inventory management and invoicing.

The Baobab LIMS is based on the Plone web-content management framework and uses the ZODB object database to store data. All the system requirements for Plone are applicable to Baobab LIMS, including the need for a server with at least 8 GB RAM and 120 GB hard disk space. Baobab LIMS is a server-client-based system, whereby the end user can access the

system securely through the internet on a standard web browser, thereby eliminating the need for standalone installations on all machines.

The Baobab LIMS is being adopted widely across Africa to support large-scale initiatives including the West African Biobank Network and The African Reference Laboratory.

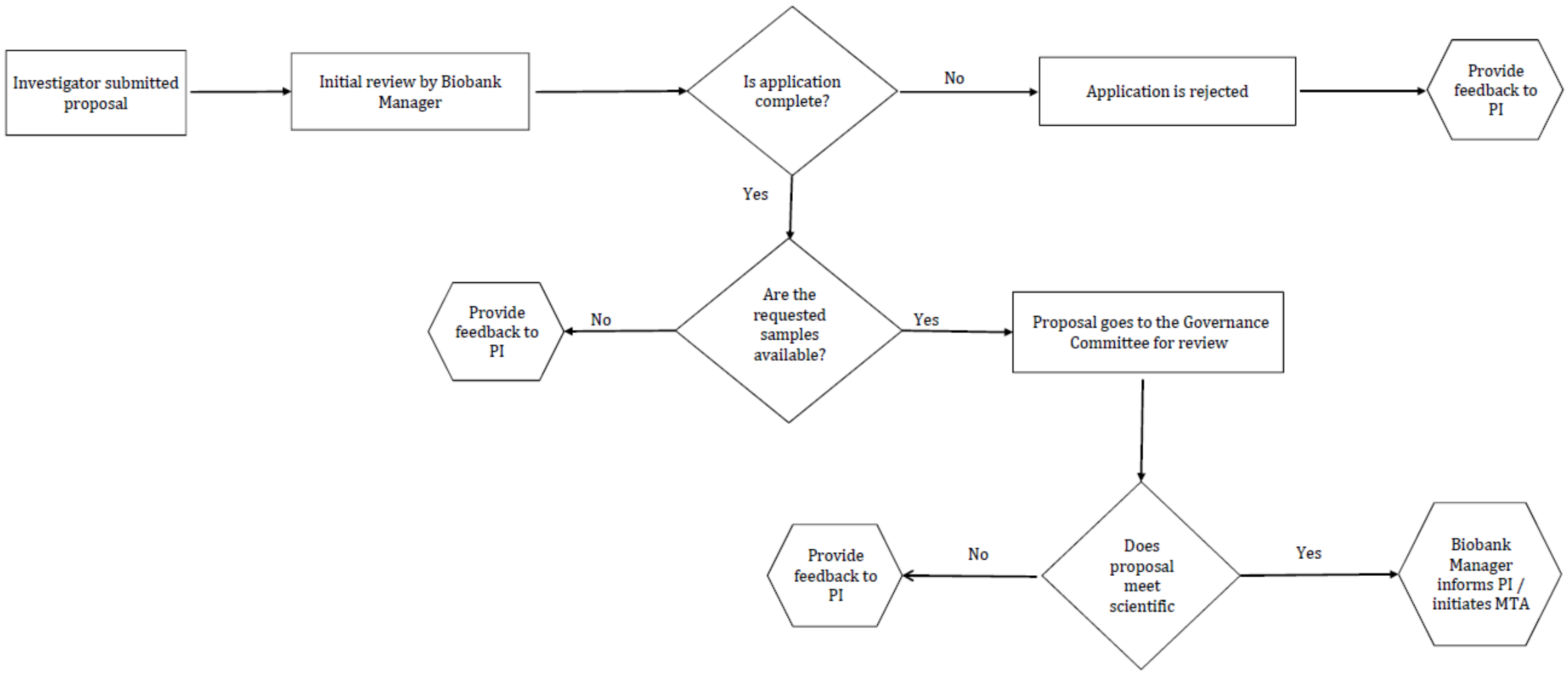

Figure 11 Data management plan for the biorepository

From January 2020, the LIMS system OpenSpecimen (https://www.openspecimen.org/) is being used. This is an open source biorepository platform developed by Krishnagni Solutions and used in more than 60 research centres in the world. OpenSpecimen can track all types of biospecimens from collection to utilisation for prospective biobanking and multi-site clinical studies. OpenSpecimen is a Tomcat-based web system and can be hosted locally in each country using the existing IT resources and securely accessed with a web browser.

.

Laboratory data collection/generation

Methodology for data collection/generation

Routine lab/ELISA data will be collected in accordance with ISO 9001:2008 for quality systems and a platform like TickIT for software development. UBC has an ISO 27001 certificate. UBCs solutions conform to relevant FDA, NIH and HL7 standards, guidelines and recommendations.

Data quality and standards

All sample identities, clinical characteristics and outcome status will be blinded to technicians conducting analysis and data entry. Before assaying blood samples, the order of the blood samples will be checked against the database and samples are run in order of the database to provide efficient transcription and checking of results.

Data management, documentation and curation

Servers and data storage

A copy of all blinded anonymous (unidentifiable) qualitative, clinical, GIS and laboratory data will be stored on a remote University of British Columbia Server (PRE-EMPT shared drive with access control) located at the BC Children's Hospital Research Institute, which is locally and remotely backed up within the institute every day. When data are sent for merging with master databases, the research staff will also keep a copy of the file on their own network drive. Hard copies of results will be kept for at least 10 years. Data will be stored on secure database and file servers on a high-speed network with access control. Servers are stored in a secure locked server room with restricted access to authorised system administrators. The server room is protected by a security

keypad and monitored for environmental changes, availability and for any unapproved access. The PRE-EMPT Centre has extensive standard operating procedures along with research institute IT security policies.

All data will be stored locally on high capacity database and file servers. Servers are to be stored in locked rooms, with extra measures for security including auxiliary power through UPS and a diesel generator and system monitoring 24x7. A tape backup system is used for backing up the database.

Software platform management

REDCap is a web based secure application and runs on a PHP web server and MySQL database with at least 4GB RAM and 80GB hard drive space, which can be hosted on a Ubuntu LTS operating system or Windows system. Data entry forms will be created and customised for longitudinal programs on the software system. The built-in audit trails will provide historical records of data update activities.

OpenSpecimen is packaged as a standard Java Web Archive (WAR-file) and runs on any Servlet containers with Java Runtime Environment (JRE) version 8 installed, including the need of a web server and a database server with at least 4GB RAM and 80GB hard drive space. As the preferred software environment for production server, UbuntuLTS operating system, MySQL database and Tomcat Servlet are recommended.

Both REDCap and LIMS database systems will be hosted and managed by a local IT administrator and/or data manager in each country. The local IT administrator will be responsible for network security and access control, system updates, database backup and recovery and clients' computer updates. Local IT network, system and security policies and procedures will be followed. The data manager will be responsible for setting up and configuring the database, testing system functionalities, updating metadata and installing/configuring mobile apps on tablets for users. The data manager will also take charge of importing and exporting data, monitoring data updates, audit trails and reports and reporting/resolving data queries within the study team.

Consent process

Before a woman is enrolled in the PRECISE Network Study, she will have an opportunity to read or be read the informed consent in her native language and have an opportunity to ask the research coordinator any questions she may have. It will be explained that participation is voluntary and can be terminated at any time without reason and without any penalty. Consent will be confirmed with the participant's signature or a thumbprint. In the absence of a signature, a witness (other than the member of the research team obtaining consent) will be asked to sign on her behalf. Finally, the member of the research team obtaining consent will sign the form. All consent forms will be collected and stored at local sites in a locked cabinet or storage room accessible only to authorised staff.

Risks

Participation in the PRECISE Study does involve potential risks of a breach of confidentiality of personal information, medical records and associated privacy of the participants. Such risks will be minimised by: a) removing direct participant identifiers when not needed; b) limiting access to codes linking de-identified information with direct participant identifiers; and c) limiting access to information contained within the PRECISE databases.

Data quality control and data query

To ensure high quality of data, program rules will be added to implement skip logics and cross validation rules will be created for checking inconsistencies in real time. All these skip and validation rules along with the data entry forms will be tested to confirm the functionality of the system is behaving as expected and acceptable before starting data collection at sites. Blank paper forms will

also be printed out and will be used as a backup in case no electronic system or tablets are available for data entry.

It is the duty of sites to ensure the data collected are complete and accurate and to run validation rules on the forms in time. Data will also be queried periodically by the local data manager to check timely data collection and synchronisation, missing information and discrepancies. Data will be transmitted to the UBC central database weekly. The UBC data management team will also run data queries and produce a query report with feedback. The data query report will focus on missing values without reasonable comments, out-of-range values and data inconsistencies with violating validation rules and will be sent to the UK Coordinating Centre and local data managers periodically for reviewing and resolving with the local team.

Metadata standards and data documentation

The KCL and UBC leadership will be responsible for developing a detailed data sharing-document during the first six months of the project for approval by the PIs and collaborators, which will document the analytical methods, description of the variables, sample coding and means of access to each of the databases described above. This will be made available at a time agreed by the study PIs (see data sharing).

Data preservation strategy and standards

Backups of the UBC central database will be put into fire safe vaults for a minimum 10 years' storage. If needed, data can be transferred to DVD disks for even longer storage.

Data security and confidentiality of potentially disclosive personal information

General protection

To ensure privacy, all interviews and consenting will take place in a private room at the local health facility. Hard copies of the study-related forms will be stored in a locked cabinet or a storage room under supervision of the principal investigator and/or an authorised study coordinator at each local site. Electronic records will be stored in password-protected computers and only approved study personnel will have access to this information. Only de-identified data will be sent to the UBC PRE-EMPT Centre through a secure connection by the local data manager.

Database access control

All participants are given a unique study identifier. Personal information will be collected and held on a local database kept in a locked environment. Direct access to the database servers will be restricted to authorised individuals who are responsible for monitoring and backing up the system and databases. All other access to the database will be controlled by logical security with access control and occur across a standard secure (HTTPS) network protected by a firewall and system controls with authentication, role-based authorisation and audit trails. Strong password policies including the password length and complexity restrictions will be enforced. All database backups will be encrypted and local IT security policies and procedures will be followed.

Tablets security control

The tablets used to collect data will be protected and encrypted with an unlock passcode. Each user will need to login with a username and password to use the mobile app. This will ensure the study participant's confidentiality from the loss of tablets. The data collected on the tablets will be synchronised with the local server via a secure connection, preferably daily. To ensure confidentiality, only authorised staff (e.g. local PI/study coordinator, data collection supervisor or data manager) within the study team in each country can have access to personal information to facilitate data collection or data cleaning.

If any lost data occurs due to the loss or damage of a tablet, the last user who held the lost or damaged tablet shall report to the supervisor and/or data manager and study coordinator

immediately. The data manager or IT administrator will take the damaged tablet to erase or destroy information before recycling. The study coordinator will coordinate with the data manager to decide if another tablet needs to be released to the user.

Cautious use of computers and removable media devices

To mitigate the risks of potential disclosure of personal information, only the password-protected computers running an antivirus program will be used to download and process personal information for data linkage or data cleaning. Such files must be encrypted with a password (e.g. 7-zip with AES-256 encryption) and stored in the local secure network drive with access control.

If the data have to be stored temporarily on removable media devices like a USB flash drive or external hard drive, approval for their use must be given by a local PI or study co-ordinator as a delegate and the data files must be encrypted with a strong password before saving there. The data files containing personal information must be erased from the removable media devices right after use. Each user is responsible for the appropriate use and security of data and for not allowing removable media devices, and the information stored on these devices, to be compromised in any way whilst in their care or under their control. Removable media devices that are no longer required, or have become damaged, must be disposed of securely to avoid data leakage.

Data sharing and access

Suitability for sharing

We propose to collect data that will be suitable for sharing with national and international scientists and once we have completed the aims and objectives of this proposal we would open the dataset up for collaborators to use within the ethical and research governance constraints of the study (any datasets released to collaborators will always be anonymised).

Governance of access

This will comply with MRC guidance on data sharing, https://mrc.ukri.org/research/policies-and-guidance-for-researchers/data-sharing/, and be governed by the eight principals of the Data Protection Act. Data will be dependently available, in view of the limits implied by consent. An access committee will review applications to use the study data. The data will be held by the PI and Co-PI's institutions as there is no appropriate national repository. The Data and Sample Access Committee will comprise the PI and Co-PIs and named collaborators. Applications will be considered from academic and, potentially, commercial bodies, all of which must be for bona fide research or for educational purposes. All interested parties will complete a project proforma detailing the rationale for the study and the information required. The forms will be reviewed to ensure compliance with administrative requirements, prior to consideration by the committee which will meet by teleconference once every two months, or more- or less-frequently depending on demand. Once agreed, and following any requested revision, the form will be signed off by the PI and, following the signing of a data sharing agreement, the data will be made available. A record of the process will be kept and made available for periodic review by an independent assessor. A log will be kept of all studies, each of which will have a unique identifier. All agreements will be subject to a time limit for uptake, and if not met, the agreement will be withdrawn, so that other scientists could use the data for a similar/the same study. Academic researchers will be requested to provide the necessary costs for data retrieval and transfer, with a surcharge to support administration of the Data and Sample Access Committee, pro rata for the amount of data requested. Commercial bodies will be charged a premium over the cost recovery rate to reflect the value of the intellectual asset.

The study team's exclusive use of the data

The data will only be made available for external open access when all the data relating to the four predefined hypotheses have been published.

Restrictions to sharing

These will be primarily defined by consent agreements but also if the Data and Sample Access committee has reservations about the quality or bona fide value of the research.

Regulation of responsibilities of the users

External users will be bound by data sharing agreements including specification of the dataset(s) to be prepared and released, the purposes for which data are released and the conditions under which the data may be used, particularly in relation to ethics committee approvals. The specific obligations and arrangements to maintain confidentiality and data security will be itemised. The agreement will address handling of intellectual property, publication, authorship, acknowledgement and whether data are provided on an "exclusive" or "non-exclusive" basis to the requester. It will include a requirement that research publications and other outputs based on the data are reported to the PIs and that the UKRI be acknowledged in outputs. The researchers will agree not to transfer data to another unauthorised third-party. The researchers will also be asked to make available a copy of data derived, and details of how the data were derived, after a reasonable delay allowing the researchers to publish their aims and objectives prior to those derived data becoming widely available to other scientists. Arrangements for secure data archiving will be made and the costs of data retrieval detailed.

Responsibilities

The study PI and the co-PIs will be responsible for data management.

Relevant policies on data sharing and security

- General Data Protection Regulation (GDPR)
  https://ico.org.uk/for-organisations/guide-to-the-general-data-protection-regulation-gdpr/
- International Organization for Standardization
  http://www.iso.org/iso/home.html
- King's College London Research Data Management Policy
  https://www.kcl.ac.uk/governancezone/Research/Research-Data-Management-Policy.aspx
- London School of Hygiene and Tropical Medicine Research Data Management Policy
  http://researchonline.lshtm.ac.uk/612422/
- St George's, University of London Data Management Policy
  http://www.sgul.ac.uk/images/about/Policies/SGUL_RDM_Policy_May_2016.pdf
- The UK Data Protection Act 2018
  https://www.gov.uk/data-protection/the-data-protection-act
- University of Oxford Policy on the Management of Research Data and Records
  http://researchdata.ox.ac.uk/university-of-oxford-policy-on-the-management-of-research-data-and-records/

N. Ethics

The PRECISE programme of work requires ethical approval to cover a range of research questions relating to the placental diseases of pregnancy in the study settings. This will include qualitative, feasibility and usability studies in the areas of gestational age measurement, the broad socio-cultural context of pregnancy and childbirth, maternal mental health care and health systems provision for ANC and delivery services.

PRECISE is an observational programme of work. In these settings, there is an absence of clinical pathways to respond to identified clinical conditions, even a condition as common as gestational diabetes. As a consortium it is our considered opinion that it is unethical to make real-time diagnoses for which there are no available clinical responses. However, secondary batch testing of samples will delineate the burden of certain conditions and act as advocacy tools for future funding prioritisation, both within the health systems and research. In addition, once we have determined

the capacity of health systems to respond to demand, we will initiate long term health interventions (e.g. diabetes screening for women who meet criteria for gestational diabetes as their risk for type II diabetes is about 30% within 5 years) through the existing health systems.

The biological samples collected through the PRECISE study will be owned and permanently stored by the country from which they were collected. Our default position is that all laboratory science will occur in-country, as part of our research enterprise strengthening mandate. In instances where the samples are required for research purposes, and the equipment required for this research is unavailable in the country in which they were collected, these samples, with ethical approval and required MTAs, will be transported to the country where the necessary equipment to conduct the research can be found. Again, our default position will be to access laboratories within sub-Saharan Africa, as part of our remit to strengthen in-continent research capacity; this is a model already adopted by H3Africa, the human biorepository consortium created by the African Union. The consent forms that will be signed by women who have agreed to be in the PRECISE cohorts, describe this in the following way: *I agree to the use of the biological samples (blood, urine, vaginal swabs, cord blood and placental and cord tissue) in future ethically approved studies, only if the PRECISE Network Biorepository Data and Sample Committee approves the transfer of the samples, to other research collaborators/institutions in Africa or elsewhere in the world.*

PRECISE will be overseen by a Project Executive Group of the PI, site PIs and Co-PIs (2 per country), and theme leads and the PRECISE Programme Manager. Strategic oversight will be provided by the independent Technical Advisory Group. We will undertake ethically-approved human subject research in collaboration with the MRC Unit The Gambia at LSHTM (MRCG) and established collaborations in Kenya and Mozambique. (See Appendix 8 PRECISE Governance Framework).

Specific consent will be requested each for participation in the formative research underpinning this application, the associated discovery science and for studies requiring DNA and long-term sample storage in the biorepository. Consent will include 'pregnancy complications' in the broadest context, explicit acknowledgment of international collaboration and the possibility of moving samples between countries and possible collaboration with industry partners chosen for their overt and substantive dedication to global health. (Appendix 9 PRECISE Information sheet and consent form).

Eligible pregnant and non-pregnant women will be identified in primary health centres and the pregnant women followed through accurately-dated gestation through delivery and the puerperium. Including samples from non-pregnant women will aid understanding of the physiological adaptations to pregnancy in Africa. Anonymised demographic and clinical data will be entered into a study-specific, internet-based database; similarly-logged samples will be stored at local biorepositories.

The personnel who will take blood samples are well trained (e.g. phlebotomist, midwife or research technician). The only risk is minor discomfort during venepuncture but there is no risk to the mother or baby. Collecting mid vaginal swabs may cause mild personal discomfort and, therefore, may be declined by some women, but does not pose any intrinsic risk to the pregnant woman or her unborn baby. Sampling of urine, cord blood and placental tissues does not pose any risk.

Participation in the PRECISE Study does involve potential risks of a breach of confidentiality of medical record, self-reported, or genetic information and associated privacy of the participants. Such risks will be minimised by removing direct patient identifiers from the analytic dataset, and retaining these only in the strictly controlled site-led documentation, identical to the regular healthcare documentation.

For qualitative research, participants in FGDs and IDIs will be identified locally and sign informed consent prior to participation. Community engagement session participation will confer implied consent.

Scientific community access to the research infrastructure, data and samples will be encouraged and overseen by a Data and Sample Committee comprising PIs and external experts, according to the MRC data sharing policy. All staff will be trained in Good Clinical and Laboratory Practice.

O. Timeline

Below is a general project timeline. Each country has developed its own internal Gantt chart.

Activity	Y1 (Oct 17 -Sept 18)	Y2 (Oct 18 -Sept 19)	Y3 (Oct 19 -Sept 20)	Y4 (Oct 20 -Sept 21)
Contracts/ethics/SOPs				
Training				
Database management				
PPI: FGDs & IDIs				
TraCer app development				
Health Geography				
Clinical activity				
Basic science				
Analyses/reports/papers				

Figure 12 Overview Gantt chart for PRECISE activity

P. Research themes

Environment (Working Group: Makanga, Woodd, Barratt, Beevers, Filippi, Sandall, Boene, Vidler, Martinez-Alvarez, Blencowe, von Dadelszen, Strang, Flint-O'Kane)

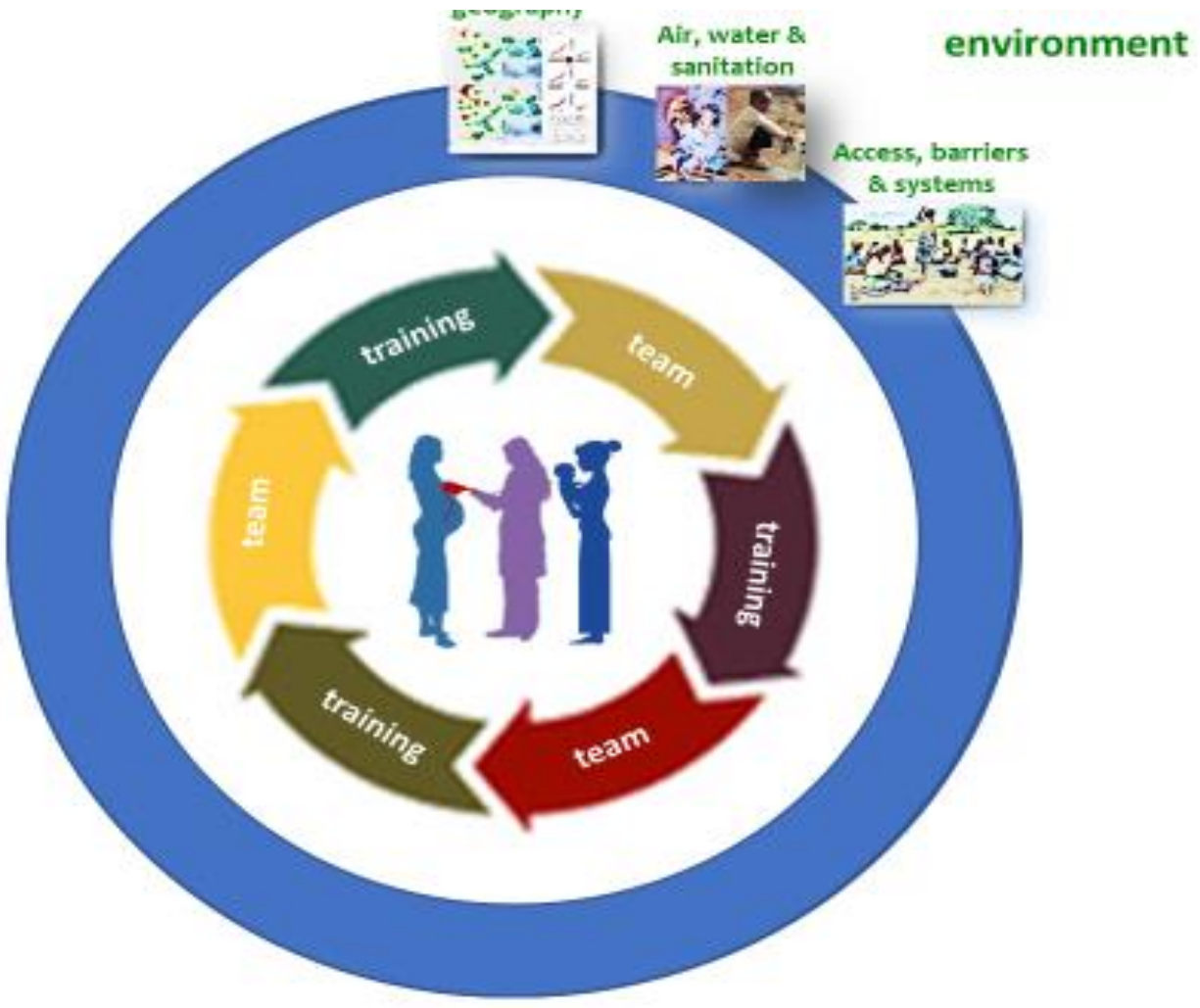

Health geography (Lead: Makanga)

We have developed a 9-step guideline for gathering and creating framework geographical information system (GIS) data in a data-poor setting [40]. Using this approach, we conducted a facility assessment in Maputo and Gaza provinces, Mozambique, to classify 56 health centres. GPS co-ordinates of the health facilities were acquired from the Ministry of Health while roads were digitised and classified from high-resolution satellite images. Data related to the geographic distribution of populations of women of reproductive age, pregnancies and births and transport options available to pregnant women were collected by household census. Daily precipitation and flood data were used to model the impact of severe weather on access for a 17-month epoch. Travel times to the nearest health facilities were calculated using the closest facility tool in ArcGIS software.

46% and 87% of pregnant women lived within 1h of the nearest primary care centre by walking or public transport, respectively. The populations within these catchments dropped by 9% and 5% respectively at the peak of the wet season. Similarly, the population of women within 2h of life-saving care dropped by 9% for secondary facilities and 18% for tertiary facilities during the wet season (see lower image on right for example of data visualisation).

Therefore, seasonal variation in maternal care access should not be viewed through a dichotomous, static lens of wet and dry seasons, as access continually fluctuates. This spatio-temporal access modelling approach, which will be adopted in the proposed programme in sub-Saharan Africa, permits GIS output use for both health services planning and near real-time community-level delivery of health services.

To examine interactions between time, place, infectious risk, food security and placental disorders, we will integrate real-time and location-specific health geography to estimate community- and individual-level resilience to placental disorders using the innovative methods described above to map new areas in Mozambique, the Gambia and Kenya.

We have identified the following specific hypothesis that we will test in this portion of PRECISE:

- That PRECISE will enlighten the geographical influences on the occurrence and outcomes of placental disorders of pregnancy.

We will map the different geographies of the PRECISE study areas, in consultation with the in-country mapping agencies. New data that describe the factors related to adverse maternal outcomes will also be mapped through household surveys. New methods for mass production of framework geospatial data will be explored through graduate projects.

In addition, much of the mapped data will be used for analysing access to maternal care and the spatial epidemiology of maternal and perinatal health. Evidence from these processes will be used to design m-health tools that will be used for targeted evidence-based action to improve maternal and perinatal outcomes.

A summary of the approach to be used in the portion of the project is appended (Appendix 10.

Environmental sciences (air quality, water, sanitation & hygiene) (Leads: Barratt, Beevers, Woodd)

Air quality: We will complement our health geography, nutrition and social science foci within PRECISE with expertise in air pollution personal exposure assessment, using a combination of small personal sensors, satellite aerosol optical depth data and outdoor air quality models as well as individual level human exposure models [41-43].

The specific hypotheses that we will test in this portion of PRECISE are being discussed by the relevant co-investigators and will be added to the protocol once agreed.

In more-developed countries there is an increasing body of evidence linking air quality with adverse pregnancy outcomes, such as placental complications, whilst in London traffic related air pollutants have been associated with babies of low birth weight and being small-for-gestational age.

In much of Africa, women are exposed to considerable levels of air pollution due to indoor fires and cooking stoves and this is compounded by poor outdoor air quality, especially in the growing African cities. For example, in Dakar, average PM2.5 and PM10 concentrations in 2010 were a factor of 2.5 and 7 times that in London, respectively; in a not dissimilar setting, it is known that asthma is the leading cause of maternal death in Karachi, Pakistan.

A summary of the approach to be used in the portion of the project is in development and will be available in due course.

Water, sanitation and hygiene (WASH): Due to the multiple pathways through which poor access to water, sanitation and hygiene (WASH) may have adverse impacts on maternal and newborn health (MNH) outcomes, PRECISE will assess the contributions of WASH within the complexity of the project. Due to their interdependent nature, these three core issues (i.e. water, sanitation and hygiene) are grouped together to represent a growing research and implementation sector; their interdependence is illustrated by the facts that, without toilets, water sources become contaminated; without clean water, basic hygiene practices are impossible [44].

The specific hypotheses that we will test in the WASH portion of PRECISE are being discussed by the relevant co-investigators and will be added to the protocol once agreed.

We will put the required elements in place by:

- A combination of remote communication, discussion with PRECISE colleagues and visits to the countries to establish what air quality measurements, traffic and other emissions and WASH source data exist and plan for further data collection if necessary
- To establish the level of housing information available, including indoor sources of air pollution, fuel used and WASH facilities
- The availability of routinely collected health data, its suitability for future epidemiological analysis and to plan for further data collection, if necessary
- To develop an analysis plan whereby outdoor and indoor personal exposure estimates and WASH data can be linked with health data for future analysis
- To begin feeding these data into the PRECISE database, as part of a 'beta' test of the system and to adjust the analysis plan where necessary

A summary of the approach to be used in the portion of the project is in development and will be available in due course.

Access, barriers and systems of care (Leads: Sandall, Filippi, Vidler, Boene, Martinez-Alvarez, Flint-O'Kane, Daniele)

There are important factors preventing timely care-seeking and effective system response for obstetric emergencies and delivery. Delay due to unfamiliarity with warning signs, especially among family members, discouragement from revealing pregnancy early in gestation, gender inequalities, complex and untimely decision-making processes, fear of mistreatment by health-care providers, lack of recognition by frontline staff and delay in escalation within the health system, lack of transport and financial constraints were the most commonly cited barriers. In rural Mozambique, we determined that women do seek antenatal care at health facilities, despite other health care providers being in the community.

We will draw on social and implementation sciences to explore enabling and impeding factors for pregnant women, with and without pregnancy complications at all levels of a woman's journey through care, including assessment of health system responsiveness. We will use ethnographic and mixed methods research across all sites using a realist evaluation approach to develop explanations that address the questions about how complex programmes, 'what works, for whom, under what circumstances and how', providing insights into implementation contexts. This will include investigation of the contribution of context and sociocultural factors, quality of care received and concepts of respectful maternity care in the PRECISE settings.

Socio-cultural qualitative work (Leads: Boene, Vidler)

The socio-cultural qualitative component of PRECISE is led by Helena Boene (Centro de Investigação em Saúde de Manhiça) and Marianne Vidler (University of British Columbia). This mixed methods work will be guided by the ecological framework, positing that no one factor can account for pregnancy outcomes and builds on the CLIP experience [3-6,45-61]. One must consider how factors at

the individual, interpersonal, community and societal level influence the health of women in pregnancy and postpartum.

Objectives:

1. To understand the socio-cultural context of women in PRECISE, particularly as this may influence placental disorders in pregnancy.
2. To evaluate the perceptions (acceptability) of a biobank of maternal and infant specimens (taken during pregnancy and postpartum)
3. To provide knowledge and understanding of the socio-cultural context that will inform the development of future intervention studies for the prevention, diagnosis and treatment of placental disorders in these contexts.

Further detail of the planned activity in this work stream can be seen in Appendix 11.

Quality of care (Leads: Filippi, Flint-O'Kane, Daniele)

With women accessing maternity services more than ever before in African settings, the quality of care they receive in health facilities and how it can be improved has become an important research and programmatic issue.

Quality of care is care that is effective, efficient, acceptable to patients, equitable and safe (IOM definition). Quality of care measurement is often done using a simple framework of indicators of input, processes and outcomes, on the initial and very popular Donabedian definition of quality of care. WHO has recently published a conceptual framework for quality of care inspired by the IOM definition and the Hulton framework Figure 12:

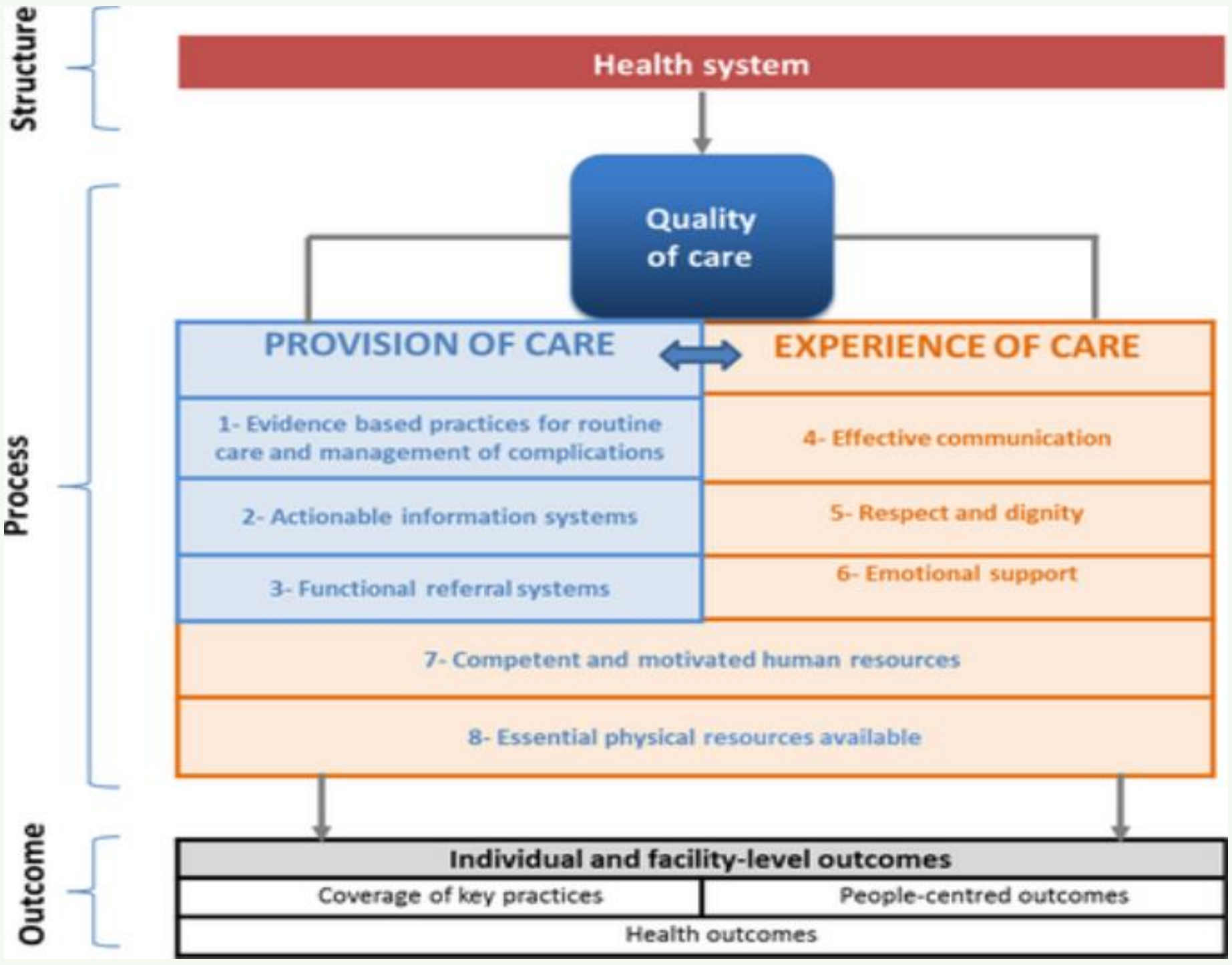

Figure 13: Hulton framework

Problem identification:

Little is known about the quality of the services provided to women in Mozambique, Kenya and The Gambia, generally and most specifically as relates to placental disorders (hypertensive disorders, fetal growth restriction and stillbirth).

Objectives:

- To prepare a conceptual framework of quality of care for the prevention and treatment of placental disorders on the basis of the literature and consultations with clinical experts and other stakeholders
- To apply the conceptual framework to document the quality of care for women with placental disorders using a mixed method approach, focussing on indicators of inputs and processes, in all facilities that take part in the PRECISE cohort – some of these indicators will be collected at the level of facilities (inputs) and some at the level of women (processes)
- To link data on some key inputs and processes findings with the health outcomes measured by the epidemiological stream, to obtain a complete picture of quality of care
- To quantify the relationship between quality of care risk factors and placental disorders (if we can build a sufficient sample)
- To use these data to provide a rich description of the context in which adverse outcomes are occurring and generate suggestions for clinical and policy interventions

Methods:

Study design: mixed methods cross-sectional study

Activities:

- Phase 1: development of conceptual framework – literature review, key informant interviews, workshop
- Phase 2 - implementing the framework - data collection. Sampling: could follow some women who are identified at first ANC as at risk of PD or who have particular RF

Inputs will be measured through one or more visits in health facilities to measure signal functions in relation to placental disorders (i.e. can they provide the care)

Processes will be measured by interviewing women and by observing care (i.e. are they providing the care and how (is it safe, etc)). Interviews with women will be in-depth using an unstructured discussion guide during the antenatal care period and postnatally. A structured questionnaire for quantitative analysis might be used for a larger sample, budget permitting, and if the epidemiology stream allows us to add a small number of questions.

Respectful care (Lead: Sandall, Daniele)

A central component of global efforts to reduce maternal mortality is to ensure that all women have access to skilled care before, during and after childbirth. Access to quality services is not guaranteed for many women, however, especially in low- and middle-income countries (LMICs). Even when services are available, care may be compromised by mistreatment during childbirth, including abusive, neglectful, or disrespectful care.

Respectful care is emerging as a phenomenon of study since the World Health Organization (WHO) conceptualisation was published in 2015. The White Ribbon Alliance operationalised the conclusions of the WHO into seven domains or standards of disrespectful care in the Charter of Universal Rights of Childbearing Women including the WHO conceptualisation in 2015 and a Lancet special issue in 2016. However, what constitutes RMC operationally (in terms of specific behaviours, practices or standards) in research and programme implementation is often variable.

An overview of evidence and research gaps in this area of work is provided in Appendix 12. *The specific hypotheses that we will test in this theme within PRECISE are being discussed by the relevant co-investigators and will be added to the protocol once agreed*

Health systems (Leads: Martinez-Alvarez)

Introduction

Women and babies suffering from placental disorders need access to quality, efficient and equitable health services during pregnancy, labour and the postnatal period. This is dependent on a well-functioning health system, with efficient and equitable financing, adequately trained and motivated human resources, effective governance and accountability structures, functioning referral pathways and adequate physical resources and information systems [62].

Aim

The aim of this study is to assess and compare the health systems of The Gambia, Kenya, Mozambique and Senegal to draw lessons on how services can best be provided to women and babies with placental disorders.

Objectives:

- To assess and compare access to and use of health services along the continuum of care for women and their babies across countries
- To take a people-centred approach to assess the different components of the health system in the four countries and, where possible, link these to maternal and newborn health outcomes
- To draw recommendations for countries in this study and beyond on how to improve the health system for mothers and their babies

Conceptual framework

The study will be embedded in the de Savigny and Adam's systems-thinking framework [63] (Figure 13). Originally defined by the WHO, this framework recognises that the six health systems building blocks are interconnected, dynamic and people-centred.

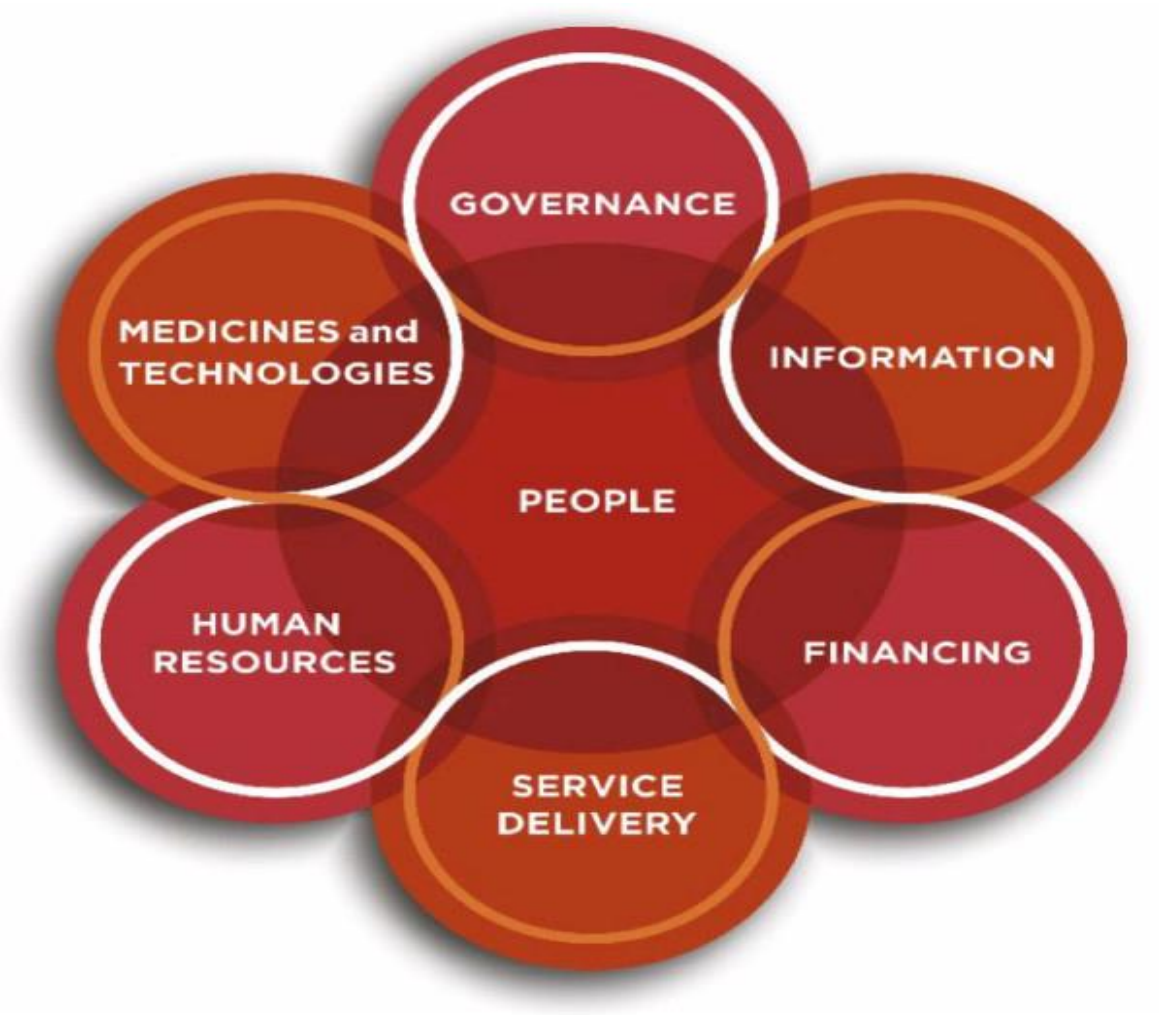

Figure 14. Health systems framework (de Savigny and Adam, 2009[63])

Methods

We will use a mixed-methods approach to assess the health systems of The Gambia, Kenya, Mozambique and Senegal. Senegal has been added to the PRECISE countries to complement The Gambian study and increase the applicability of findings across West Africa. Data collection in Senegal will take place in the Kaolack region, which is located directly across the border from The Gambian study site (Farafenni). Data collected from Senegal will not be analysed in conjunction with outcome data provided by the PRECISE cohort.

The health system component of PRECISE will have two strategies:

(i) If possible, a small number of questions will be added to the Epidemiology and Geography surveys

(ii) New data will be collected through facility-based surveys, document reviews and semi-structured interviews. There will be two phases of work: formative research to develop and test research tools, followed by data collection and analysis. The sample of facilities in which the facility surveys will be administered may be extended to cover all facilities in each study site.

Service delivery, medicines and technologies

We will map out the health system configuration for maternal and newborn health in all countries, including identifying which facilities provide services for placental disorders, how these are linked through a referral pathway and how medical supply chains work in each country. We will assess the physical infrastructure, medicines and technologies available in these facilities, including provisions of basic and comprehensive emergency obstetric care and newborn care, water and sanitation conditions and drug stock-outs. We will calculate women's and babies' use of pre-pregnancy, antenatal, delivery, referral and postnatal services by maternal education and household income.

Human resources

We will assess the degree to which human resources that are competent, motivated and in sufficient numbers are available to provide women and their babies suffering from placental disorders with appropriate care. We will map out the pre-service and in-service training requirements for health workers that provide these services and compare these across the four countries. We will also assess the number of health workers by cadre and level of training for each facility and compare this against internationally agreed minimum standards. We will assess and compare health worker motivation across settings, adapting existing methodology [64]. Factors that will be included in health worker motivation analysis will include salary structure, performance management and clinical supervision. The degree to which analysis is descriptive or analytical will depend on the sample size achieved. The association between human resource numbers, training and motivation on maternal and newborn health outcomes will be assessed, as well as the minimum numbers of health workers necessary to provide safe services to women and babies suffering from placental disorders.

Financing

Financing will be analysed from the perspective of the health system (supply-side) and from the perspective of women and their babies (demand-side). To examine how the health system is financed we will assess the income available by facility by source (government funds, health insurance, out-of-pocket payments and external funds) and expenditure item (including salaries, facility running costs, drugs, infrastructure and equipment). Supply-side financing data will be analysed to compare total and per capita expenditure, to test associations between financing and maternal and newborn outcomes, to assess the efficiency, equity and the incentives arising from differing financing arrangements. Demand-side financing will be assessed to explore the burden on households of using services for placental disorders and to examine progress towards achieving universal coverage in each of the countries studied. Data will be collected on direct (medical and non-medical out-of-pocket) and indirect costs (loss of income) and household income. Data will be analysed to explore the impact of placental disorders on poverty (catastrophic and impoverishing expenditures) by household wealth and to assess equity of financing (by performing a financing incidence analysis if sample size allows)

Governance and information systems

We will review all policies relevant to placental disorders in each country, including clinical guidelines and policies specific to the different health systems components. In addition, we will

assess the information system of each country, including the types of data collected and completeness and timing of reporting. We will further explore downward and upward accountability systems available for women who have placental disorders.

Barriers to access

If the budget allows, the work undertaken by the socio-cultural and geography groups may be replicated in the Kaolack region of Senegal.

Biology (Working Group: Poston, Tribe, Omuse, Okiro, Wanyonyi, Chappell, Lawn, Whitley, Cartwright, Craik, Russell, von Dadelszen)

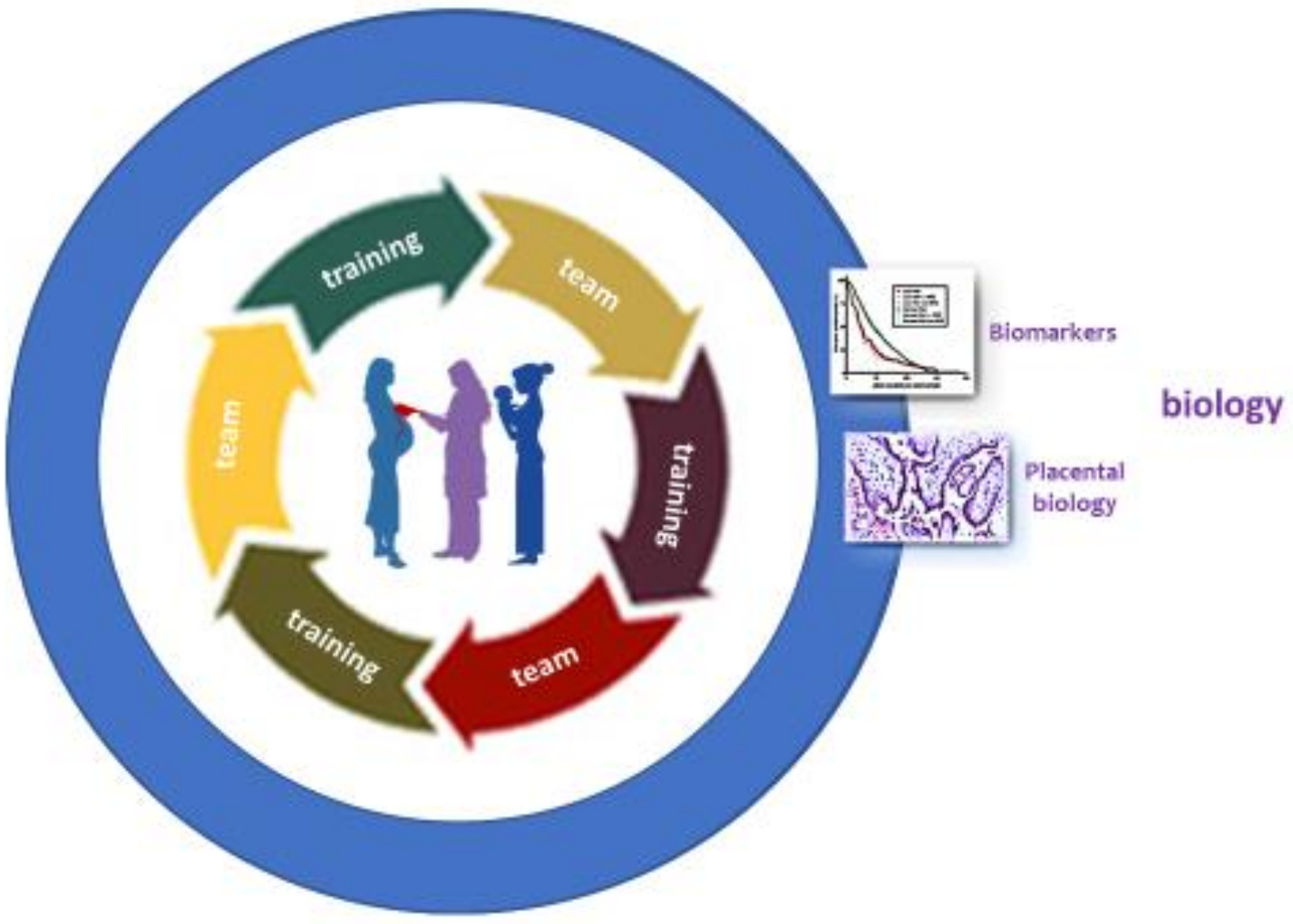

Biomarkers (Leads: Poston, , Chappell, Lawn, Tribe)

Pre-eclampsia, FGR and PlGF in more- and less-developed countries [65-70]:

The diagnosis of pre-eclampsia by BP and proteinuria is of limited use as these are tertiary, downstream features of the disease. Similarly, discriminating between placentally-mediated FGR and constitutionally-small fetuses is a challenge in obstetric practice. PlGF is an angiogenic factor, a secondary marker of associated placental dysfunction of pre-eclampsia, with known low plasma concentrations in the disease. In the UK, women presenting with suspected pre-eclampsia at 20^{+0}-34^{+6} weeks' gestation, low maternal plasma PlGF concentration (<5th centile for gestation, Quidel Triage® assay) identified women who delivered with confirmed pre-eclampsia within 14 days (sensitivity 0.96 [95% CI 0.89–0.99], negative predictive value (NPV) 0.98 [95% CI 0.93–0.995]). In addition, in UK, Canadian and NZ women with suspected FGR, low PlGF identified placental FGR with an AUC ROC 0.96 [95% CI 0.93-0.98], sensitivity 0.98 [95% CI 0.91-0.999], specificity 0.75 [95% CI 0.68-0.82] specificity, NPV 0.99 [95% CI 0.95-0.999] and PPV 0.59 [95% CI 0.48-0.69]26. Low PlGF outperformed other variables in predicting placental FGR. Very low PlGF (<12 pg/mL) was associated with shorter sampling-to-delivery intervals than normal PlGF (13 vs. 29.5 days, p < 0.0001). In a less-developed country, we studied maternal plasma PlGF in women with suspected pre-eclampsia attending two antenatal clinics in Maputo, Mozambique. The clinic-to-delivery interval was shorter in low PlGF, compared with normal PlGF, women (median 24d [IQR 10-49] vs 44d [24-81], p=0.0042). Also, low PlGF was associated with a confirmed diagnosis of pre-eclampsia, higher blood pressure, transfer for higher care, earlier gestational age delivery, delivery within 7 days and 14 days, preterm birth, Caesarean delivery, lower birth weight and perinatal loss.

PlGF & stillbirth:

Unpublished pilot data from PRE-EMPT's Global Pregnancy Collaboration show that in 45 women destined to suffer a stillbirth, the gestational age-corrected median plasma PlGF was 0.012 multiples of the median compared with >1000 normal pregnancies (p=0.004).

In addition, in recently-published data from Maputo, one-third of women with both pre-eclampsia and PlGF <50pg/ml suffered a stillbirth [68]. In PRECISE, we will not only utilise PlGF, but source additional funding to carry out an 'unsupervised' biomarker programme to develop new tools for prediction, diagnosis and management with potential for point-of-care testing. The existence of these cohorts will greatly facilitate the sourcing of leveraged funding from UK and global partners.

PRECISE will confirm or refute our *a priori* hypothesis that screening pregnant women for placental health can identify women at risk of stillbirth. PlGF is our initial biomarker of interest, using a random 20% of PHC samples and all time-of-disease samples, PRECISE will also aim to improve the performance of the PIERS models with biomarkers, starting with PlGF.

The planned PRECISE biorepository affords an opportunity to identify and test other time-of-disease biomarkers for potential in stratifying individual women's risks. For example, accurate diagnosis of pre-eclampsia is difficult in women with chronic hypertension, a common co-morbidity in pregnant Black African women.

Placental biology (Leads: Whitley & Cartwright)

Maternal-fetal-placental immunology

During normal pregnancy, the placental trophoblast invades the maternal uterine spiral arteries, reducing resistance to flow and establishing the placental blood supply. In early-onset pre-eclampsia and placental FGR partial failure of this process leads to reduced placental perfusion.

Complex interactions between the maternal immune system and paternal antigens, her uterine vascular biology and the receptivity of the decidua and the trophoblast have all been implicated. Decidual natural killer cells, macrophages, placental endothelial cells, trophoblast and stromal cells in first trimester pregnancies at increased risk of developing pre-eclampsia and FGR reveal differences which offer some mechanistic insight. In less-developed country settings the immunomodulation of successful pregnancy is likely to be compounded by the presence of either food insecurity or infectious agents or both. PRECISE scientists will have a unique opportunity to address mechanisms in an appropriate setting.

The specific hypotheses that we will test in this portion of PRECISE are being discussed by the relevant co-investigators and will be added to the protocol once agreed.

Placental Biology

Gene expression at term in normal pregnancies and those complicated by pre-eclampsia will provide limited insight in to the aetiology of the syndrome but will provide useful data regarding differences between early and late onset disease. However, this region is faced with further challenges associated with bacterial, viral and parasitic infections. Understanding the interaction of these infections with both a normal and pre-eclamptic pregnancy may be of immense value in targeting treatments to individuals or groups of individuals. It is anticipated that with the limited funds available analysis of only small numbers of individuals per group is likely. We would suggest using very tightly matched groups in an effort to obtain preliminary data for future applications.

Gene Array studies

Isolation of RNA should be compatible with analysis using RNA-Seq and would be best achieved using a service provider. Costs approximately £300 per patient for preparation and sequencing. Number of samples per group minimum of 8.

1. Initial comparison normal vs PE early and late onset
2. Groups need to be well matched for the usual, mothers age, parity, gestational age (late onset), BMI, etc. Also, should start by excluding known medical problems such as infection. In the light of current thinking comparisons based on the sex of the child should be considered
3. Should also consider comparisons between regions of the placenta according to SOP
4. Should consider some sample preparation following isolation prior to banking samples
5. Validation of the array data by western blot and RT-PCR of an independent cohort

Protein expression, immunohistochemistry

Identification of target genes will direct future immuno-histochemical studies. Examination for gross structural changes particularly between infected and non-infected placental tissue should be possible. The most significant hits identified from the array could be examined to identify differences in distribution or expression patterns. In these studies, we will have to be mindful of exactly where the samples are derived i.e. central or peripheral.

6. Costs to:
   a. fix and embed samples approximately £10/sample
   b. Section and basic histological staining £75 for 30 sections/sample
   c. Immunohistochemistry dependent on number of antibodies and cost per antibody
   d. Slide scanning £20/h 15 slides/h

Future comparisons

1. Blood samples for more targeted studies as performed on the Ugandan population looking specifically at typed for *KIR* and *HLA-C* variants including for presence/absence of *KIR2DS5* alleles (Am J Hum Genet. 2016 Aug 4;99(2):375-91)
2. Epigenetic studies i.e. methylation patterns in placental tissue
3. Comparison with first generation migrants
4. Healthy vs individuals with parasitic infection such as malaria
5. Healthy vs sickle cell

Epidemiology & co-exposures (Working Group: Magee, Moore, Krishna, D'Alessandro, Macete, Filippi, Sevene, Salisbury)

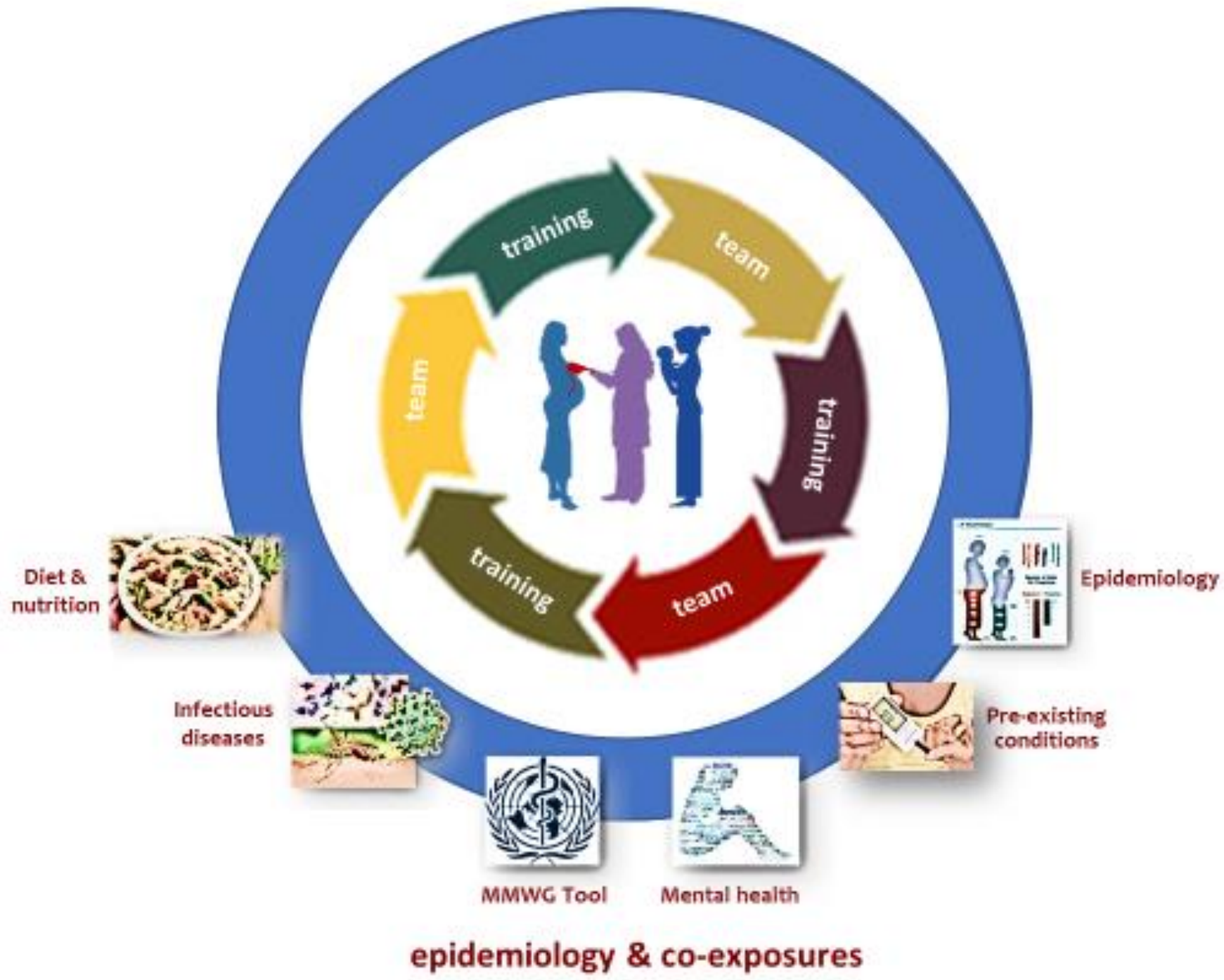

Epidemiology (Lead: Magee)

Interventions cannot be implemented without accurate assessment of the prevalence of placental disorders in the different centres/countries. To determine the prevalence of placental disorders and neonatal outcomes, we need to identify population-representative samples of pregnant women, date their pregnancies and follow them and their infants into the postnatal period (ideally to the end of the puerperium at 6 weeks). This will complement ongoing WHO activity in The Gambia, assessing and enumerating pregnancy outcomes.

We have identified the following specific hypotheses that we will test in this portion of PRECISE:

- That the accurately-dated and well-characterised PRECISE cohort will enable population-level estimates of the incidence of placental disorders of pregnancy, preterm birth and hyperglycaemia in pregnancy.
- That the well-characterised PRECISE cohort will enable population-level estimates of the prevalence of non-communicable disorders in pregnant women and non-pregnant women of reproductive age.
- That the well-characterised PRECISE cohort will enable population-level estimates of the prevalence of mental health disorders in pregnant women and non-pregnant women of reproductive age.
- That the well-characterised PRECISE cohort will enable population-level estimates of the prevalence of infectious diseases in pregnant women and non-pregnant women of reproductive age.

We will conduct longitudinal studies of women with identified pregnancies in primary health centres (PHCs) and district hospitals (DHs) in each country. Each cohort will recruit for 24 months. The women will be approached to provide the biological samples described below. In addition to routine data collection related to social determinants of health, demographics, clinical findings (symptoms, signs, and laboratory tests) and outcomes (through PHC and facility surveillance) and the new WHO

Maternal Morbidity Tool, WOICE, we have embedded two sub-projects into the project: TraCer and CRADLE (see below).

Pre-existing conditions (non-communicable physical diseases) (Lead: Magee)

Many sub-Saharan African women live with pre-existing medical conditions, some of which are heritable (e.g. sickle cell disease) and others which are acquired (e.g. rheumatic heart disease). These maternal medical conditions will confound the relationship between pregnancy and health. It is for that reason that women with these conditions will not be excluded from PRECISE.

In addition, women who have had a pregnancy complicated by either any one, or a combination, of the placental disorders, identify themselves to be at increased risk for premature cardiovascular disease (hypertension, myocardial infarction and stroke) and cardiovascular death. Furthermore, the placental disorders identify women at increased risk for type II diabetes mellitus.

We have identified the following specific hypotheses that we will test in this portion of PRECISE:

- That PRECISE will identify the independent contribution of physical NCDs to the occurrence of pregnancy complications.

Our plan is to use biomarker-based research to identify the burden of NCDs in these populations of reproductive age women. In addition, we will seek funding to screen populations for congenital and acquired cardiac disease, especially following pregnancies complicated by placental disorders.

Maternal mental health (Lead: Salisbury)

It is important to determine how maternal mental health influences pregnancy and pregnancy outcomes in women in sub-Saharan Africa. In more-developed countries, it is known that maternal stress, anxiety and depression are associated with an excess risk of preterm delivery. Also, it is recognised that women who have complex pregnancies, often living with perinatal uncertainty for days or even months, have increased risks for postnatal depression and psychosis. Those who endure operative vaginal (e.g. forceps, breech) or Caesarean deliveries are at increased risk of suffering from post-traumatic stress disorder (PTSD) and through our work with the WHO, assessing the Maternal Morbidity Working Group tool, WOICE, we have determined that African women list anxiety, depression and intimate partner violence as their dominant morbidities.

Therefore, we have complemented our initial social science and physical health foci within PRECISE by adding expertise in mental health assessment, using standardised data collection tools, some of which are embedded in WOICE, as well as a tool for PTSD.

We have leveraged additional funds from the Bill & Melinda Gates Foundation Grand Challenges Explorations platform to fund a particular focus on adolescent maternal mental health, the Catalyst project (currently limited to Mozambique; more leveraged funding is being sought for The Gambia and Kenya).

We have identified the following specific hypothesis that we will test in the Catalyst portion of PRECISE:

- That, in Catalyst, engaging and empowering young mothers in the development of methods to deliver mental health interventions is feasible and will result in greater acceptability of interventions to improve mental health outcomes.

In Catalyst, a mixed method design will be used to develop and evaluate an intervention(s) to improve wellbeing among young women in the perinatal period over 18 months. A human-centred approach will be used to understand the conceptualisation of and priorities for wellbeing and mental ill health (Phase 1), identify a priority challenge(s) to wellbeing during the perinatal period (Phase 2), select and adapt existing mental health intervention(s) to address the challenge(s) and develop up to

three prototypes for implementation (Phase 3). These prototypes will be evaluated for fidelity, feasibility, acceptability and impact on clinical outcomes through a five-month pilot study using a before-and-after design, with up to 60 young mothers recruited from maternity clinics and community-based organisations to reach those both in receipt and not in receipt of medical care.

A detailed Catalyst protocol is appended (Appendix 15).

For more general mental health issues, we have identified the following specific hypothesis that we will test in the Catalyst portion of PRECISE:

- That maternal stress, anxiety and receipt of domestic violence will modify pathways to placental disorders and secondary outcomes.
- That pregnancy complications and the style of their management contribute to the burden of PTSD assessed at the end of the puerperium.

Other maternal mental health fields are assessed by WOICE, the MMWG tool, which is discussed in more detail, immediately below. During initial testing of the WOICE tool, up to 40% of pregnant women, uniformly of African origin, experienced significant levels of stress, anxiety and domestic violence.

WOICE: Maternal Morbidity Working Group tool (Leads: Filippi, Magee, von Dadelszen)

Convened by the WHO, the MMWG has agreed on the following definition of maternal morbidity: "any health condition attributed to and/or aggravated by pregnancy and childbirth that has a negative impact on the woman's wellbeing". This new definition of maternal morbidity will be proposed for inclusion in the 11th revision of the International statistical classification of diseases and related health problems. Professors Filippi, Magee and von Dadelszen are members of the MMWG.

The MMWG has developed a tool, WOICE, that can be used to assess maternal morbidity at scale. However, the tool has yet to be validated. The plan is to beta-test the tool in these three African geographies.

We have identified the following specific hypotheses that we will test in this portion of PRECISE:

- That the WOICE tool administered in the third trimester will be successfully beta-tested in this project.
- That the MMWG WOICE tool administered at approximately six weeks postpartum will be successfully beta-tested in this project.

We have embedded all elements of the WOICE tool into the PRECISE database fields for the follow-up visits during pregnancy and the end of the puerperium.

Perinatal & newborn health (Leads: Blencowe, Lawn)

The PRECISE Network will provide a platform to investigate important unanswered questions to improve understanding of epidemiology, burden and contributing underlying factors for stillbirth and adverse neonatal outcomes including preterm birth, fetal growth restriction, neonatal encephalopathy and infections.

Important questions include improving the understanding of:

- the interaction between non-communicable conditions, infection and intrapartum related deaths (stillbirths and intrapartum related early neonatal deaths).
- the contribution of post-term/preterm birth to perinatal mortality in high burden settings.
- the interaction between fetal growth disorders and length of gestation on mortality – including preterm small-for-gestational age, post-term small/ large-for-gestational age.

In addition, the PRECISE cohort with its well phenotyped maternal disease and fetal/neonatal outcomes will allow detailed analysis to improve the understanding of the contribution of underlying maternal, placental and fetal factors to disorders of fetal growth and length of gestation.

We have identified the following specific hypotheses that we will test in this portion of PRECISE:

1) Non-communicable conditions and infections are hypothesised to be important underlying contributing factors in cases of intrapartum death (both stillbirth and intrapartum related neonatal death) but the contribution has not been quantified in these settings.
   a. What is the interaction between non-communicable conditions, infection and intrapartum related deaths (stillbirths and intrapartum related early neonatal deaths) in these settings?
2) Disorders of fetal growth and length of gestation (preterm and post-term birth) are important contributors to perinatal mortality in high income settings. These are likely also to be important contributors in LMIC settings but poor characterisation of gestational age has limited the ability of previous studies to determine this contribution, especially for post-term births.
   a. What is the contribution of post-term/preterm birth to perinatal mortality in these settings?
   b. What is the interaction between fetal growth disorders and length of gestation on mortality – including preterm SGA, post-term SGA/LGA?
3) Maternal, placental and fetal factors all are hypothesised to potentially contribute to disorders of fetal growth and length of gestation but these have been inadequately characterised previously in these settings.
   a. What underlying maternal, placental and fetal factors contribute to disorders of fetal growth and length of gestation? (including GDM, hypertension, infections etc).

Infectious diseases (Leads: Krishna, D'Alessandro, Macete, Sevene)

Given the seasonality of pre-eclampsia (it is unclear whether or not FGR and stillbirth are similarly seasonal), it is important to examine the interaction between season, day-to-day fluctuations in geographical variants, seasonal dietary variation, infectious agents (viruses, bacteria and parasites) and placental disorders. For example, there is conflicting evidence related to the possible contribution of malaria infestation of the maternal vascular (intervillous) space of the placenta and all three placental disorders of interest – if so, such an effect may only be operative in first ongoing pregnancies. However, HIV infection may protect against pre-eclampsia.

Our team has considerable breadth and depth in infectious diseases expertise. This is relevant to viral, bacterial and parasitic infectious disease in pregnant and non-pregnant women and infants. We have established interests in the development of new diagnostic technologies for diseases such as malaria and tuberculosis, which will not only confirm disease but also guide the choice of effective treatment regimens.

We have identified the following specific hypotheses that we will test in this portion of PRECISE:

- That PRECISE will identify the independent contribution of infectious diseases to the occurrence of pregnancy complications.

We will examine women and their biological samples for evidence of infectious disease and map the complex interactions between time, place, nutrition and infectious agents to clarify these interactions in a definitive and novel manner. We will rely considerably on extant in-country expertise to lead these aspects of the research, with critical linkage through, and collaboration with, PRECISE.

Diet/nutrition (Lead: Moore)

Aggregated undernutrition, including fetal growth restriction, stunting, wasting, deficiencies of vitamin A and zinc and sub-optimal breastfeeding is estimated to be the cause of 3.1 million child deaths annually and of 45% of all child deaths in 2011. Given the focal role of the placenta in supply of fetal nutrients, a detailed understanding of how (i) maternal nutrition regulates placental function and (ii) placental regulation of nutrient supply influences fetal growth and FGR will be an important element of this initiative.

We have identified the following specific hypotheses that we will test in this portion of PRECISE:

1. Food insecurity and/or a low dietary diversity among pregnant women increases risk of placental disorders.
2. Maternal undernutrition (assessed by short stature, low BMI, low MUAC) increases risk of placental disorders among pregnant women.
3. Sub-optimal micronutrient status in pregnancy increases risk of placental disorders.

Maternal diet: Given the challenges of accurately capturing dietary/nutrient intakes within and between populations, for the current study we will develop population-relevant questionnaires to assess dietary diversity and food insecurity among participating women. These measures will provide a qualitative measure of food consumption, reflecting household access to a variety of foods and providing a proxy for nutrient adequacy of the diet of the individual women.

Nutritional biomarkers: Nutritional status will be assessed directly through the measurement of nutritional biomarkers, on the same random 20% of PHC samples and all time-of-disease samples. Biological samples (plasma and urine) will be used to measure micronutrient status, including iron, folate, iodine, and vitamins A, D and B12. Where possible, we will use multiplex approaches, limiting sample volume requirements and costs.

Clinical Innovation (Working Group: Shennan, Koech, Papageorghiou, Blencowe, Roca, Magee, von Dadelszen, Craik)

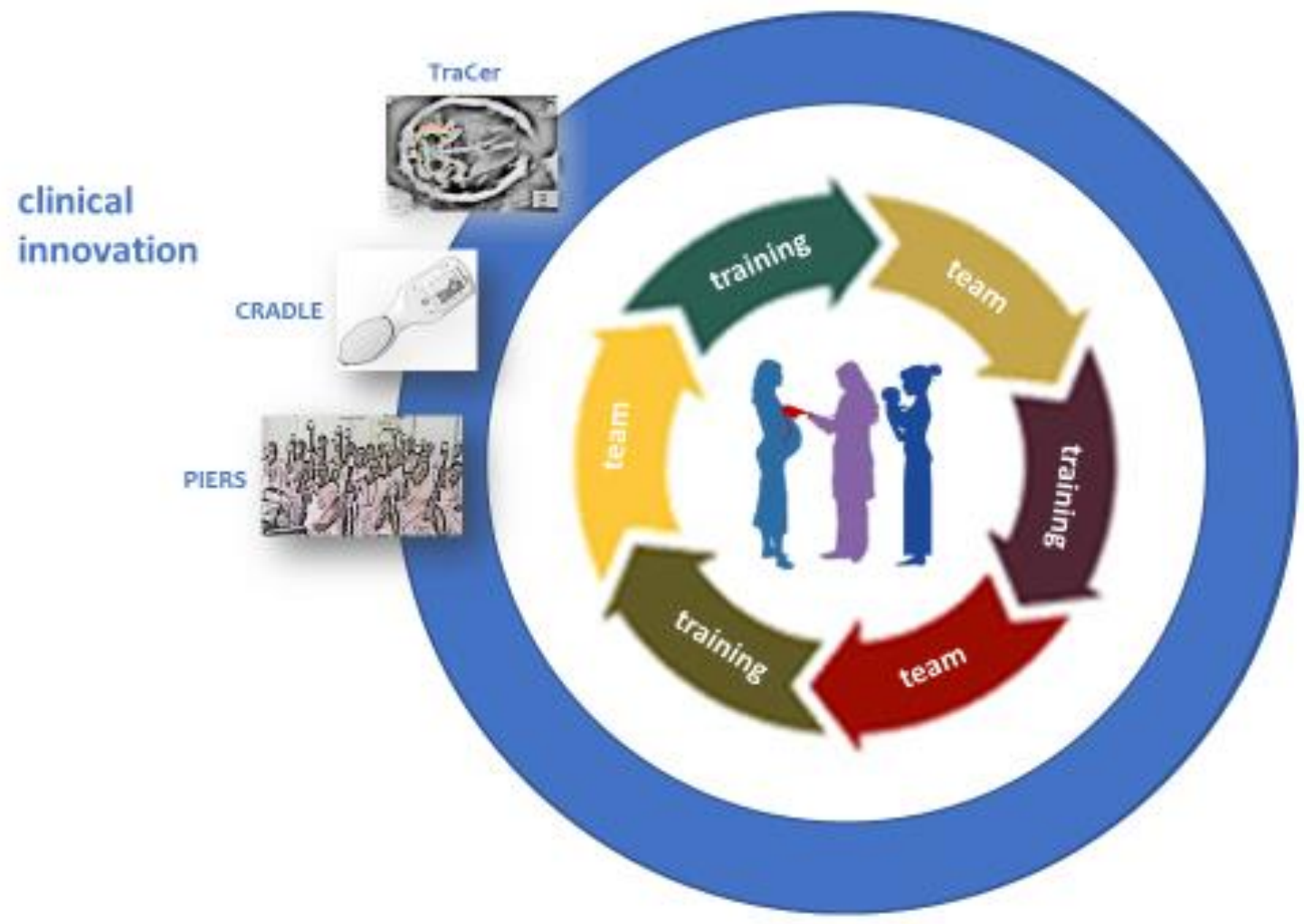

TraCer (Leads: Papageorghiou & Noble)

Accurate knowledge of gestational age (GA) is a cornerstone of modern maternity care, reducing unnecessary interventions while reducing stillbirths and other obstetric complications. In most LMICs, many women never receive antenatal care or, if they do, present at 20 weeks' gestation or later. Pregnancy dating using either maternal recall of her last menstrual period or symphysis-fundal

height is inaccurate. Although ultrasound is the most accurate approach, lack of expensive equipment and trained staff means accurate GA dating is often unavailable in LMICs despite its obvious need.

We have developed a mobile health (mHealth) and image analysis ultrasound platform for automated measurement of the fetal transcerebellar diameter (TCD); this measurement correlates with GA and, in the absence of fetal anomalies, is relatively protected from the influence of FGR and remains reliable throughout pregnancy from 14 weeks until term (37^{+0} weeks). This is important in settings where FGR is likely to be more common, which would lead to a systematic error in underestimating GA if the normal four biometric parameters (biparietal diameter, head circumference, abdominal circumference and femur length) are used. With both poor placental function and severe calorie restriction, asymmetrical FGR is common. The TCD is resistant to this influence until fetal undernutrition becomes extreme.

The INTERGROWTH-21st project has developed global gestational age norms (and errors) for the TCD.

Currently, the TraCer app can automatically identify and measure the TCD in ultrasound video loops of the fetal head in >99% of video loops. However, at present, the app is unable to automatically detect the fetal head from an ultrasound probe sweep over the maternal abdomen. This capacity is a specific aim for the TraCer app.

The TraCer initiative aim: to develop and validate automated image recognition and measurement software to capture the TCD during an ultrasound examination by minimally-trained health workers. We have (i) derived normalised values of the TCD for every day of gestational age from 16-41 weeks; (ii) developed and validated an automated image capture and measurement tool for TCD; and (iii) created a prototype TraCer mobile health (mHealth) application that can be integrated into the software package of a hand-held ultrasound machine.

We have identified the following specific hypotheses that we will test in this portion of PRECISE:

- That the TraCer tool will be acceptable to pregnant women, their families, and community decision makers.
- That the TraCer tool will be both acceptable to, and feasible for use by, health care workers and health systems' representatives.
- That the TraCer tool will be usable and informative in the hands of antenatal care providers.

We will integrate the TraCer app on a low-cost hand-held device. Currently, the TraCer system consists of a client app that automatically detects and measures the TCD ≈99% of the time and translates this into a GA. PRECISE will introduce the app onto an Android tablet with a blue-tooth-enabled basic curvilinear ultrasound probe (Konted), running the app on a low-cost tablet computer connected to a database server running a web data collection app with transmission of ultrasound data to a centralised database for quality control. The first version of the app will record video sweeps of the abdomen which will be sent to the University of Oxford for the images to be analysed and the TCDs to be measured by trained sonographers. Once the algorithm automatically calculating the TCD from the video sweeps is refined and validated by the data captured in the study, phase two of the app will be released where the TCD will be calculated automatically from the abdominal sweep. Training nurses and midwives to identify the fetal head will be carried out by a trained sonographer to ensure they locate the correct structure.

This phase will include usability- and beta-testing of the device and iterative feedback to refine the algorithm, if needed.

Qualitative data will be obtained through focus groups and in-depth interviews with health care providers and policy makers to identify current patterns of practice, barriers and facilitators for task-sharing of GA determination using the TraCer app and what level of heath care worker would be

best placed to use it. Analyses will be conducted using nVivo software in local dialect(s), prior to English translation.

See Appendix 13 for a detailed protocol, as this phase of the project is finalised, ready to proceed, and is a pre-requisite for the remainder of the initiative. Additional complementary research to validate the app may occur in Kenya as part of Dr Koech's PRECISE-embedded PhD project.

CRADLE (Lead: Shennan)

To enumerate the prevalence of pregnancy hypertension, accurate blood pressure (BP) measurement is essential. In less-developed countries, pre-eclampsia is frequently undetected as there is low attendance for antenatal care, inadequate training in BP measurement and insufficient, poorly functioning equipment. With Gates and MRC funding we have developed and clinically validated a semi-automated BP device (Microlife CRADLE Vital Signs Alert (VSA®)) specifically for use in less-developed countries.

The device comprises a micro-USB port and a sealed rechargeable Li battery pack for charging through generic mobile phone chargers. The manual inflation prolongs battery life. An integrated traffic light early-warning system alerts users to BP abnormalities. Predefined BP thresholds, used as hypertension triggers for the CLIP Trials, have been introduced as the amber and red triggers within the traffic light system. As well as being a highly accurate device suitable for LMIC settings, this device costs less than $20 USD, and was named by Innovation Countdown 2030 as one of 30 high impact innovations to save lives (another is the phone oximeter, described below).

We have identified the following specific hypotheses that we will test in this portion of PRECISE:

- That use of the CRADLE BP device will accurately identify hypertensive pregnant and non-pregnant women.
- That the identification of women with hypertension will lead to fewer adverse maternal and perinatal events.
- That, in conjunction with SpO_2 (and oximetry-estimated haemoglobin), the measurement of pulse and BP will identify women with clinically-important anaemia.

Within PRECISE, the CRADLE team will further evaluate the clinical impact of the device in identifying women with pregnancy hypertension in both urban and rural settings. The CRADLE device will be the cornerstone of our identification of hypertensive pregnancies for the epidemiological elements of PRECISE.

A user guide for the CRADLE device is appended to this protocol (Appendix 14).

Clinical phenotyping (PIERS) (Leads: Magee & von Dadelszen)

In addition to routine clinical data, we will focus on two issues, namely time-of-disease assessment and integrating translational biomarkers into risk assessment.

Time-of-disease risk assessment with miniPIERS, PIERS on the Move and phone oximetry: The LMIC-based demographics, symptom and sign-based miniPIERS (Pre-eclampsia Integrated Estimate of RiSk) prediction model provides a simple, evidence-based tool to identify pregnant women at increased risk of death or major hypertensive-related complications. The model includes: parity (nulliparous vs parous), gestational age, headache/visual disturbances, chest pain/dyspnoea, vaginal bleeding with abdominal pain, systolic BP and dipstick proteinuria (AUC ROC 0.77 [95% CI 0.74–0.80]). A predicted probability ≥25% to define a positive test classifies women with 85.5% accuracy. Thus, miniPIERS identifies women at increased risk of adverse maternal outcomes to guide $MgSO_4$ and antihypertensive therapy, or transfer to a higher level of care.

In addition, we have developed and externally validated the extended fullPIERS model that includes demographics (gestational age), symptoms (chest pain/dyspnoea), signs (SpO_2) and laboratory tests (platelet count, serum creatinine, ALT and AST) (AUC ROC 0.88 [95% CI 0·84–0·92]). External validation in the LMIC-based miniPIERS cohort confirms good fullPIERS performance in these settings.

The PIERS On the Move (POM) app integrates miniPIERS with a decision algorithm to provide mHealth support to community health workers screening women for pregnancy hypertension and initiating life-saving therapy within the community before transfer to referral centres for definitive care. Also, we have developed an integrated miniPIERS and fullPIERS app, Kenek PIERS®. SpO_2 is a significant independent predictor of risk in women with pregnancy hypertension and the addition of pulse oximetry improves the miniPIERS model. The POM app integrates phone oximetry; the Kenek Edge pulse oximeter® plugs into the audio port on a smart device to measure, record and export heart rate and SpO_2. A miniPIERS data set-based fetal time-of-disease risk model, for use ≥32+0wks includes maternal age, symptoms (0, 1 or ≥2) and dipstick proteinuria (AUC ROC 0.75 [95% CI 0.71–0.80]).

Also, we have developed the WHO Maternal Morbidity Tool, WOICE, to guide care and to track health systems (see Epidemiology and co-exposures, above).

We have identified the following specific hypotheses that we will test in this portion of PRECISE:

- That the miniPIERS model will be externally-validated in three new geographies.
- That a fetal outcome prediction model can be developed and validated in this project.
- That a LMIC prediction of placental disease model can be developed and validated in this project.

PRECISE provides the opportunity to externally validate the miniPIERS and fullPIERS models in new LMIC settings, using standard epidemiological approaches with which we have experience; standard measures of diagnostic accuracy include stratification capacity, calibration ability and classification.

We will develop and validate a fetal outcome prediction model for women with complex pregnancies building on previous miniPIERS experience.

By modifying the approach taken by the Fetal Medicine Foundation, we will develop and validate a less-developed country-relevant outcome prediction model that identifies women at increased risk of developing one or more placental disease(s).

A summary of the approach to be used in the portion of the project is in development and will be added in due course.

Q. *Advocacy (Working Group: Temmerman, , von Dadelszen)*

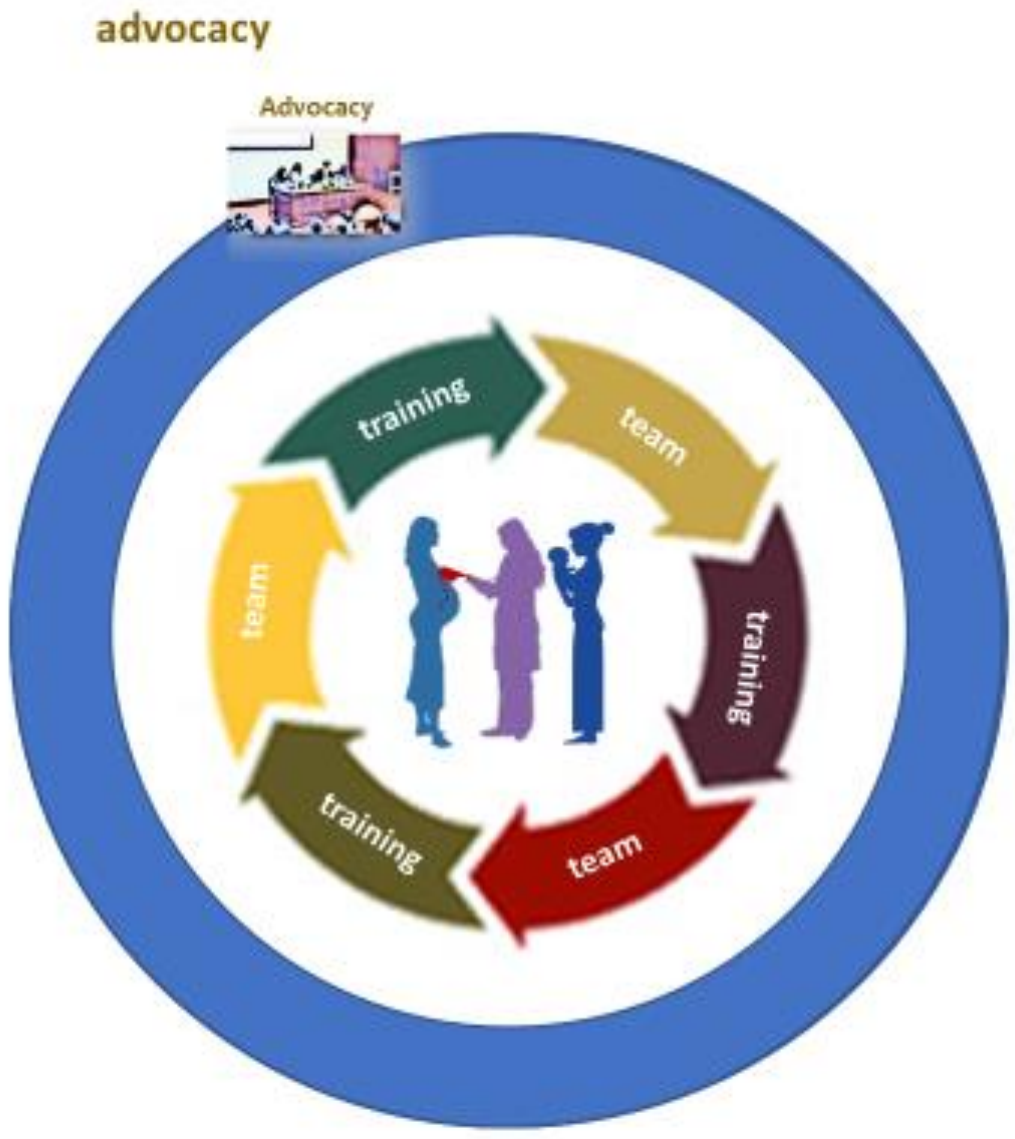

Our team has experience working in the WHO, leading the FIGO Safe Motherhood and Newborn Health Committee and the relevant maternal, fetal, newborn and under-5 mortality and health *Lancet* series.

We will embed advocacy and engagement with governments, multilaterals and NGOs. Critical partners will be those that ultimately fund the implementation of the PRECISE evidence-based interventions. They will be encouraged to share project 'ownership' from the design phase onwards.

This thematic working group will include two stakeholders from each country, one embedded at the interface between maternity care and ministry of health and the other from either the Ministry of Higher Education, Ministry of Science or the national research funding body.

More detail on the advocacy approach and framework can be seen in Appendix 3.

References

1. von Dadelszen P, Magee LA. Preventing deaths due to the hypertensive disorders of pregnancy. *Best Pract Res Clin Obstet Gynaecol* 2016; **36**: 83-102.
2. von Dadelszen P, Magee LA, Payne BA, et al. Moving beyond silos: How do we provide distributed personalized medicine to pregnant women everywhere at scale? Insights from PRE-EMPT. *Int J Gynaecol Obstet* 2015; **131 Suppl 1**: S10-5.
3. Munguambe K, Boene H, Vidler M, et al. Barriers and facilitators to health care seeking behaviours in pregnancy in rural communities of southern Mozambique. *Reprod Health* 2016; **13 Suppl 1**: 31.
4. Boene H, Vidler M, Sacoor C, et al. Community perceptions of pre-eclampsia and eclampsia in southern Mozambique. *Reprod Health* 2016; **13 Suppl 1**: 33.
5. Boene H, Vidler M, Augusto O, et al. Community health worker knowledge and management of pre-eclampsia in southern Mozambique. *Reprod Health* 2016; **13**(Suppl 2): 105.
6. Khowaja AR, Qureshi RN, Sawchuck D, et al. The feasibility of community level interventions for pre-eclampsia in South Asia and Sub-Saharan Africa: a mixed-methods design. *Reprod Health* 2016; **13 Suppl 1**: 56.
7. de Greeff A, Shennan AH. Clinical accuracy of a low cost portable blood pressure device in pregnancy and pre-eclampsia: the Nissei DS-400. *Trop Doct* 2015; **45**(3): 168-73.
8. El Ayadi AM, Nathan HL, Seed PT, et al. Vital sign prediction of adverse maternal outcomes in women with hypovolemic shock: the role of shock index. *PLoS One* 2016; **11**(2): e0148729.
9. Nathan HL, Boene H, Munguambe K, et al. The CRADLE vital signs alert: qualitative evaluation of a novel device designed for use in pregnancy by healthcare workers in low-resource settings. *Reprod Health* 2018; **15**(1): 5.
10. Nathan HL, de Greeff A, Hezelgrave NL, Chappell LC, Shennan AH. Accuracy validation of the Microlife 3AS1-2 blood pressure device in a pregnant population with low blood pressure. *Blood Press Monit* 2015; **20**(5): 299-302.
11. Nathan HL, de Greeff A, Hezelgrave NL, Chappell LC, Shennan AH. An accurate semiautomated oscillometric blood pressure device for use in pregnancy (including pre-eclampsia) in a low-income and middle-income country population: the Microlife 3AS1-2. *Blood Press Monit* 2015; **20**(1): 52-5.
12. Nathan HL, El Ayadi A, Hezelgrave NL, et al. Shock index: an effective predictor of outcome in postpartum haemorrhage? *BJOG* 2015; **122**(2): 268-75.
13. Nathan HL, Seed PT, Hezelgrave NL, et al. Early warning system hypertension thresholds to predict adverse outcomes in pre-eclampsia: A prospective cohort study. *Pregnancy Hypertens* 2017.
14. Payne BA, Hutcheon JA, Dunsmuir D, et al. Assessing the incremental value of blood oxygen saturation (SpO(2)) in the miniPIERS (Pre-eclampsia Integrated Estimate of RiSk) Risk Prediction Model. *J Obstet Gynaecol Can* 2015; **37**(1): 16-24.
15. Garde A, Karlen W, Dehkordi P, Ansermino JM, Dumont GA. Oxygen saturation resolution influences regularity measurements. *Conf Proc IEEE Eng Med Biol Soc* 2014; **2014**: 2257-60.
16. Garde A, Zhou G, Raihana S, et al. Respiratory rate and pulse oximetry derived information as predictors of hospital admission in young children in Bangladesh: a prospective observational study. *BMJ Open* 2016; **6**(8): e011094.
17. Ginsburg AS, Lenahan JL, Izadnegahdar R, Ansermino JM. A systematic review of tools to measure respiratory rate in order to identify childhood pneumonia. *Am J Respir Crit Care Med* 2018; **197**(9): 1116-27.
18. Gorges M, West NC, Karlsdottir E, Ansermino JM, Cassidy M, Lauder GR. Developing an objective method for analyzing vital signs changes in neonates during general anesthesia. *Paediatr Anaesth* 2016; **26**(11): 1071-81.
19. Wiens MO, Kissoon N, Kumbakumba E, et al. Selecting candidate predictor variables for the modelling of post-discharge mortality from sepsis: a protocol development project. *Afr Health Sci* 2016; **16**(1): 162-9.

20. Wiens MO, Kumbakumba E, Larson CP, et al. Postdischarge mortality in children with acute infectious diseases: derivation of postdischarge mortality prediction models. *BMJ Open* 2015; **5**(11): e009449.
21. Akkermans J, Payne B, von Dadelszen P, et al. Predicting complications in pre-eclampsia: external validation of the fullPIERS model using the PETRA trial dataset. *Eur J Obstet Gynecol Reprod Biol* 2014; **179**: 58-62.
22. von Dadelszen P, Payne B, Li J, et al. Prediction of adverse maternal outcomes in pre-eclampsia: development and validation of the fullPIERS model. *Lancet* 2011; **377**(9761): 219-27.
23. Yen TW, Payne B, Qu Z, et al. Using clinical symptoms to predict adverse maternal and perinatal outcomes in women with preeclampsia: data from the PIERS (Pre-eclampsia Integrated Estimate of RiSk) study. *J Obstet Gynaecol Can* 2011; **33**(8): 803-9.
24. Millman AL, Payne B, Qu Z, et al. Oxygen saturation as a predictor of adverse maternal outcomes in women with preeclampsia. *J Obstet Gynaecol Can* 2011; **33**(7): 705-14.
25. Kozic JR, Benton SJ, Hutcheon JA, et al. Abnormal liver function tests as predictors of adverse maternal outcomes in women with preeclampsia. *J Obstet Gynaecol Can* 2011; **33**(10): 995-1004.
26. Laskin S, Payne B, Hutcheon JA, et al. The role of platelet counts in the assessment of inpatient women with preeclampsia. *J Obstet Gynaecol Can* 2011; **33**(9): 900-8.
27. Payne B, Magee LA, Cote AM, et al. PIERS proteinuria: relationship with adverse maternal and perinatal outcome. *J Obstet Gynaecol Can* 2011; **33**(6): 588-97.
28. Payne BA, Groen H, Ukah UV, et al. Development and internal validation of a multivariable model to predict perinatal death in pregnancy hypertension. *Pregnancy Hypertens* 2015; **5**(4): 315-21.
29. Payne B, Hodgson S, Hutcheon JA, et al. Performance of the fullPIERS model in predicting adverse maternal outcomes in pre-eclampsia using patient data from the PIERS (Pre-eclampsia Integrated Estimate of RiSk) cohort, collected on admission. *BJOG* 2013; **120**(1): 113-8.
30. Ukah UV, Payne B, Hutcheon JA, et al. Assessment of the fullPIERS risk prediction model in women with early-onset preeclampsia. *Hypertension* 2018; **71**(4): 659-65.
31. Ukah UV, Payne B, Lee T, et al. External validation of the fullPIERS model for predicting adverse maternal outcomes in pregnancy hypertension in low- and middle-income countries. *Hypertension* 2017; **69**(4): 705-11.
32. Dunsmuir DT, Payne BA, Cloete G, et al. Development of mHealth applications for pre-eclampsia triage. *IEEE J Biomed Health Inform* 2014; **18**(6): 1857-64.
33. Lim J, Cloete G, Dunsmuir DT, et al. Usability and feasibility of PIERS on the Move: an mHealth app for pre-eclampsia triage. *JMIR Mhealth Uhealth* 2015; **3**(2): e37.
34. Livingston JR, Payne B, Brown M, et al. Uric acid as a predictor of adverse maternal and perinatal outcomes in women hospitalized with preeclampsia. *J Obstet Gynaecol Can* 2014; **36**(10): 870-7.
35. Filippi V, Chou D, Ronsmans C, Graham W, Say L. Levels and causes of maternal mortality and morbidity. In: Black RE, Laxminarayan R, Temmerman M, Walker N, eds. Reproductive, Maternal, Newborn, and Child Health: Disease Control Priorities, Third Edition (Volume 2). Washington (DC); 2016.
36. Chou D, Tuncalp O, Firoz T, et al. Constructing maternal morbidity - towards a standard tool to measure and monitor maternal health beyond mortality. *BMC Pregnancy Childbirth* 2016; **16**: 45.
37. Say L, Barreix M, Chou D, et al. Maternal morbidity measurement tool pilot: study protocol. *Reprod Health* 2016; **13**(1): 69.
38. Firoz T, Chou D, von Dadelszen P, et al. Measuring maternal health: focus on maternal morbidity. *Bull World Health Organ* 2013; **91**(10): 794-6.
39. Leftwich HK, Alves MV. Adolescent pregnancy. *Pediatr Clin North Am* 2017; **64**(2): 381-8.
40. Makanga PT, Schuurman N, Sacoor C, et al. Seasonal variation in geographical access to maternal health services in regions of southern Mozambique. *Int J Health Geogr* 2017; **16**(1): 1.

41. Krauskopf J, Caiment F, van Veldhoven K, et al. The human circulating miRNome reflects multiple organ disease risks in association with short-term exposure to traffic-related air pollution. *Environ Int* 2018; **113**: 26-34.
42. Moore E, Chatzidiakou L, Jones RL, et al. Linking e-health records, patient-reported symptoms and environmental exposure data to characterise and model COPD exacerbations: protocol for the COPE study. *BMJ Open* 2016; **6**(7): e011330.
43. Walton RT, Mudway IS, Dundas I, et al. Air pollution, ethnicity and telomere length in east London schoolchildren: An observational study. *Environ Int* 2016; **96**: 41-7.
44. Gon G, Restrepo-Mendez MC, Campbell OM, et al. Who delivers without water? A multi Country analysis of water and sanitation in the childbirth environment. *PLoS One* 2016; **11**(8): e0160572.
45. Akeju DO, Oladapo OT, Vidler M, et al. Determinants of health care seeking behaviour during pregnancy in Ogun State, Nigeria. *Reprod Health* 2016; **13 Suppl 1**: 32.
46. Akeju DO, Vidler M, Oladapo OT, et al. Community perceptions of pre-eclampsia and eclampsia in Ogun State, Nigeria: a qualitative study. *Reprod Health* 2016; **13 Suppl 1**: 57.
47. Akeju DO, Vidler M, Sotunsa JO, et al. Human resource constraints and the prospect of task-sharing among community health workers for the detection of early signs of pre-eclampsia in Ogun State, Nigeria. *Reprod Health* 2016; **13**(Suppl 2): 111.
48. Bellad MB, Vidler M, Honnungar NV, et al. Maternal and newborn health in Karnataka State, India: the Community Level Interventions for Pre-Eclampsia (CLIP) trial's baseline study results. *PLoS One* 2017; **12**(1): e0166623.
49. Firoz T, Vidler M, Makanga PT, et al. Community perspectives on the determinants of maternal health in rural southern Mozambique: a qualitative study. *Reprod Health* 2016; **13**(Suppl 2): 112.
50. Khowaja AR, Mitton C, Bryan S, Magee LA, Bhutta ZA, von Dadelszen P. Economic evaluation of Community Level Interventions for Pre-eclampsia (CLIP) in South Asian and African countries: a study protocol. *Implement Sci* 2015; **10**: 76.
51. Khowaja AR, Mitton C, Qureshi R, et al. Societal perspective on cost drivers for health technology assessment in Sindh, Pakistan. *Int J Technol Assess Health Care* 2017; **33**(2): 192-8.
52. Khowaja AR, Qureshi RN, Sheikh S, et al. Community's perceptions of pre-eclampsia and eclampsia in Sindh Pakistan: a qualitative study. *Reprod Health* 2016; **13 Suppl 1**: 36.
53. Ramadurg U, Vidler M, Charanthimath U, et al. Community health worker knowledge and management of pre-eclampsia in rural Karnataka State, India. *Reprod Health* 2016; **13**(Suppl 2): 113.
54. Sacoor C, Payne B, Augusto O, et al. Health and socio-demographic profile of women of reproductive age in rural communities of southern Mozambique. *PLoS One* 2018; **13**(2): e0184249.
55. Salam RA, Qureshi RN, Sheikh S, et al. Potential for task-sharing to Lady Health Workers for identification and emergency management of pre-eclampsia at community level in Pakistan. *Reprod Health* 2016; **13**(Suppl 2): 107.
56. Sharma S, Adetoro OO, Vidler M, et al. A process evaluation plan for assessing a complex community-based maternal health intervention in Ogun State, Nigeria. *BMC Health Serv Res* 2017; **17**(1): 238.
57. Sheikh S, Qureshi RN, Khowaja AR, et al. Health care provider knowledge and routine management of pre-eclampsia in Pakistan. *Reprod Health* 2016; **13**(Suppl 2): 104.
58. Sotunsa JO, Vidler M, Akeju DO, et al. Community health workers' knowledge and practice in relation to pre-eclampsia in Ogun State, Nigeria: an essential bridge to maternal survival. *Reprod Health* 2016; **13**(Suppl 2): 108.
59. Vidler M, Charantimath U, Katageri G, et al. Community perceptions of pre-eclampsia in rural Karnataka State, India: a qualitative study. *Reprod Health* 2016; **13 Suppl 1**: 35.
60. Vidler M, Magee LA, von Dadelszen P, et al. Women's views and postpartum follow-up in the CHIPS Trial (Control of Hypertension in Pregnancy Study). *Eur J Obstet Gynecol Reprod Biol* 2016; **206**: 105-13.

61. Vidler M, Ramadurg U, Charantimath U, et al. Utilization of maternal health care services and their determinants in Karnataka State, India. *Reprod Health* 2016; **13 Suppl 1**: 37.
62. Campbell OM, Calvert C, Testa A, et al. The scale, scope, coverage, and capability of childbirth care. *Lancet* 2016; **388**(10056): 2193-208.
63. de Savigny DT, A. Systems thinking for health systems strengthening. Geneva, 2009.
64. Lohmann J, Souares A, Tiendrebeogo J, et al. Measuring health workers' motivation composition: validation of a scale based on Self-Determination Theory in Burkina Faso. *Hum Resour Health* 2017; **15**(1): 33.
65. Benton SJ, Hu Y, Xie F, et al. Angiogenic factors as diagnostic tests for preeclampsia: a performance comparison between two commercial immunoassays. *Am J Obstet Gynecol* 2011; **205**(5): 469 e1-8.
66. Benton SJ, Hu Y, Xie F, et al. Can placental growth factor in maternal circulation identify fetuses with placental intrauterine growth restriction? *Am J Obstet Gynecol* 2012; **206**(2): 163 e1-7.
67. Benton SJ, McCowan LM, Heazell AE, et al. Placental growth factor as a marker of fetal growth restriction caused by placental dysfunction. *Placenta* 2016; **42**: 1-8.
68. Manriquez Rocha B, Mbofana F, Loquiha O, et al. Early diagnosis of preeclampsia using placental growth factor: An operational pilot study in Maputo, Mozambique. *Pregnancy Hypertens* 2018; **11**: 26-31.
69. Ukah UV, Hutcheon JA, Payne B, et al. Placental growth factor as a prognostic tool in women with hypertensive disorders of pregnancy: a systematic review. *Hypertension* 2017; **70**(6): 1228-37.
70. Ukah UV, Mbofana F, Rocha BM, et al. Diagnostic performance of placental growth factor in women with suspected preeclampsia attending antenatal facilities in Maputo, Mozambique. *Hypertension* 2017; **69**(3): 469-74.
