# Supplementary material for: Evaluating the association between household water, sanitation and hygiene (WASH) and selected placenta-related complications in The Gambia, Kenya and Mozambique
Source: PLOS Glob Public Health. 2026 Oct 15;6(10):e0005004. doi: 10.1371/journal.pgph.0005004 (PMC13671507; doi:10.1371/journal.pgph.0005004)
Supplement: Supplementary file 2 [file pgph.0005004.s001.docx]

**S1 Table. Baseline characteristics of participants with and without size‑for‑gestational‑age data in the PRECISE cohort in The Gambia, Kenya and Mozambique.**

| **Characteristic** | **SGA** | |
| --- | --- | --- |
|  | **Not missing,**  **N = 4,957 (86.3%)** | **Missing,**  **N = 788 (13.7%)** |
| **Age at enrolment, median (IQR)** | 25 (21, 30) | 26 (22, 31) |
| Unknown | 1 | 5 |
| **Age at enrolment (years)** |  |  |
| 15-19 | 747 (15.1%) | 70 (8.9%) |
| 20-24 | 1,456 (29.4%) | 227 (29.0%) |
| 25-29 | 1,313 (26.5%) | 227 (29.0%) |
| 30-34 | 868 (17.5%) | 150 (19.2%) |
| 35+ | 572 (11.5%) | 109 (13.9%) |
| Unknown | 1 | 5 |
| **Parity** |  |  |
| 0 | 1,510 (30.5%) | 185 (23.5%) |
| 1 | 1,196 (24.1%) | 174 (22.1%) |
| 2-4 | 1,817 (36.7%) | 312 (39.6%) |
| 5+ | 434 (8.8%) | 117 (14.8%) |
| **Maternal BMI, median (IQR)** | 23.9 (21.4, 27.2) | 23.3 (20.7, 26.6) |
| Unknown | 22 | 4 |
| **Maternal BMI categorised** |  |  |
| <18.5 - Underweight | 239 (4.8%) | 54 (6.9%) |
| 18.5-24.9 - Normal | 2,726 (55.2%) | 463 (59.1%) |
| 25-29.9 - Overweight | 1,345 (27.3%) | 172 (21.9%) |
| +30 - Obese | 625 (12.7%) | 95 (12.1%) |
| Unknown | 22 | 4 |
| **Average MUAC, median (IQR)** | 26.8 (24.6, 29.5) | 27.5 (24.3, 29.4) |
| Unknown | 17 | 5 |
| **Average MUAC categorised (cm)** |  |  |
| <23 – Underweight | 469 (9.6%) | 80 (10.4%) |
| 23-26.4 – Normal | 1,733 (35.6%) | 291 (37.8%) |
| 26.5-29.9 - Overweight | 1,538 (31.6%) | 235 (30.5%) |
| +30 – Obese | 1,132 (23.2%) | 164 (21.3%) |
| Unknown | 85 | 18 |
| **Household location** |  |  |
| Rural | 1,447 (29.9%) | 237 (31.5%) |
| Peri-urban | 729 (15.1%) | 150 (19.9%) |
| Urban | 2,665 (55.1%) | 365 (48.5%) |
| Unknown | 116 | 36 |
| **Highest education level** |  |  |
| None | 857 (17.3%) | 227 (29.0%) |
| Primary | 1,920 (38.7%) | 334 (42.7%) |
| Secondary | 1,841 (37.2%) | 171 (21.8%) |
| Higher | 337 (6.8%) | 51 (6.5%) |
| Unknown | 2 | 5 |
| **% likelihood below the poverty line, median (IQR)** | 21.4 (6.1, 37.4) | 27.9 (6.1, 50.8) |
| Unknown | 2 | 6 |
| **Pre-eclampsia** | 410 (8.3%) | 44 (5.6%) |
| Unknown | 3 | 4 |
| **Stillbirth** | 112 (2.3%) | 67 (8.5%) |

Abbreviations: BMI, body mass index; IQR, interquartile range; MUAC, mid-upper arm circumference; SGA, small-for-gestational-age
