# Supplementary material for: Evaluating the association between household water, sanitation and hygiene (WASH) and selected placenta-related complications in The Gambia, Kenya and Mozambique
Source: PLOS Glob Public Health. 2026 Oct 15;6(10):e0005004. doi: 10.1371/journal.pgph.0005004 (PMC13671507; doi:10.1371/journal.pgph.0005004)
Supplement: Supplementary file 3 [file pgph.0005004.s002.docx]

**S2 Table. Characteristics and prevalence of selected placenta-related complications in the PRECISE cohort in The Gambia, Kenya and Mozambique.**

| **Characteristic** | **The Gambia, N = 1,178** | **Kenya, N = 2,706** | **Mozambique, N = 1,861** | **PRECISE, N = 5,745** |
| --- | --- | --- | --- | --- |
| **Age at enrolment, median (IQR)** | 26.0 (22.0, 31.0) | 26.0 (23.0, 31.0) | 23.0 (19.0, 29.0) | 26.0 (21.0, 31.0) |
| Unknown | 4 | 2 | 0 | 6 |
| **Age at enrolment (years)** |  |  |  |  |
| 15-19 | 145 (12.4%) | 183 (6.8%) | 489 (26.3%) | 817 (14.2%) |
| 20-24 | 311 (26.5%) | 823 (30.4%) | 549 (29.5%) | 1,683 (29.3%) |
| 25-29 | 343 (29.2%) | 796 (29.4%) | 401 (21.5%) | 1,540 (26.8%) |
| 30-34 | 213 (18.1%) | 564 (20.9%) | 241 (13.0%) | 1,018 (17.7%) |
| 35-49 | 162 (13.8%) | 338 (12.5%) | 181 (9.7%) | 681 (11.9%) |
| Unknown | 4 | 2 | 0 | 6 |
| **Gestational age at enrolment, median (IQR), weeks** | 21.4 (15.4, 27.1) | 21.7 (16.7, 26.4) | 19.6 (15.6, 23.4) | 20.9 (16.1, 25.4) |
| Unknown | 45 | 59 | 7 | 111 |
| **Duration of follow-up from enrolment to delivery, median (IQR), weeks** | 16.9 (11.3, 22.7) | 17.1 (12.3, 22.0) | 19.4 (15.3, 23.9) | 18.0 (13.0, 22.9) |
| Unknown | 22 | 21 | 0 | 43 |
| **Parity** |  |  |  |  |
| 0 | 217 (18.4%) | 756 (27.9%) | 722 (38.8%) | 1,695 (29.5%) |
| 1 | 195 (16.6%) | 726 (26.8%) | 449 (24.1%) | 1,370 (23.8%) |
| 2-4 | 480 (40.7%) | 1,015 (37.5%) | 634 (34.1%) | 2,129 (37.1%) |
| 5+ | 286 (24.3%) | 209 (7.7%) | 56 (3.0%) | 551 (9.6%) |
| **Maternal BMI, median (IQR)** | 22.1 (19.8, 24.8) | 24.2 (21.6, 27.9) | 24.3 (22.1, 27.1) | 23.8 (21.3, 27.1) |
| Unknown | 1 | 25 | 0 | 26 |
| **Maternal BMI categorised** |  |  |  |  |
| <18.5 - Underweight | 153 (13.0%) | 104 (3.9%) | 36 (1.9%) | 293 (5.1%) |
| 18.5-24.9 - Normal | 750 (63.7%) | 1,423 (53.1%) | 1,016 (54.6%) | 3,189 (55.8%) |
| 25-29.9 - Overweight | 200 (17.0%) | 710 (26.5%) | 607 (32.6%) | 1,517 (26.5%) |
| +30 - Obese | 74 (6.3%) | 444 (16.6%) | 202 (10.9%) | 720 (12.6%) |
| Unknown | 1 | 25 | 0 | 26 |
| **MUAC, median (IQR)** | 26.3 (24.2, 28.8) | 26.5 (24.0, 29.5) | 27.5 (25.6, 30.0) | 26.8 (24.5, 29.5) |
| Unknown | 2 | 17 | 3 | 22 |
| **Average MUAC categorised (cm)** |  |  |  |  |
| <23 – Underweight | 123 (10.7%) | 374 (14.2%) | 52 (2.8%) | 549 (9.7%) |
| 23-26.4 – Normal | 470 (40.8%) | 951 (36.0%) | 603 (32.6%) | 2,024 (35.9%) |
| 26.5-29.9 - Overweight | 343 (29.7%) | 719 (27.2%) | 711 (38.5%) | 1,773 (31.4%) |
| +30 – Obese | 217 (18.8%) | 596 (22.6%) | 483 (26.1%) | 1,296 (23.0%) |
| Unknown | 25 | 66 | 12 | 103 |
| **Household location** |  |  |  |  |
| Rural | 713 (61.8%) | 198 (7.7%) | 773 (41.5%) | 1,684 (30.1%) |
| Peri-urban | 10 (0.9%) | 824 (32.0%) | 45 (2.4%) | 879 (15.7%) |
| Urban | 431 (37.3%) | 1,556 (60.4%) | 1,043 (56.0%) | 3,030 (54.2%) |
| Unknown | 24 | 128 | 0 | 152 |
| **Highest education level** |  |  |  |  |
| None | 751 (63.8%) | 229 (8.5%) | 104 (5.6%) | 1,084 (18.9%) |
| Primary | 187 (15.9%) | 1,446 (53.6%) | 621 (33.4%) | 2,254 (39.3%) |
| Secondary | 190 (16.1%) | 706 (26.2%) | 1,116 (60.0%) | 2,012 (35.1%) |
| Higher | 50 (4.2%) | 318 (11.8%) | 20 (1.1%) | 388 (6.8%) |
| Unknown | 0 | 7 | 0 | 7 |
| **% likelihood below the poverty line, median (IQR)** | 46.9 (27.9, 58.6) | 13.9 (4.6, 30.0) | 21.4 (7.2, 31.7) | 21.4 (6.1, 42.3) |
| Unknown | 0 | 8 | 0 | 8 |
| **Pre-eclampsia** | 172 (14.6%) | 228 (8.4%) | 54 (2.9%) | 454 (7.9%) |
| Unknown | 1 | 6 | 0 | 7 |
| **Size for gestational age** |  |  |  |  |
| Very Small for Gestational Age (<3rd centile) | 72 (8.0%) | 168 (7.4%) | 141 (7.9%) | 381 (7.7%) |
| Small for Gestational Age (<10th centile) | 110 (12.2%) | 262 (11.6%) | 202 (11.3%) | 574 (11.6%) |
| Appropriate for Gestational Age (10th to 90th centile) | 609 (67.8%) | 1,582 (69.8%) | 1,297 (72.4%) | 3,488 (70.4%) |
| Large for Gestational Age (>90th centile) | 69 (7.7%) | 176 (7.8%) | 96 (5.4%) | 341 (6.9%) |
| Very Large for Gestational Age (>97th centile) | 38 (4.2%) | 80 (3.5%) | 55 (3.1%) | 173 (3.5%) |
| Unknown | 280 | 438 | 70 | 788 |
| **Stillbirth** | 58 (4.9%) | 60 (2.2%) | 61 (3.3%) | 179 (3.1%) |
| **Selected placenta-related complications: Pre-eclampsia, small-for-gestational-age (SGA < 10th percentile) or stillbirth** | 305 (34.0%) | 621 (27.4%) | 409 (22.8%) | 1,335 (26.9%) |
| Unknown | 280 | 438 | 70 | 788 |
