# Supplementary material for: Evaluating the association between household water, sanitation and hygiene (WASH) and selected placenta-related complications in The Gambia, Kenya and Mozambique
Source: PLOS Glob Public Health. 2026 Oct 15;6(10):e0005004. doi: 10.1371/journal.pgph.0005004 (PMC13671507; doi:10.1371/journal.pgph.0005004)
Supplement: Supplementary file 4 [file pgph.0005004.s003.docx]

**S3 Table. Water, sanitation and hygiene facilities and practices in the households of pregnant women in the PRECISE cohort in The Gambia, Kenya and Mozambique.**

| **Variable** | **The Gambia,**  **N = 1,178** | **Kenya,**  **N = 2,706** | **Mozambique,**  **N = 1,861** | **PRECISE,**  **N = 5,745** |
| --- | --- | --- | --- | --- |
| **Source of drinking water** |  |  |  |  |
| Piped water into dwelling/plot | 341 (29.1%) | 1,204 (44.7%) | 1,320 (71.0%) | 2,865 (50.1%) |
| Other improved water source (piped water into neighbour’s dwelling or public tap, boreholes or tubewells, protected dug wells, protected springs, rainwater, and packaged or delivered water) | 792 (67.6%) | 1,396 (51.9%) | 347 (18.7%) | 2,535 (44.3%) |
| Unimproved water source (surface water, unprotected well or spring) | 39 (3.3%) | 91 (3.4%) | 191 (10.3%) | 321 (5.6%) |
| Unknown | 6 | 15 | 3 | 24 |
| **Source of non-drinking water (used for cooking/washing/personal hygiene)** |  |  |  |  |
| Piped water into dwelling/plot | 349 (29.7%) | 1,228 (45.6%) | 1,309 (70.5%) | 2,886 (50.4%) |
| Other improved water source (piped water into neighbour’s dwelling or public tap, boreholes or tubewells, protected dug wells, protected springs, rainwater, and packaged or delivered water) | 767 (65.3%) | 1,358 (50.4%) | 354 (19.1%) | 2,479 (43.3%) |
| Unimproved water source (surface water, unprotected well or spring) | 58 (4.9%) | 108 (4.0%) | 195 (10.5%) | 361 (6.3%) |
| Unknown | 4 | 12 | 3 | 19 |
| **Alternative drinking and non-drinking water source** | 37 (3.2%) | 70 (2.6%) | 88 (4.7%) | 195 (3.4%) |
| Unknown | 6 | 15 | 4 | 25 |
| **Location of water source** |  |  |  |  |
| In own dwelling | 71 (6.0%) | 474 (17.6%) | 215 (11.6%) | 760 (13.3%) |
| In own yard or plot | 326 (27.7%) | 837 (31.0%) | 1,301 (69.9%) | 2,464 (43.0%) |
| Elsewhere | 781 (66.3%) | 1,386 (51.4%) | 344 (18.5%) | 2,511 (43.8%) |
| Unknown | 0 | 9 | 1 | 10 |
| **Time to collect water if collected outside the household, median (IQR)** | 5.0 (3.0, 10.0) | 5.0 (2.0, 10.0) | 3.0 (2.0, 5.0) | 5.0 (2.0, 10.0) |
| **Sanitation facility** |  |  |  |  |
| Improved (flush toilets connected to piped sewer systems, septic tanks or pit latrines; pit latrines with slabs, composting toilets) | 727 (61.9%) | 2,351 (87.3%) | 1,490 (80.5%) | 4,568 (79.9%) |
| Unimproved (pit latrines without a slab or platform, hanging latrines, bucket latrines or open defecation) | 448 (38.1%) | 342 (12.7%) | 360 (19.5%) | 1,150 (20.1%) |
| Unknown | 3 | 13 | 11 | 27 |
| **Sanitation facility location** |  |  |  |  |
| In own dwelling | 268 (22.8%) | 724 (26.8%) | 194 (10.4%) | 1,186 (20.7%) |
| In own yard or plot | 849 (72.1%) | 1,867 (69.2%) | 1,608 (86.5%) | 4,324 (75.4%) |
| Elsewhere | 61 (5.2%) | 106 (3.9%) | 57 (3.1%) | 224 (3.9%) |
| Unknown | 0 | 9 | 2 | 11 |
| **Share sanitation facility with non-household members** | 421 (35.7%) | 1,484 (55.0%) | 166 (8.9%) | 2,071 (36.1%) |
| Unknown | 0 | 9 | 6 | 15 |
| **Handwashing facility** |  |  |  |  |
| Fixed facility (sink/tap) in dwelling | 20 (1.7%) | 352 (13.2%) | 90 (4.8%) | 462 (8.1%) |
| Fixed facility (sink/tap) in yard/plot | 20 (1.7%) | 187 (7.0%) | 110 (5.9%) | 317 (5.6%) |
| Mobile object (bucket/jug/kettle) | 1,065 (91.0%) | 1,731 (65.0%) | 1,653 (89.0%) | 4,449 (78.2%) |
| No handwashing place in dwelling or yard/plot | 65 (5.6%) | 392 (14.7%) | 4 (0.2%) | 461 (8.1%) |
| Unknown | 8 | 44 | 4 | 56 |

IQR, interquartile range; PRECISE, PREgnancy Care Integrating translational Science, Everywhere
