# Supplementary material for: Evaluating the association between household water, sanitation and hygiene (WASH) and selected placenta-related complications in The Gambia, Kenya and Mozambique
Source: PLOS Glob Public Health. 2026 Oct 15;6(10):e0005004. doi: 10.1371/journal.pgph.0005004 (PMC13671507; doi:10.1371/journal.pgph.0005004)
Supplement: Supplementary file 5 [file pgph.0005004.s004.docx]

**S4 Table. Comparison of Joint Monitoring Programme 2022 national WASH data and the PRECISE cohort in The Gambia, Kenya and Mozambique.**

| **JMP Ladders** | **The Gambia** | | | | **Kenya** | | | | **Mozambique** | | | |
| --- | --- | --- | --- | --- | --- | --- | --- | --- | --- | --- | --- | --- |
|  | **Rural** | | **Urban** | | **Rural** | | **Urban** | | **Rural** | | **Urban** | |
|  | **JMP data, %** | **PRECISE* (N=713)** | **JMP data, %** | **PRECISE (N=431)** | **JMP data, %** | **PRECISE (N = 198)** | **JMP data, %** | **PRECISE (N = 1,556)** | **JMP data,**  **%** | **PRECISE (N = 773)** | **JMP data, %** | **PRECISE**  **(N = 1,043)** |
| **Water** |  |  |  |  |  |  |  |  |  |  |  |  |
| At least basic | 76.4 | 611 (95.0%) | 90.9 | 401 (96.6%) | 53.3 | 186 (94.9%) | 86.4 | 1,448 (94.5%) | 48.3 | 642 (83.4%) | 87.3 | 963 (93.3%) |
| Limited | 12.5 | 2 (0.3%) | 2.2 | 5 (1.2%) | 10.7 | 7 (3.6%) | 3.6 | 61 (4.0%) | 12.5 | 5 (0.6%) | 5.4 | 5 (0.5%) |
| Unimproved | 11.0 | 30 (4.7%) | 6.9 | 9 (2.2%) | 11.5 | 2 (1.0%) | 4.0 | 10 (0.7%) | 23.7 | 122 (15.8%) | 5.3 | 62 (6.0%) |
| Surface water | 0 | 0 (0.0%) | 0.1 | 0 (0.0%) | 24.5 | 1 (0.5%) | 6.0 | 13 (0.8%) | 15.4 | 1 (0.1%) | 2.1 | 2 (0.2%) |
| Unknown |  | 70 |  | 16 |  | 2 |  | 24 |  | 3 |  | 11 |
| **Sanitation** |  |  |  |  |  |  |  |  |  |  |  |  |
| At least basic | 24.0 | 313 (44.0%) | 61.1 | 190 (44.1%) | 35.2 | 70 (35.4%) | 39.8 | 621 (39.9%) | 22.6 | 567 (73.5%) | 61.3 | 753 (73.0%) |
| Limited | 7.9 | 78 (11.0%) | 13.8 | 124 (28.8%) | 15.9 | 109 (55.1%) | 44.9 | 772 (49.6%) | 1.7 | 35 (4.5%) | 10.5 | 97 (9.4%) |
| Unimproved | 68 | 314 (44.1%) | 25 | 115 (26.7%) | 40.2 | 16 (8.1%) | 14.4 | 151 (9.7%) | 47.3 | 168 (21.8%) | 22.9 | 178 (17.3%) |
| OD | 0.0 | 7 (1.0%) | 0.1 | 2 (0.5%) | 8.7 | 3 (1.5%) | 0.9 | 12 (0.8%) | 28.5 | 1 (0.1%) | 5.2 | 3 (0.3%) |
| Unknown |  | 1 |  | 0 |  | 0 |  | 0 |  | 2 |  | 12 |
| **Hygiene** |  |  |  |  |  |  |  |  |  |  |  |  |
| Basic | 11.9 | NA | 13.5 | NA | 34.6 | NA | 44.8 | NA | NA | NA | NA | NA |
| Limited^1^ | 80.2 | 660 (93.4%) | 77.4 | 413 (96.3%) | 27.4 | 158 (80.6%) | 31.3 | 1,329 (86.6%) | NA | 770 (99.9%) | NA | 1,038 (99.7%) |
| No facility | 7.9 | 47 (6.6%) | 9.1 | 16 (3.7%) | 38 | 38 (19.4%) | 23.9 | 206 (13.4%) | NA | 1 (0.1%) | NA | 3 (0.3%) |
| Unknown |  | 6 |  | 2 |  | 2 |  | 21 |  | 2 |  | 2 |

Note: this does not include peri-urban data. All national WASH data sourced from the Joint Monitoring Programme in 2022 (13).

Abbreviations: JMP, Joint Monitoring Programme; OD, open defecation; PRECISE, PREgnancy Care Integrating translational Science, Everywhere

^1^ Survey question did not specify whether soap and water were available in the household, so it was not possible to categorise PRECISE countries into the basic hygiene levels.
