# Supplementary material for: Evaluating the association between household water, sanitation and hygiene (WASH) and selected placenta-related complications in The Gambia, Kenya and Mozambique
Source: PLOS Glob Public Health. 2026 Oct 15;6(10):e0005004. doi: 10.1371/journal.pgph.0005004 (PMC13671507; doi:10.1371/journal.pgph.0005004)
Supplement: Supplementary file 6 [file pgph.0005004.s005.docx]

**S5 Table. Bivariable models (modified Poisson regression with robust standard errors) of eight WASH variables and their associations with selected placenta‑related complications in the PRECISE cohort in The Gambia, Kenya and Mozambique with imputed and non-imputed datasets.**

|  |  |  |  | **Imputed dataset** | | | **Non-imputed dataset** | | |
| --- | --- | --- | --- | --- | --- | --- | --- | --- | --- |
| **Variable** | **Overall,**  **N = 4,957** | **No selected placenta-related complication,**  **N = 3,622** | **Selected placenta-related complication, N = 1,335** | **RR** | **95% CI** | **p-value** | **RR** | **95% CI** | **p-value** |
| **Source of drinking water** |  |  |  |  |  |  |  |  |  |
| Piped water into dwelling/plot | 2,542 (51.4%) | 1,942 (53.7%) | 600 (45.1%) | Ref |  |  | Ref |  |  |
| Other improved water source | 2,122 (42.9%) | 1,472 (40.7%) | 650 (48.9%) | 1.30 | 1.18–1.43 | <0.001 | 1.30 | 1.18–1.43 | <0.001 |
| Unimproved water source | 280 (5.7%) | 200 (5.5%) | 80 (6.0%) | 1.21 | 0.99–1.48 | 0.057 | 1.21 | 0.99–1.48 | 0.059 |
| Unknown | 13 | 8 | 5 |  |  |  |  |  |  |
| **Source of non-drinking water** |  |  |  |  |  |  |  |  |  |
| Piped water into dwelling/plot | 2,554 (51.6%) | 1,959 (54.1%) | 595 (44.7%) | Ref |  |  | Ref |  |  |
| Other improved water source | 2,084 (42.1%) | 1,442 (39.9%) | 642 (48.3%) | 1.32 | 1.2–1.46 | <0.001 | 1.32 | 1.2–1.45 | <0.001 |
| Unimproved water source | 310 (6.3%) | 217 (6.0%) | 93 (7.0%) | 1.29 | 1.08–1.56 | 0.006 | 1.29 | 1.07–1.55 | 0.007 |
| Unknown | 9 | 4 | 5 |  |  |  |  |  |  |
| **Alternative water source for uses other than drinking** | 166 (3.4%) | 121 (3.3%) | 45 (3.4%) | 1.01 | 0.78–1.3 | 0.950 | 1.01 | 0.78–1.3 | 0.951 |
| Unknown | 13 | 8 | 5 |  |  |  |  |  |  |
| **Location of water source** |  |  |  |  |  |  |  |  |  |
| In own dwelling | 654 (13.2%) | 499 (13.8%) | 155 (11.6%) | Ref |  |  | Ref |  |  |
| In own yard or plot | 2,201 (44.4%) | 1,671 (46.1%) | 530 (39.8%) | 1.01 | 0.87–1.18 | 0.869 | 1.02 | 0.87–1.19 | 0.842 |
| Elsewhere | 2,098 (42.4%) | 1,451 (40.1%) | 647 (48.6%) | 1.30 | 1.12–1.51 | <0.001 | 1.30 | 1.12–1.51 | <0.001 |
| Unknown | 4 | 1 | 3 |  |  |  |  |  |  |
| **Unimproved sanitation facility - Yes** | 958 (19.4%) | 658 (18.2%) | 300 (22.6%) | 1.22 | 1.09–1.35 | <0.001 | 1.22 | 1.09–1.35 | <0.001 |
| Unknown | 18 | 9 | 9 |  |  |  |  |  |  |
| **Sanitation facility location** |  |  |  |  |  |  |  |  |  |
| In own dwelling | 1,004 (20.3%) | 749 (20.7%) | 255 (19.1%) | Ref |  |  | Ref |  |  |
| In own yard or plot | 3,770 (76.1%) | 2,743 (75.8%) | 1,027 (77.1%) | 1.07 | 0.95–1.21 | 0.245 | 1.07 | 0.95–1.21 | 0.245 |
| Elsewhere | 178 (3.6%) | 128 (3.5%) | 50 (3.8%) | 1.11 | 0.86–1.44 | 0.427 | 1.11 | 0.85–1.43 | 0.444 |
| Unknown | 5 | 2 | 3 |  |  |  |  |  |  |
| **Share sanitation facility with non-household members - Yes** | 1,737 (35.1%) | 1,230 (34.0%) | 507 (38.1%) | 1.14 | 1.04–1.25 | 0.007 | 1.14 | 1.04–1.25 | 0.007 |
| Unknown | 9 | 5 | 4 |  |  |  |  |  |  |
| **Handwashing facility** |  |  |  |  |  |  |  |  |  |
| Fixed facility (sink/tap) in dwelling | 400 (8.1%) | 309 (8.6%) | 91 (6.9%) | Ref |  |  | Ref |  |  |
| Fixed facility (sink/tap) in yard/plot | 267 (5.4%) | 189 (5.3%) | 78 (5.9%) | 1.28 | 0.98–1.65 | 0.066 | 1.28 | 0.99–1.66 | 0.059 |
| Mobile object (bucket/jug/kettle) | 3,879 (78.8%) | 2,841 (78.9%) | 1,038 (78.6%) | 1.17 | 0.97–1.41 | 0.098 | 1.18 | 0.97–1.42 | 0.090 |
| No handwashing place in dwelling or yard/plot | 374 (7.6%) | 260 (7.2%) | 114 (8.6%) | 1.33 | 1.05–1.68 | 0.018 | 1.34 | 1.06–1.70 | 0.015 |
| Unknown | 37 | 23 | 14 |  |  |  |  |  |  |

Note: Unknown variables correspond to the non-imputed dataset

Abbreviations: CI, confidence interval; RR, relative risk; PRECISE, PREgnancy Care Integrating translational Science, Everywhere; Ref, reference group
