# Supplementary material for: Evaluating the association between household water, sanitation and hygiene (WASH) and selected placenta-related complications in The Gambia, Kenya and Mozambique
Source: PLOS Glob Public Health. 2026 Oct 15;6(10):e0005004. doi: 10.1371/journal.pgph.0005004 (PMC13671507; doi:10.1371/journal.pgph.0005004)
Supplement: Supplementary file 7 [file pgph.0005004.s006.docx]

**S6 Table. Final selected multivariable modified Poisson regression of retained WASH variables and their associations with selected placenta‑related complications in the PRECISE cohort in The Gambia, Kenya and Mozambique, with imputed and non-imputed datasets.**

|  | **Imputed dataset (N = 4,957)** | | | **Non-imputed dataset (N = 4,746)** | | |
| --- | --- | --- | --- | --- | --- | --- |
| **Variable** | **aRR** | **95% CI** | **p-value** | **aRR** | **95% CI** | **p-value** |
| **Source of drinking water** |  |  |  |  |  |  |
| Piped water into dwelling/plot | Ref |  |  | Ref |  |  |
| Other improved water source | 1.18 | 1.07–1.31 | 0.002 | 1.19 | 1.07–1.33 | 0.002 |
| Unimproved water source | 1.23 | 1.00–1.50 | 0.049 | 1.09 | 0.87–1.37 | 0.450 |
| **Unimproved sanitation facility** | 1.15 | 1.02–1.30 | 0.019 | 1.11 | 0.98–1.26 | 0.107 |

Note: adjusted for age at enrolment, parity, average MUAC <23 cm (Underweight), household located in rural area, education, % likelihood below the poverty line and season at conception.

Abbreviations: aRR, adjusted relative risk; CI, confidence interval; MUAC, mid-upper arm circumference; PRECISE, PREgnancy Care Integrating translational Science, Everywhere; Ref, Reference group
