# Supplementary material for: Evaluating the association between household water, sanitation and hygiene (WASH) and selected placenta-related complications in The Gambia, Kenya and Mozambique
Source: PLOS Glob Public Health. 2026 Oct 15;6(10):e0005004. doi: 10.1371/journal.pgph.0005004 (PMC13671507; doi:10.1371/journal.pgph.0005004)
Supplement: Supplementary file 8 [file pgph.0005004.s007.docx]

**S7 Table. Full multivariable modified Poisson regression model estimating associations between household WASH exposures, covariates, and selected placenta‑related complications in the PRECISE cohort.**

| **Variable** | **aRR** | **95% CI** | **p-value** |
| --- | --- | --- | --- |
| **Source of drinking water** |  |  |  |
| Piped water into dwelling/plot | Ref |  |  |
| Other improved water source | 1.18 | 1.07–1.31 | 0.002 |
| Unimproved water source | 1.23 | 1.00–1.50 | 0.049 |
| **Unimproved sanitation facility (Ref: Improved)** | 1.15 | 1.02–1.30 | 0.019 |
| **Age at enrolment** | 1.02 | 1.01–1.03 | <0.001 |
| **Highest education level** |  |  |  |
| None | Ref |  |  |
| Primary | 1.01 | 0.87–1.17 | 0.922 |
| Secondary | 0.92 | 0.78–1.08 | 0.314 |
| Higher | 0.86 | 0.68–1.08 | 0.192 |
| **% likelihood below the poverty line** | 1.00 | 1.00–1.00 | 0.644 |
| **Parity** |  |  |  |
| 0 | Ref |  |  |
| 1 | 0.63 | 0.55–0.72 | <0.001 |
| 2-4 | 0.60 | 0.52–0.69 | <0.001 |
| 5+ | 0.59 | 0.47–0.74 | <0.001 |
| **MUAC Underweight (<23) (Ref: MUAC ≥ 23)** | 1.01 | 0.88–1.17 | 0.884 |
| **Household location – Rural (Ref: Urban/peri-urban)** | 0.93 | 0.82–1.05 | 0.215 |
| **Conception season – Wet (Ref: Dry)** | 1.01 | 0.91–1.12 | 0.861 |
| **Country** |  |  |  |
| The Gambia | Ref |  |  |
| Kenya | 0.77 | 0.65–0.91 | 0.002 |
| Mozambique | 0.69 | 0.58–0.81 | <0.001 |

Abbreviations: aRR, adjusted relative risk; CI, confidence interval; PRECISE, PREgnancy Care Integrating translational Science, Everywhere; Ref, Reference group
