# Supplementary material for: Evaluating the association between household water, sanitation and hygiene (WASH) and selected placenta-related complications in The Gambia, Kenya and Mozambique
Source: PLOS Glob Public Health. 2026 Oct 15;6(10):e0005004. doi: 10.1371/journal.pgph.0005004 (PMC13671507; doi:10.1371/journal.pgph.0005004)
Supplement: Supplementary file 9 [file pgph.0005004.s008.docx]

**S8 Table. Sensitivity analysis comparing drinking-water and non-drinking-water exposures and their associations with selected placenta‑related complications in the PRECISE cohort.**

| **Model specification** | **Water-source category** | **aRR** | **95% CI** | **p-value** |
| --- | --- | --- | --- | --- |
| **Drinking-water source** | Piped into dwelling/plot | Ref |  |  |
|  | Other improved source | **1.18** | **1.07–1.31** | **0.002** |
|  | Unimproved source | **1.23** | **1.00–1.50** | **0.049** |
| **Non-drinking-water source** | Piped into dwelling/plot | Ref |  |  |
|  | Other improved source | 1.22 | 1.10–1.36 | <0.001 |
|  | Unimproved source | 1.30 | 1.08–1.57 | 0.006 |

Note: adjusted for age at enrolment, parity, average MUAC <23 cm (Underweight), household located in rural area, education, % likelihood below the poverty line and season at conception.

Abbreviations: aRR, adjusted relative risk; CI, confidence interval; MUAC, mid-upper arm circumference; Ref, reference group
