# Supplementary material for: Evaluating the association between household water, sanitation and hygiene (WASH) and selected placenta-related complications in The Gambia, Kenya and Mozambique
Source: PLOS Glob Public Health. 2026 Oct 15;6(10):e0005004. doi: 10.1371/journal.pgph.0005004 (PMC13671507; doi:10.1371/journal.pgph.0005004)
Supplement: Supplementary file 10 [file pgph.0005004.s009.docx]

**S9 Table. Comparison of the adjusted final multivariable model for the PRECISE cohort by country (The Gambia, Kenya and Mozambique).**

|  | **The Gambia (N = 898)** | | | **Kenya (N = 2,268)** | | | **Mozambique (N = 1,791)** | | |
| --- | --- | --- | --- | --- | --- | --- | --- | --- | --- |
| **Variable** | **aRR** | **95% CI** | **p-value** | **aRR** | **95% CI** | **p-value** | **aRR** | **95% CI** | **p-value** |
| **Source of drinking water** |  |  |  |  |  |  |  |  |  |
| Piped water into dwelling/plot | Ref |  |  | Ref |  |  | Ref |  |  |
| Other improved water source | 1.31 | 1.01–1.70 | 0.042 | 1.22 | 1.05–1.42 | 0.008 | 1.07 | 0.87–1.33 | 0.511 |
| Unimproved water source | 1.01 | 0.52–1.97 | 0.983 | 1.66 | 1.20–2.29 | 0.002 | 1.09 | 0.82–1.44 | 0.570 |
| **Unimproved sanitation facility** | 0.99 | 0.82–1.21 | 0.949 | 1.17 | 0.95–1.44 | 0.140 | 1.28 | 1.04–1.57 | 0.019 |

Note: adjusted for age at enrolment, parity, average MUAC <23 cm (Underweight), household located in rural area, education, % likelihood below the poverty line and season at conception. Analytic Ns reflect complete cases for outcomes and covariates in country‑specific models. Formal tests of interaction with country provided no clear evidence of effect modification for drinking-water source (χ²[4]=6.18; p=0.186) or unimproved sanitation (χ²[2]=3.37; p=0.186).

Abbreviations: aRR, adjusted relative risk; CI, confidence interval; MUAC, mid-upper arm circumference; PRECISE, PREgnancy Care Integrating translational Science, Everywhere; Ref, reference group
