# Supplementary material for: Evaluating the association between household water, sanitation and hygiene (WASH) and selected placenta-related complications in The Gambia, Kenya and Mozambique
Source: PLOS Glob Public Health. 2026 Oct 15;6(10):e0005004. doi: 10.1371/journal.pgph.0005004 (PMC13671507; doi:10.1371/journal.pgph.0005004)
Supplement: Supplementary file 11 [file pgph.0005004.s010.docx]

**S10 Table. Comparison of the adjusted final multivariable model for the PRECISE cohort by pregnancy complication.**

|  | **Pre-eclampsia (N = 5,738)** | | | **SGA (N = 4,957)** | | | **Stillbirth (N = 5,745)** | | |
| --- | --- | --- | --- | --- | --- | --- | --- | --- | --- |
| **Variable** | **aRR** | **95% CI** | **p-value** | **aRR** | **95% CI** | **p-value** | **aRR** | **95% CI** | **p-value** |
| **Source of drinking water** |  |  |  |  |  |  |  |  |  |
| Piped water into dwelling/plot | Ref |  |  | Ref |  |  | Ref |  |  |
| Other improved water source | 1.20 | 0.98–1.48 | 0.077 | 1.15 | 1.01–1.32 | 0.030 | 1.24 | 0.89–1.72 | 0.208 |
| Unimproved water source | 1.71 | 1.14–2.57 | 0.010 | 1.10 | 0.85–1.41 | 0.462 | 1.57 | 0.89–2.77 | 0.117 |
| **Unimproved sanitation facility** | 1.10 | 0.88–1.38 | 0.406 | 1.21 | 1.05–1.40 | 0.010 | 1.05 | 0.73–1.51 | 0.800 |

Note: adjusted for: age at enrolment, parity, average MUAC <23 cm (Underweight), household located in rural area, education, % likelihood below the poverty line, season at conception and country. Analytic Ns reflect complete cases for outcomes and covariates.

Abbreviations: aRR, adjusted relative risk; CI, confidence interval; MUAC, mid-upper arm circumference; PRECISE, PREgnancy Care Integrating translational Science, Everywhere; SGA, small-for-gestational-age; Ref, reference group
