# Supplementary material for: Evaluating the association between household water, sanitation and hygiene (WASH) and selected placenta-related complications in The Gambia, Kenya and Mozambique
Source: PLOS Glob Public Health. 2026 Oct 15;6(10):e0005004. doi: 10.1371/journal.pgph.0005004 (PMC13671507; doi:10.1371/journal.pgph.0005004)
Supplement: Supplementary file 14 [file pgph.0005004.s013.docx]

**S13 Table. Propensity‑score–matched modified Poisson regression models (with robust standard errors) estimating associations between household WASH exposures and individual placenta‑related complications in the PRECISE cohort (The Gambia, Kenya and Mozambique).**

| **Exposure** | **Pre-eclampsia** | | | | | **SGA** | | | | | **Stillbirth** | | | | |
| --- | --- | --- | --- | --- | --- | --- | --- | --- | --- | --- | --- | --- | --- | --- | --- |
|  | **RR** | **95% CI** | **p-value** | **Matched N** | **SMD before/after matching** | **RR** | **95% CI** | **p-value** | **Matched N** | **SMD before/after matching** | **RR** | **95% CI** | **p-value** | **Matched N** | **SMD before/after matching** |
| Other improved and unimproved vs piped into dwelling plot water | 1.23 | 0.97–1.57 | 0.089 | 3560 | 0.274/ 0.058 | 1.26 | 1.09–1.46 | 0.002 | 3080 | 0.285/ 0.057 | 1.32 | 0.89–1.97 | 0.165 | 3560 | 0.274/ 0.058 |
| Unimproved vs improved sanitation | 1.00 | 0.75–1.33 | 1.000 | 2116 | 0.300/ 0.036 | 1.17 | 0.98–1.39 | 0.086 | 1806 | 0.293/ 0.031 | 0.95 | 0.61–1.48 | 0.816 | 2116 | 0.300/ 0.036 |

Note: Propensity scores were estimated using logistic regression including age at enrolment, parity and average MUAC represented categorically, together with rural residence, education, household poverty-likelihood quintile and season at conception. We performed 1:1 nearest-neighbour matching without replacement, estimating the average treatment effect on the treated (ATT), with exact matching on country and a calliper of 0.2 standard deviations on the logit of the propensity score. Observations outside the region of common support were discarded. Relative risks were estimated using modified Poisson regression with robust standard errors, clustering by matched subclass and incorporating matching weights. Reported SMDs represent the maximum absolute SMD across measured covariates before and after matching. All measured covariates achieved SMDs below 0.10 after matching.

Abbreviations: CI, confidence interval; RR, relative risk; MUAC, mid-upper arm circumference; PRECISE, PREgnancy Care Integrating translational Science, Everywhere; SGA, small-for-gestational-age; SMD, standardised mean difference
