# Supplementary material for: Evaluating the association between household water, sanitation and hygiene (WASH) and selected placenta-related complications in The Gambia, Kenya and Mozambique
Source: PLOS Glob Public Health. 2026 Oct 15;6(10):e0005004. doi: 10.1371/journal.pgph.0005004 (PMC13671507; doi:10.1371/journal.pgph.0005004)
Supplement: Supplementary file 16 [file pgph.0005004.s019.docx]

**S1 Acknowledgements. Membership of the PRECISE and PRECISE-DYAD Networks.**

Marleen Temmerman, Angela Koech, Patricia Okiro, Geoffrey Omuse, Consolata Juma, Joseph Mutunga, Moses Mukhanya, Isaac Mwaniki, Marvin Ochieng, Onesmus Wanje, Emily Mwadime, Amina Abubakar, Umberto D’Alessandro, Anna Roca, Hawanatu Jah, Andrew Prentice, Melisa Martinez-Alvarez, Brahima Diallo, Abdul Sesay, Sambou Suso, Yahaya Idris, Baboucarr Njie, Fatima Touray, Fatoumata Kongira, Modou F.S. Ndure, Gibril Gabbidon, Lawrence Gibba, Abdoulie Bah, Yorro Bah, Esperança Sevene, Corssino Tchavana, Salesio Macuacua, Anifa Vala, Helena Boene, Lazaro Quimice, Sonia Maculuve, Inacio Mandomando, Peter von Dadelszen, Laura A. Magee, Rachel Craik, Marie-Laure Volvert, Hiten Mistry, Donna Russell, Prestige Tatenda Makanga, Liberty Makacha, Reason Mlambo, Lucilla Poston, Rachel Tribe, Sophie Moore, Tatiana Salisbury, Aris Papageorghiou, Alison Noble, Hannah Blencowe, Veronique Filippi, Joy Lawn, Matt Silver, Joseph Akuze, Ursula Gazeley, Judith Cartwright, Guy Whitley, Sanjeev Krishna, Marianne Vidler, Jing (Larry) Li, Jeff Bone, Mai-Lei (Maggie) Woo Kinshella, Domena Tu, Ash Sandhu, Kelly Pickerill, Carla Carrilho, Benjamin Barratt, Akbar Waljee, Jovito Nunes, Valdemiro Escola, Charfudin Sacoor, Cristina Escalona, Anne Rerimoi, Giulia Ghillia, Thomas Mendy, Jaya Chandna, Asma Khalil, Akshdeep Sandhu, Melissa Gladstone, Dorcas Magai and Cathryn Tonne.
